# Gene co-expression network analysis in *Rhodobacter capsulatus* and application to comparative expression analysis of *Rhodobacter sphaeroides*

**DOI:** 10.1186/1471-2164-15-730

**Published:** 2014-08-28

**Authors:** Lourdes Peña-Castillo, Ryan G Mercer, Anastasia Gurinovich, Stephen J Callister, Aaron T Wright, Alexander B Westbye, J Thomas Beatty, Andrew S Lang

**Affiliations:** Department of Biology, Memorial University of Newfoundland, St. John’s, NL A1B 3X5 Canada; Department of Computer Science, Memorial University of Newfoundland, St. John’s, NL Canada; Biological Sciences Division, Pacific Northwest National Laboratory, Richland, WA 99352 USA; Department of Microbiology and Immunology, University of British Columbia, Vancouver, BC Canada

**Keywords:** Comparative transcriptomics, Module preservation, Gene-protein expression conservation, *Rhodobacter capsulatus*, *Rhodobacter sphaeroides*

## Abstract

**Background:**

The genus *Rhodobacter* contains purple nonsulfur bacteria found mostly in freshwater environments. Representative strains of two *Rhodobacter* species, *R. capsulatus* and *R. sphaeroides*, have had their genomes fully sequenced and both have been the subject of transcriptional profiling studies. Gene co-expression networks can be used to identify modules of genes with similar expression profiles. Functional analysis of gene modules can then associate co-expressed genes with biological pathways, and network statistics can determine the degree of module preservation in related networks. In this paper, we constructed an *R. capsulatus* gene co-expression network, performed functional analysis of identified gene modules, and investigated preservation of these modules in *R. capsulatus* proteomics data and in *R. sphaeroides* transcriptomics data.

**Results:**

The analysis identified 40 gene co-expression modules in *R. capsulatus*. Investigation of the module gene contents and expression profiles revealed patterns that were validated based on previous studies supporting the biological relevance of these modules. We identified two *R. capsulatus* gene modules preserved in the protein abundance data. We also identified several gene modules preserved between both *Rhodobacter* species, which indicate that these cellular processes are conserved between the species and are candidates for functional information transfer between species. Many gene modules were non-preserved, providing insight into processes that differentiate the two species. In addition, using Local Network Similarity (LNS), a recently proposed metric for expression divergence, we assessed the expression conservation of between-species pairs of orthologs, and within-species gene-protein expression profiles.

**Conclusions:**

Our analyses provide new sources of information for functional annotation in *R. capsulatus* because uncharacterized genes in modules are now connected with groups of genes that constitute a joint functional annotation. We identified *R. capsulatus* modules enriched with genes for ribosomal proteins, porphyrin and bacteriochlorophyll anabolism, and biosynthesis of secondary metabolites to be preserved in *R. sphaeroides* whereas modules related to RcGTA production and signalling showed lack of preservation in *R. sphaeroides.* In addition, we demonstrated that network statistics may also be applied within-species to identify congruence between mRNA expression and protein abundance data for which simple correlation measurements have previously had mixed results.

**Electronic supplementary material:**

The online version of this article (doi:10.1186/1471-2164-15-730) contains supplementary material, which is available to authorized users.

## Background

Species in the genus *Rhodobacter* are purple nonsulfur bacteria found mostly in freshwater environments [1]. A hallmark of purple nonsulfur bacteria is that they display tremendous physiological diversity [2]. Genome sequences are available from two *Rhodobacter* species, *R. capsulatus* [3] and *R. sphaeroides* [4], and transcriptional profiling studies have been performed with both species [5–8]. These two species have been widely studied as model organisms for anoxygenic photosynthesis, carbon and nitrogen fixation, chemotaxis and flagellar motility, and various regulatory systems including quorum sensing, two-component phosphorelays and those responsible for regulation in response to O_2_ and light [9–12] *. R. capsulatus* is also a model organism for study of a gene transfer agent, RcGTA, which is a virus-like particle that packages small segments of the genome of a GTA-producing cell that can then be transferred to recipient cells [13].

Weighted gene co-expression network analysis (WGCNA) has been widely used to analyze transcriptional profiles since its introduction in 2005 [14, 15], and has proved to be a useful approach for the functional annotation of uncharacterized genes [16, 17]. In a recent critical assessment of methods for constructing gene networks [18] WGCNA was found to be one of the methods that performed the best for constructing global co-expression networks. After network construction, functional analysis focuses on groups of tightly connected genes (known as modules) instead of single genes. Because genes within the same modules tend to maintain a consistent, correlated expression relationship independent of phenotype or experimental condition, such genes are assumed to be functionally associated, and shared regulatory and/or functional pathways may be inferred. In addition, WGCNA offers functionality to assess whether gene modules are preserved in other networks [19]. Preserved gene modules indicate biological processes that are conserved between species and may be candidates for functional information transfer between species. Non-preserved gene modules reflect species-specific modules, which may provide insight into biological processes that have diverged between species. Recently, a metric for expression divergence called Local Network Similarity (LNS) was proposed to assess expression conservation of a pair of orthologs [20]. LNS is the correlation between the correlations of the pair of orthologs’ expression and the expression patterns of all other identified orthologs. This metric differs from the module preservation statistics obtained by WGCNA in that it is applied to a pair of genes instead of to a gene module. LNS and WGCNA may also be applied to diverse datasets such as mRNA expression and protein abundance data. Observations of low to moderate correlations between mRNA expression and protein abundance data are recurrent in the literature [21, 22], indicating that network-based metrics of similarity may be more suitable to compare these two types of data.

In this study, we constructed an *R. capsulatus* gene co-expression network, and took advantage of the module preservation functionality in WGCNA to identify *R. capsulatus* gene modules preserved in a collection of published *R. sphaeroides* mRNA expression data, and in a *R. capsulatus* proteomics dataset. In addition, we calculated LNS for all 2175 pairs of orthologs between the two *Rhodobacter* species, and we also applied this metric to assess whether *R. capsulatus* genes and proteins have similar co-expression relationships in the protein abundance and mRNA expression data. We also related LNS to WGCNA module preservation statistics and investigated the effect of the size of the datasets in LNS. In sum, we produced comparative transcriptomics resources to guide further functional studies of *R. capsulatus*, and, to the best of our knowledge, performed the first application of network-based expression preservation metrics between transcriptomics and proteomics data.

## Results and discussion

### *R. capsulatus*co-expression network

We used 48 gene expression experiments encompassing 23 different conditions and/or mutant strains for the 3571 genes on the *R. capsulatus* microarrays to construct a gene co-expression network using WGCNA. A total of 40 gene co-expression modules were identified. To assess the stability of modules, we performed a resampling analysis of cluster robustness as described in [23]. The results of cluster stability analysis indicated that module assignments were reasonably stable with many of the modules being identified in most resampled data sets (see Additional file 1). The modules varied in size from 18 to 696 genes with an average size of 87 genes. A total of 3,533 genes out of the 3,571 genes represented on the microarrays were assigned to modules. Thirty-seven modules had enrichment of at least one type of biological gene set (i.e., protein domain, biological pathway, protein complex or transcription unit), and 21 modules were related to at least one biological pathway, which indicated that the modules were biologically meaningful. Some modules of interest are discussed below to illustrate the validity of this analysis.

**Figure 1:**
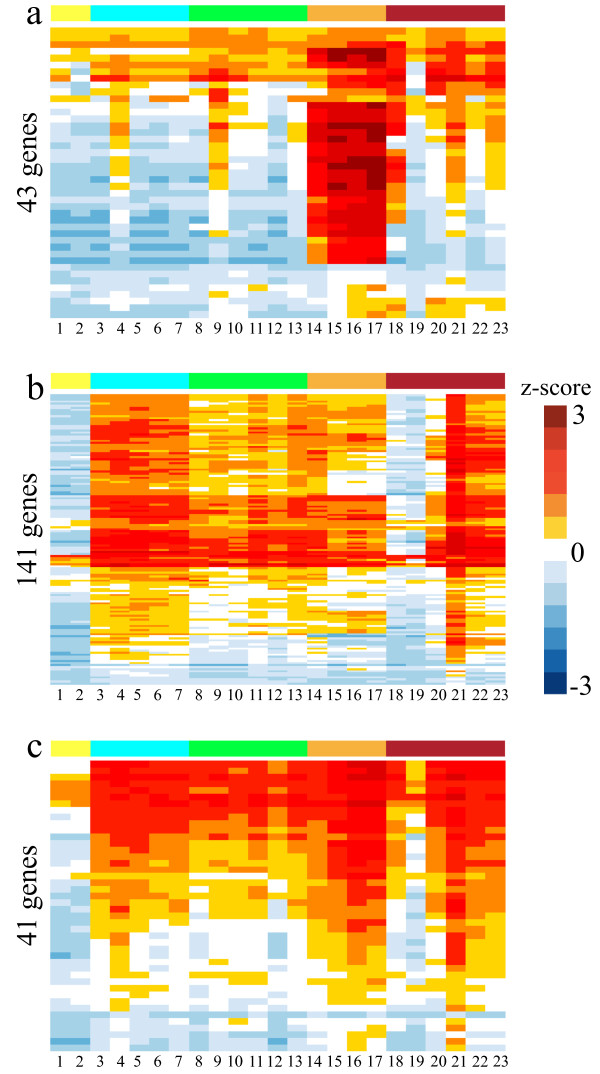
**Expression profiles of genes in selected co-expression modules across all conditions and/or mutant strains.** Heatmap colours indicate robust z-scores. Colours on top of the columns refer to clusters of conditions/mutant strains highlighted in Figure 2. Numbers below the columns correspond to the indices of the conditions mutant strains described in Table 1. **(a)** Orange co-expression module; **(b)** Pink co-expression module; **(c)** Darkturquoise co-expression module.

One gene co-expression module containing 43 genes (the orange module) was associated with the production of RcGTA. This module was enriched (p-value = 5.8e-35) with the RcGTA gene cluster (*rcc01682* to *rcc01698*) [24]. It also contained the endolysin and holin genes (*rcc00555* and *rcc00556*) required for RcGTA release [25, 26], and genes predicted to be involved in DNA uptake and recombination, with two genes annotated as related to competence (*comM* and *rcc02362*) and three genes associated with DNA repair and protection and incorporation of DNA received from RcGTA particles (*radC*, *recA* and *dprA*) [27]. There were also two genes encoding predicted signal transduction proteins, *rcc00042* encoding a sensor domain protein and *rcc00645* encoding a diguanylate cyclase/phosphodiesterase, which had previously been identified as affected by the loss of the response regulator CtrA similar to the RcGTA gene cluster [5]. The trends for genes in this module were increased expression in the stationary phase relative to logarithmic phase, reduced expression in the *ctrA* and *gtaI* mutants but not in the *cckA* mutant, and greatly increased expression in the RcGTA overproducer strain, DE442 (Figure 1a).

Signal transduction and transcriptional regulation proteins affected by the loss of the response regulator protein CtrA [5] are significantly over-represented (p-value = 4.8e-22) among the 141 genes forming the pink module, with 17 out of the 23 previously identified proteins in this module. The pink module showed a significant enrichment of genes involved in chemotaxis (FDR-corrected p-value of 1.3e-34), two-component systems (FDR-corrected p-value of 8.6e-11), and flagellar assembly (FDR-corrected p-value of 2.9e-9). This module also contains all 17 *R. capsulatus* proteins containing a Methyl-accepting chemotaxis protein (MCP) signalling domain (FDR-corrected p-value of 1.7e-20). Genes in the pink module showed significantly lower expression in both the *cckA* and *ctrA* strains (Figure 1b). This corresponds to previous work that demonstrated that CtrA and CckA are required for expression of flagellar and chemotaxis genes [5, 28, 29]. Genes within this module have also been shown to be involved in control of motility [28] and expression of the RcGTA genes (*rbaU*, *rbaV* and *rbaW*; [30]). The darkturquoise module also contained an over-representation of flagellar genes (FDR-corrected p-value of 6.3e-11), and visually showed a very similar expression profile (Figure 1c) as the pink module. The one exception was that expression of genes in the darkturquoise module was elevated in the DE442 strain in the transition and stationary growth phases while expression of genes in the pink module was not. The median expression profiles of the pink and darkturquoise modules reciprocally correlate the most with each other (Pearson correlation of 0.76, p-value = 2.87e-5). Correlations between module median expression profiles are shown in Additional file 2. In addition to flagellar genes, the darkturquoise module contained an over-representation of gas vesicle genes. In total, the orange, pink and darkturquoise modules represent 84% of what was previously identified as the CtrA “regulon” [5].

Several modules showed patterns of expression that were most affected by the culture growth medium (heatmaps illustrating the expression profiles of all modules are provided in Additional file 3). This included the darkred and orangered4 modules that showed a relative decrease in expression in RCV medium and the cyan, greenyellow and paleturquoise modules that showed increased expression in RCV medium. Not surprisingly, these modules contained many genes involved in transport and various aspects of metabolism such as sugar and vitamin biochemistry.

Two modules, midnightblue and salmon4, which showed high relative expression across all strains and conditions contained many of the genes required for phototrophic growth. This included genes encoding the photosynthetic reaction centre, the light-harvesting complexes I and II, and bacetriochlorophyll and carotenoid pigment biosynthesis proteins. High expression of these genes is expected because all RNA samples used in the microarray experiments came from cultures grown photoheterotrophically, and these genes are well characterized for their global regulation by several key regulators [31].

The darkgreen module contained genes responsible for synthesis of the capsule, *rcc0181-01086* and *rcc01958-01960*, required as an RcGTA receptor [32]. This module showed increased expression in strain DE442 and decreased gene expression in the *gtaI* mutant in the logarithmic phase when grown in RCV medium. This module also contains one of the *R. capsulatus* crispr-associated (*cas*) gene clusters [3]. The skyblue3 module could also be implicated as affected by quorum sensing because of decreased expression levels in the *gtaI* mutant relative to wild type (in the logarithmic phase only). This module included another one of the genes required for capsule synthesis, *rcc01932* [32], and *rcc01955-01957*, which are located adjacent to the genes in the darkgreen module mentioned above that are involved in quorum sensing-dependent capsule production.

Three modules, skyblue, turquoise and violet, showed lower relative expression across all strains and conditions. These modules obviously represent genes with low or no expression under the conditions of these experiments, and the turquoise module was the largest of all 40 modules, with 696 genes. Of note in the turquoise module is a large number of prophage genes, representing 5 distinct uncharacterized prophage regions as well as the majority of the genes of RcapMu [33]. This module also includes genes for nitrogen fixation and several alternative sigma factors.

As a result of this gene co-expression network analysis, 99% of the 909 *R. capsulatus* genes described as “hypothetical protein” were assigned to modules. These uncharacterized genes might now be putatively implicated in specific biological processes to guide functional characterization. Gene module assignments are provided in Additional file 4.

We also tested whether genes in certain modules were preferentially packaged in the RcGTA particles using the available RcGTA packaging microarray data [25]. No modules were found to be over-represented in the RcGTA-packed DNA but we observed a strong inverse correlation (Pearson correlation of -0.84, p-value = 2.35e-12) between the content of plasmid genes in a module and the intensity measurements detected in the RcGTA DNA. This is expected as the RcGTA DNA was isolated from DE442, which lacks the ~100-kb plasmid present in the genome-sequenced strain, SB1003 [25]. Although the plasmid genes were distributed amongst 17 different modules, the darkmagenta module showed the largest proportional plasmid gene content (18/30) of all co-expression modules, at 60% plasmid-borne genes. The darkmagenta and three other modules that contained >15 plasmid genes, purple (17), royalblue (25) and turquoise (31), combined to contain 64% of all plasmid genes on the arrays, indicating widespread co-regulation of the plasmid-borne genes.

To confirm that the modules with a large number of plasmid genes were still identifiable in the absence of the samples from the plasmid-lacking DE442 strain, we assessed (using the WGCNA modulePreservation function) whether such modules were reproducible in the data subset without the DE442 data. Indeed, there was moderate to strong evidence (Zsummary.pres > 5) that all 40 modules were present in the subset of data without the DE442 samples. Thus, modules are robust to the lack of signal for the plasmid genes in the DE442 data.

**Figure 2:**
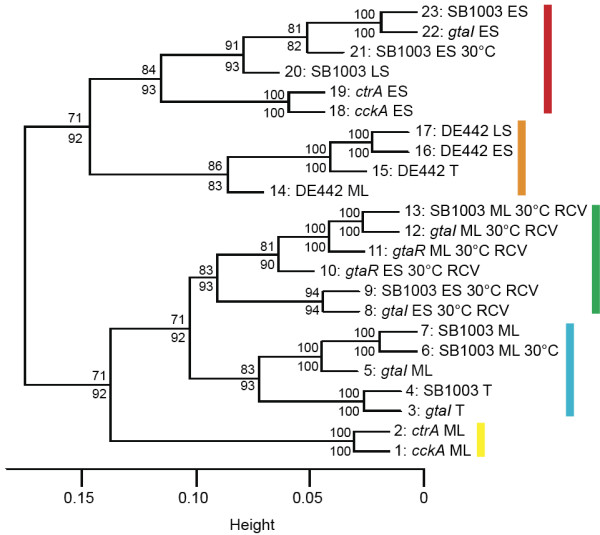
**Dendrogram showing hierarchical clustering of conditions and/or mutant strains based on gene expression profiles.** Values at branches are approximately unbiased (AU) percentage values (bottom) and bootstrap (BP) percentage values (top). BP values indicate the percentage of bootstrap replicates in which a given cluster appears. Leaf numbers correspond to the indices of the conditions/mutant strains described in Table 1. Coloured boxes indicate branches in the dendrogram of conditions/mutant strains having in common RCV growth medium (green), the DE442 strain (orange), and culture growth phase (yellow and blue versus red).

To explore similarity of expression profiles based on conditions and/or mutant strains, we performed a hierarchical cluster (average linkage) analysis with multiscale bootstrap resampling (ten different sample sizes and 10000 bootstrap samples) [34] using the Pvclust R package (version 1.2.2) [35]. The dendrogram obtained (Figure 2) indicated that gene expression profiles form groups based on RCV growth medium (green), the DE442 strain (orange), and culture growth phase (yellow and blue versus red).

### Network based comparison of transcript levels and protein abundance

We explored the preservation of co-expression between *R. capsulatus* mRNAs and proteins by applying the modulePreservation function from WGCNA. This function, using a permutation test, assessed whether the module nodes identified in *R. capsulatus* gene co-expression network remained connected in the protein co-expression network and whether the connectivity pattern between nodes in both networks was similar. A composite preservation statistic, Zsummary, can be used to evaluate whether modules are preserved. A Zsummary > 2 indicates that there is weak to moderate evidence of preservation and Zsummary > 10 indicates that there is strong evidence that the module is preserved [19]. Note that module preservation can be assessed using the protein co-expression network (as defined by a correlation matrix) without cluster detection. We realized that the small sample size of the proteomics dataset (six conditions and 1158 proteins) might reduce the statistical power to pinpoint preserved modules; however, it seemed possible that strongly conserved biological signals could be identified. Indeed, we found evidence of preservation of two gene modules in the protein abundance data: the blue module (Zsummary = 2.90), which was enriched with a number of housekeeping functions (Additional file 5), and especially with ribosomal proteins (FDR-corrected p-value of 3.3e-38), and the brown module (Zsummary = 2.48), which was enriched in genes related to iron transport (FDR-corrected p-values <0.01) (Additional file 5). Additional file 6 shows preservation statistics of *R. capsulatus* mRNA modules in the protein co-expression network. This suggests that network-based analysis may be suitable for identifying preservation of global expression between transcriptomics and proteomics data. Comparison of these two data types has frequently yielded mixed results with reports of low to moderate correlations [21, 22]. A network-based analysis with a larger sample size of protein abundance data is needed to corroborate and further extend our finding.

### Comparative transcriptomics in *Rhodobacter*species

We investigated the preservation of global co-expression between *R. capsulatus* and *R. sphaeroides* using the network-based statistics calculated by the modulePreservation function from WGCNA. There are two main network-based statistics found to accurately distinguish preserved from unpreserved modules: Zsummary and medianRank [19]. These statistics are calculated twice: once to assess whether modules are reproducible in the reference data subset consisting only of genes in common with the test dataset (referred to as “quality” statistics), and the second time to evaluate the conservation of the modules in the test data subset (referred to as “preservation” statistics). The quality statistics are a complementary approach to the cluster stability analysis to assess the robustness of the identified modules. 2123 one-to-one orthologs between the species have been identified by Reciprocal Best Match [36], and we calculated the module preservation statistics in the data subsets containing these 2123 orthologous genes between the *R. capsulatus* (reference) and *R. sphaeroides* (test) co-expression networks.

**Figure 3:**
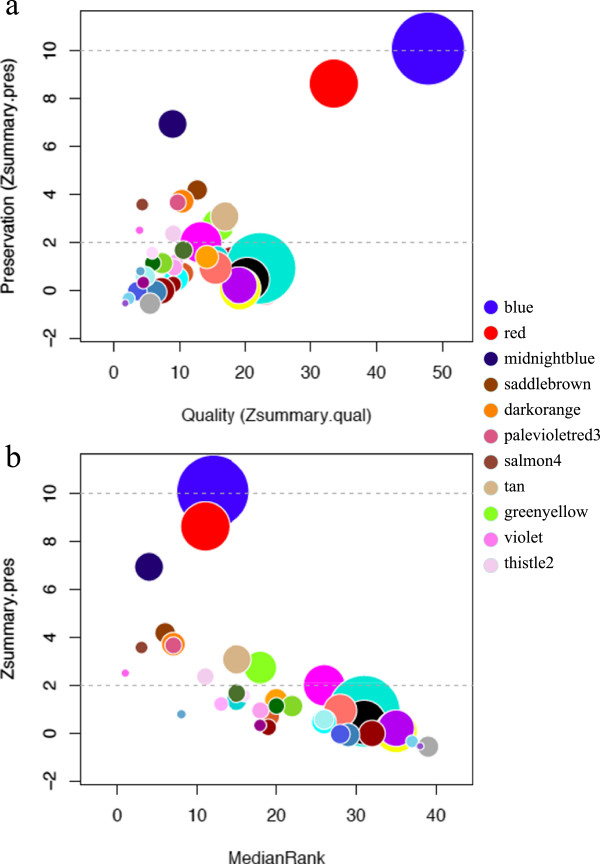
**Preservation statistics of** ***R. capsulatus*** **modules in** ***R. sphaeroides*** **data.** The size of the bubble represents module size in the data subset used to assess module preservation. The horizontal lines indicate the Zsummary.pres thresholds for strong evidence of conservation (above 10) and for low to moderate evidence of conservation (above 2). *R. capsulatus* modules found to be conserved in *R. sphaeroides* are listed on the right side. **(a)** Module preservation as a function of module quality; **(b)** relationship between the two preservation statistics (Zsummary.pres and medianRank). Lower medianRank indicates higher preservation.

The quality and preservation of the 40 *R. capsulatus* co-expression modules identified are illustrated in Figure 3a. Zsummary tends to be more dependent on the module size than medianRank [19]; nevertheless both statistics showed a strong correlation (Pearson coefficient of -0.654) assessing the preservation of *R. capsulatus* modules in *R. sphaeroides* data (Figure 3b). Unsurprisingly, there was strong evidence of preservation (Zsummary.pres > 10) of the blue module. We found low to moderate evidence of preservation (2 < Zsummary.pres < 10) for ten additional modules. Among those, there were modules enriched with proteins implicated in porphyrin and bacteriochlorophyll metabolism (midnightblue, FDR-corrected p-value of 1.16e-11), biosynthesis of secondary metabolites (red, FDR-corrected p-value of 7.9e-7), ABC transporters (tan, FDR-corrected p-value of 0.0001), CO_2_ fixation (darkorange, FDR-corrected p-value of 0.0003), two-component systems (salmon4, FDR-corrected p-value of 0.0007), protein secretion (palevioletred3, FDR-corrected p-value of 0.03), and lysine biosynthesis (thistle2, FDR-corrected p-value of 0.03).

The orange module, related to RcGTA production, is amongst those not preserved in *R. sphaeroides*. This is consistent with the fact that no evidence of GTA production has been found in *R. sphaeroides* [13], despite conservation of the GTA genes [37]. Other non-preserved modules were the pink (related to chemotaxis and signalling), darkturquoise (related to flagellar assembly), darkred (related to aerobic hydrogen oxidation), darkolivegreen (related to Fe^2+^ oxidation), green (related to adenosylcobalamin biosynthesis from cobyrinate a,c), turquoise (related to chloroalkane and chloroalkene degradation), sienna3 (related to valine metabolism), yellowgreen (related to creatinine degradation and formate oxidation), steelblue (related to biotin metabolism), and darkgreen (related to 2-ketoglutarate dehydrogenase complex). There was also no evidence of preservation of the brown module in *R. sphaeroides*, which was one of the conserved *R. capsulatus* mRNA-protein modules.

### Assessment of gene-wise conservation of expression

**Figure 4:**
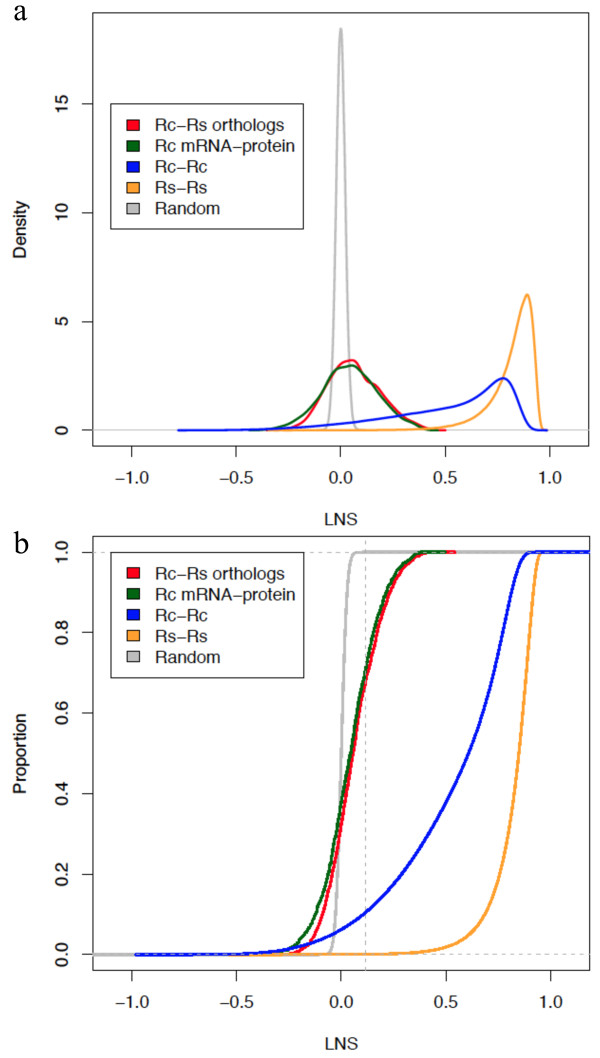
**Within-species and between-species expression conservation. (a)** Distribution of within-species and between-species LNS scores is shifted to right of the null distribution. Within-species show a stronger shift towards positives values; **(b)** represents the same data as in (a) but as the cumulative distribution. The vertical dashed line indicates the maximum LNS score observed in the null distribution.

In addition to evaluating module preservation between the two *Rhodobacter* species, we wanted to assess pairwise conservation of expression between orthologs. Therefore, we calculated Local Network Similarity (LNS) [20] to study the conservation of gene expression between the two species*.* This metric was developed for application to expression datasets consisting of unmatched experimental conditions, and it quantifies the similarity of the expression correlations between a pair of orthologs and all other identified orthologs. We decided to explore the effect of dataset size in LNS and obtained the LNS within each species by dividing the available data into two different subsets. We also simulated the null-hypothesis of no conservation by randomizing the ortholog pairs (see Methods). LNS scores of the null distribution ranged from -0.11 to 0.11 with an average very close to zero (2.6e-5). The within-species LNS distributions showed a pronounced shift towards positive values (Figure 4a). However, the *R. capsulatus* distribution was less positive (median LNS of 0.61) than that of *R. sphaeroides* (median LNS of 0.85)*.* This is likely due to the difference in the amounts of transcriptomics data for the two species, as the *R. sphaeroides* dataset contains eight times as many arrays as the *R. capsulatus* dataset.

The LNS was then calculated between *R. capsulatus* and *R. sphaeroides* orthologs and between the *R. capsulatus* transcriptomics and proteomics data (Figure 4). The between-species LNS scores of the matched ortholog pairs showed less positive values than the within-species LNS, but there were still values to the right of the null distribution such that 30% of ortholog pairs had a positive LNS score greater than 100% of the random values (Figure 4b). Co-expression of between-species ortholog pairs is expected to be less similar than the within-species co-expression. Furthermore, the orthologs’ functions may have diverged in the different species, in which case the LNS should be low to reflect this divergence. For example, the LNS scores of *R. capsulatus* genes involved in the production of RcGTA and their corresponding *R. sphaeroides* orthologs ranged from -0.08 to 0.10 while highly conserved housekeeping genes such as *aroA* and *radA* have LNS scores of 0.43 and 0.38, respectively. Encouragingly, the LNS scores between *R. capsulatus* mRNA expression and protein abundance data are also to the right of the null distribution suggesting that the LNS metric is sensitive enough to detect conservation of expression in small datasets and between diverse data types. LNS scores are provided in Additional file 7.

### Relationship between module preservation statistics

**Figure 5:**
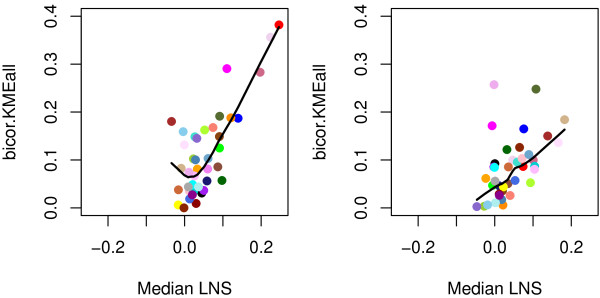
**Relationship between module preservation connectivity statistics.** Total network correlation of the module eigengenes connectivity (bicor.KMEall) as a function of the median-LNS per module for *R. capsulatus* modules in *R. sphaeroides* (left) and *R. capsulatus* mRNA modules in *R. capsulatus* proteomics data (right). Each point represents a module labeled by the colour corresponding to the module name. The black line is the loess smoothed line.

Connectivity statistics quantify whether connections between genes in the reference network are similar to those in the test network. By its definition, LNS is a connectivity-based metric. To relate LNS to WGCNA module preservation statistics, we obtained the median LNS per module (henceforth referred to as median-LNS). After comparing the median-LNS with WGCNA connectivity-based statistics, we found that median-LNS correlated best with bicor.kMEall, which is the correlation of the total network module eigengenes connectivity. A module eigengene (ME) summarizes the expression profile of a module. The relationship between LNS-Median and cor.kMEall is shown in Figure 5. We observed a Pearson correlation between median-LNS and cor.kMEall of 0.78 (p-value of 2.6e-9) for the network comparison between the two *Rhodobacter* species, and of 0.49 (p-value of 0.001) for the comparison between *R. capsulatus* mRNA and protein expression.

## Conclusions

Using WGCNA and functional analysis of *R. capsulatus* transcriptomics data, we identified distinct groups of co-expressed genes with associations to biological gene sets (protein domains, metabolic pathways, transcriptional units and/or protein complexes). We observed co-expression modules associated with functions known to be co-regulated based on previous studies, such as the production of RcGTA, motility and chemotaxis. These identified co-expression modules will be useful to identify candidate genes for further investigations in *R. capsulatus* biology, such as the regulation and production of RcGTA. In addition, we distinguished between preserved and non-preserved modules between *R. capsulatus* and *R. sphaeroides*. The module preservation results point to a lack of similarity between the two *Rhodobacter* species for many of the modules, whereas the expression of several metabolic pathways was similar in both species. We also quantified the conservation of expression of all one-to-one orthologs between these species using the LNS metric. These resources may aid in the identification of functional analog genes (those with conserved functional roles) in these bacteria, and comparative transcriptomics studies such as this can be applied to other bacterial species to obtain evidence of gene expression conservation and thereby allow further exploration of gene function.

## Methods

### Datasets

Putting together published [5, 25, 32] and unpublished microarray experiments (NCBI Gene Expression Omnibus database accessions: GSE18149, GSE33176, GSE41014 and GSE53636), we collected 48 gene expression experiments encompassing 23 different conditions and/or mutant strains for the 3571 genes on the *R. capsulatus* microarrays. We also analyzed a small-scale proteomics dataset of 1158 proteins for *R. capsulatus* over six conditions and/or mutant strains ([5], and our own unpublished data) and collected all data from 192 *R. sphaeroides* microarray experiments available in NCBI Gene Expression Omnibus (GEO) [38].

### *R. capsulatus*transcriptomics analysis

In addition to the previously published arrays [5, 25, 32], data were used from the strains and growth conditions described below. The complete listing of conditions and/or mutant strains used for the analyses is provided in Table 1 and the strains are described in Table 2. RNA isolations and hybridizations to the arrays were done as described in [5]; specifically, RNA was isolated using Qiagen RNeasy Minikit and cDNA synthesis, labelling and target hybridization performed as described in the Affymetrix Expression Analysis Technical Manual for prokaryotic samples. Arrays were quantile normalized together using the RMA method as implemented in the Affy package [39] for R (version 2.15.0). Quality tests were performed on the normalized array data using the Bioconductor AffyPLM package (version 1.36.0) [40], and by examining chip trees generated by the R WGCNA package (version 1.27.1) [41] and the Pvclust R package (version 1.2.2) [35].

**Table 1:** **List of conditions and/or mutant strains represented in** ***R. capsulatus*** **samples**

Module index^a^	Strain	Growth phase^b^	Growth condition^c^	Description	Number of replicates
1 (yellow)	*cckA*	ML	YPS 37°C	SB1003 *cckA* mutant	1
2 (yellow)	SBRM1	ML	YPS 37°C	SB1003 *ctrA* mutant	3
3 (blue)	ALS1	T	YPS 37°C	*gtaI* quorum sensing mutant	1
4 (blue)	SB1003	T	YPS 37°C	Wild type	1
5 (blue)	ALS1	ML	YPS 37°C	*gtaI* quorum sensing mutant	1
6 (blue)	SB1003	ML	YPS 30°C	Wild type	1
7 (blue)	SB1003	ML	YPS 37°C	Wild type	7
8 (green)	ALS1	ES	RCV 30°C	*gtaI* quorum sensing mutant	1
9 (green)	SB1003	ES	RCV 30°C	Wild type	1
10 (green)	SLRK	ES	RCV 30°C	*gtaR* quorum sensing mutant	1
11 (green)	SLRK	ML	RCV 30°C	*gtaR* quorum sensing mutant	1
12 (green)	ALS1	ML	RCV 30°C	*gtaI* quorum sensing mutant	1
13 (green)	SB1003	ML	RCV 30°C	Wild type	1
14 (orange)	DE442	ML	YPS 37°C	GTA overproducer	1
15 (orange)	DE442	T	YPS 37°C	GTA overproducer	1
16 (orange)	DE442	ES	YPS 37°C	GTA overproducer	1
17 (orange)	DE442	LS	YPS 37°C	GTA overproducer	1
18 (red)	*cckA*	ES	YPS 37°C	SB1003 *cckA* mutant	1
19 (red)	SBRM1	ES	YPS 37°C	SB1003 *ctrA* mutant	3
20 (red)	SB1003	LS	YPS 37°C	Wild type	2
21 (red)	SB1003	ES	YPS 30°C	Wild type	1
22 (red)	ALS1	ES	YPS 37°C	*gtaI* quorum sensing mutant	1
23 (red)	SB1003	ES	YPS 37°C	Wild type	7

^a^Colours in parentheses correspond to the clusters highlighted in Figures 1 and 2.

^b^ML, mid-logarithmic growth phase; ES, early stationary growth phase; LS, late stationary growth phase; T, the transition point between the logarithmic and stationary phases.

^c^All cultures were grown under phototrophic conditions. YPS and RCV represent complex and defined media, respectively.

**Table 2:** ***R. capsulatus*** **strains used in this study**

***R. capsulatus***strain	Details	Reference
SB1003	Genome-sequenced strain	[3]
SBRM1	SB1003 with disrupted *ctrA*	[5]
*cckA*	SB1003 with disrupted *cckA*	[28]
ALS1	SB1003 with disrupted *gtaI*	[42]
SLKR	SB1003 with disrupted *gtaR*	[43] ^a^
DE442	RcGTA overproducer	[44, 45]; Providence uncertain

^a^Describes the mutation of *gtaR* in a different parental strain.

Probes were mapped using BLAST+ 2.2.24 to coding sequences in the *R. capsulatus* chromosome and plasmid (sequences were downloaded from NCBI on 24 January 2012). Only hits with an E-value of less than 0.001 were considered. Probes that mapped to multiple genes were discarded from further analysis. If two or more probes mapped to a single gene, the expression value for that gene was determined by averaging the signals across those probes. Expression values were log2-transformed before being processed further. Normalized and log2 transformed expression values were averaged across replicate chips to generate an averaged expression value for each gene per experimental condition. Robust z-scores were obtained and used to construct the co-expression network. The robust z-score is the number of median absolute deviations (MAD) away from the median [46].

To build a signed weighted co-expression network and identify modules (clusters) of co-expressed genes, we used the function blockwiseModules in the R WGCNA package. The co-expression network was constructed based on all pairwise biweight midcorrelation values raised to a power β equal to 18. Biweight midcorrelation is less susceptible to outliers than Pearson correlation [23]. We set the minimum module size to fifteen, reassignThreshold to zero, and pamRespectsDendro to false. All other WGCNA parameters remained at their default settings.

To determine if any underlying biological processes were enriched within the co-expression modules, we carried out over-representation analysis [47] using biological genes sets from KEGG [48] metabolic pathways, transcription units and protein complexes from MetaCyc [49], and protein domains from Pfam [50]. Functional annotations for all *R. capsulatus* genes were used for the over-representation analysis. The hypergeometric distribution was used to test for statistically significant over-representation of genes from particular biological gene sets within the co-expression modules. P-values were corrected for multiple testing using false discovery rate (FDR) [51]. Biological gene sets with an FDR-corrected p-value of less than 0.05 were deemed statistically significantly enriched within the given co-expression module. Full functional analysis results are provided in Additional file 5.

To investigate whether the genes in a co-expression module as a set were preferentially packaged or excluded from RcGTA particles, we used rank-based permutation tests and the microarray data from Hynes *et al.* [25]. Permutation tests (also called randomization tests) are non-parametric procedures for determining statistical significance based on rearrangements of the labels of a dataset. We performed a rank-based permutation approach where all genes were ranked based on the robust z-scores of their normalized and log2-transformed expression values from the DNA packaging array [25]. The observed ranks of the genes in a module were compared against the rank of 1000 randomly selected sets of genes of the same size (i.e., containing the same number of genes as the co-expressed module) using the Wilcoxon-Mann-Whitney test. Modules whose median rank were statistically lower (or greater) at a significance level of 0.01 than the median rank of 85% of the random gene sets (and no random gene set was statistically greater or lower) were considered to be differentially packed in RcGTA particles.

### *R. sphaeroides*mRNA expression data

We gathered all available *R. sphaeroides* mRNA expression data in NCBI GEO [38] using the R package GEOquery (version 2.26.2) [52]. The 192 microarray experiments collected had previously been published elsewhere [6–8, 53–64]. Linear expression values were log2-transformed. If two or more probes mapped to a single gene, the expression value for that gene was determined by averaging the signals across those probes. Robust z-scores were obtained and used to calculate the correlation matrix with Biweight midcorrelation.

### *R. capsulatus*proteomics data

Protein abundance data was collected from [5] and our own data on growth in a complex medium with and without supplemented phosphate (PeptideAtlas database accession: PASS00523). *R. capsulatus* SB1003 was cultured photoheterotrophically in 165 mL capped flat bottles for 36 hours at 30°C in YPSm medium or YPSm supplemented with 9.6 mM KPO_4_ pH 6.8 [26]. Cells were harvested from approximately 40 mL culture by centrifugation (15,000 rcf). The Accurate Mass and Time (AMT) tag proteomics approach was used to generate label-free relative quantification measurements [65]. Briefly, proteins from phosphate enriched and regular cell cultures were extracted from whole cell, soluble and insoluble lysate fractions then digested according to established protocols [5]. A pooled sample of peptides generated from each lysate fraction was further fractionated using strong cation exchange SCX-HPLC according to established protocols. 148 collected fractions (½ from phosphate enriched and ½ from phosphate depleted cell cultures) were then analyzed using a linear ion trap mass spectrometer (Thermo Scientific, San Jose CA) coupled to a reverse phase HPLC separation. MS instrumentation operating and HPLC separation conditions have been described previously for tandem mass spectra generation [5]. Peptide sequence assignment to tandem mass spectra was performed using SEQUEST [66] and results further processed using MSGF [67] in order to assign spectral probabilities. Only peptides having a spectral probability of less than 1×10^−10^ and a length of at least six amino acids were retained for matching to peptide feature data generated using high resolution FT-MS instrumentation (LTQ-Orbitrap; Thermo Scientific, San Jose CA) as described previously [68]. Arbitrary abundance measurements for matched peptides were determined by integrating the area under each LC-FT-MS peak for a given peptide feature. Measurements from multiple peptides uniquely mapped to a single protein were averaged to obtain one measurement of abundance per protein. Protein abundance data were normalized using a central tendency approach [69]. Normalized abundance values were log2-transformed and converted to z-scores (the number of standard deviations away from the mean). Z-scores were averaged across replicate conditions to generate an averaged abundance value for each protein per experimental condition. Proteins with more than two missing values were removed. Z-scores were used to calculate the correlation matrix with Biweight midcorrelation.

### Module preservation

Module preservation and quality statistics were computed using the modulePreservation function (1000 permutations) implemented in the R package WGCNA [19]. Network module preservation statistics assess whether modules identified in the reference network remain connected in the test network (density), and whether node connections are similar between the reference and the test network (connectivity). These statistics are calculated without the need to define modules in the test dataset. *R. capsulatus* transcriptomics data was our reference dataset; *R. sphaeroides* transcriptomics data and *R. capsulatus* proteomics data were our two test datasets. The complete set of network-based statistics obtained is provided in Additional file 8. This same procedure was used to determine the reproducibility *R. capsulatus* modules in the absence of the DE442 data.

### LNS calculation

We calculated LNS as described by Guan *et al.* [20]. Correlation values were transformed using the inverse hyperbolic tangent (atanh) function (also called Fisher’s z transformation), and LNS of a pair of orthologs is the correlation between their matched correlation vectors. Let *W*^A^ *= [w*^A^_*ij*_*]* and *W*^B^ *= [w*^B^_*ij*_*]* denote *n x n* matrices of atanh-transformed correlations, where A and B denote the species and *n* is the number of orthologs between these species. A correlation vector ***w***^A^ of an ortholog gene *j* is the *j*-th row of *W*^A^ with *n* components (w^A^_j1_, w^A^_j2_, … w^A^_jn_). The LNS of two ortholog genes *j* and *j'* is defined as the correlation between the correlation vectors ***w***^A^*(j)* and ***w***^B^*(j').*

The null distribution of LNS scores was obtained by randomizing the ortholog mapping table while preserving the correlation matrix and thus the network structure. Note that randomization might also be performed by permuting the gene labels in the correlation matrix (equivalent to shuffling node labels in the network); in this case, the null distribution will differ from the one obtained here. However, we considered that the network topology and the connectivity pattern of each node in the network should be preserved during randomization; thus, we favoured randomizing the ortholog mapping table. We performed 100 random permutations of the ortholog-mapping table. To obtain the within-species LNS, we evenly divided the conditions available per species and calculated the LNS per gene using the two resulting data subsets. This random subsampling process was repeated 100 times for each species.

### Availability of supporting data

The datasets supporting the results of this article are included within the article and its additional files. Microarray data have been deposited in the NCBI Gene Expression Omnibus (database accessions: GSE18149, GSE33176, GSE41014 and GSE53636) and proteomics data have been deposited in PeptideAtlas (database accession: PASS00523).

## Electronic supplementary material

Additional file 1: **Gene dendrogram and module labels from resampled data sets.** Cluster stability analysis results. (PDF 993 KB)

Additional file 2: **Module median expression profile similarities.** Pearson correlation coefficients between the median expression profiles of the identified modules. (PDF 2 MB)

Additional file 3: **Module heatmaps.** Expression profile of genes in all 40 identified co-expression modules across all conditions and/or mutant strains. (PDF 524 KB)

Additional file 4: **Gene module assignment.** Module assignment and functional annotation for all *R. capsulatus* genes. (XLS 813 KB)

Additional file 5: **Functional analysis results.** List of gene sets found statistically significantly enriched in the co-expression modules. (XLS 57 KB)

Additional file 6: **Preservation statistics of** ***R. capsulatus*** **gene modules in** ***R. capsulatus*** **proteomics data.** Module preservation as a function of module quality and relationship between the two preservation statistics, Zsummary.qual and medianRank. (PDF 784 KB)

Additional file 7: **LNS scores.** LNS scores for ortholog’ pairs between *Rhodobacter* species, and for *R. capsulatus* mRNA-protein data. (XLS 618 KB)

Additional file 8: **Network statistics.** Complete set of network-based statistics per co-expression module. (XLS 138 KB)
