# Supplementary material for: Gene co-expression network analysis in Rhodobacter capsulatus and application to comparative expression analysis of Rhodobacter sphaeroides
Source: BMC Genomics. 2014 Aug 28;15(1):730. doi: 10.1186/1471-2164-15-730 (PMC4158056; doi:10.1186/1471-2164-15-730)
Supplement: Supplementary file 1 — Additional file 1: Gene dendrogram and module labels from resampled data sets. Cluster stability analysis results. (PDF 993 KB) [file 12864_2014_6415_MOESM1_ESM.pdf]

Additional file 1. Gene dendrogram and module labels from resampled data sets. Cluster robustness analysis results. Top: Hierarchical clustering dendogram of all genes on the arrays; branches of the dendogram correspond to modules identified by the blockwiseModules function in WGCNA package. Bottom: Gene module assignment indicated by colour. The first row indicates module assignments obtained from the full data, and the 50 rows beneath the first row indicate the module assignments obtained from networks based on resampled sets of samples. More robust modules appear in every resampling/row.

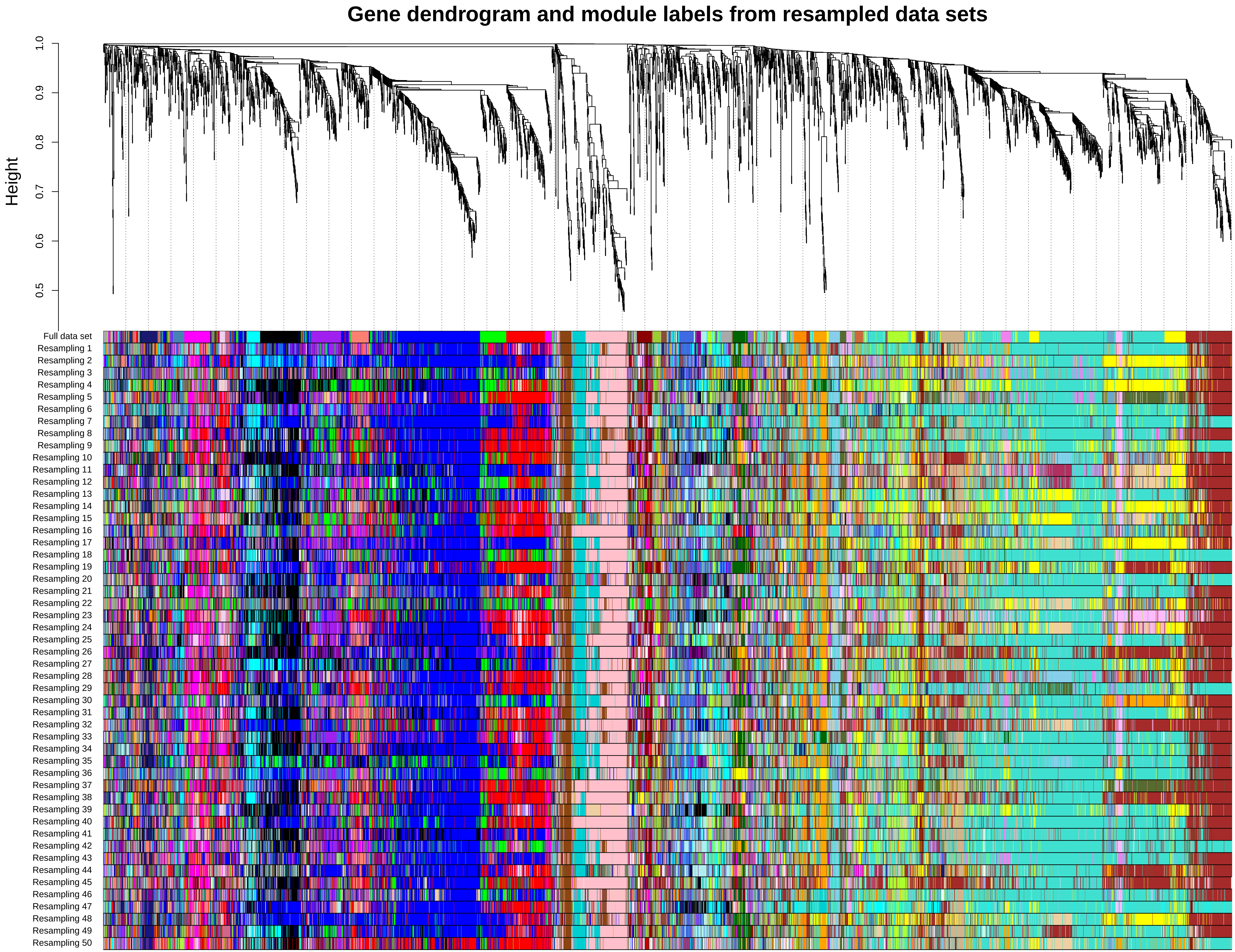
Gene dendrogram and module labels from resampled data sets
Height
1.0
0.9
0.8
0.7
0.6
0.5
Full data set
Resampling 1
Resampling 2
Resampling 3
Resampling 4
Resampling 5
Resampling 6
Resampling 7
Resampling 8
Resampling 9
Resampling 10
Resampling 11
Resampling 12
Resampling 13
Resampling 14
Resampling 15
Resampling 16
Resampling 17
Resampling 18
Resampling 19
Resampling 20
Resampling 21
Resampling 22
Resampling 23
Resampling 24
Resampling 25
Resampling 26
Resampling 27
Resampling 28
Resampling 29
Resampling 30
Resampling 31
Resampling 32
Resampling 33
Resampling 34
Resampling 35
Resampling 36
Resampling 37
Resampling 38
Resampling 39
Resampling 40
Resampling 41
Resampling 42
Resampling 43
Resampling 44
Resampling 45
Resampling 46
Resampling 47
Resampling 48
Resampling 49
Resampling 50
