# Supplementary material for: Gene co-expression network analysis in Rhodobacter capsulatus and application to comparative expression analysis of Rhodobacter sphaeroides
Source: BMC Genomics. 2014 Aug 28;15(1):730. doi: 10.1186/1471-2164-15-730 (PMC4158056; doi:10.1186/1471-2164-15-730)

Additional file 2. Module median expression profile similarities. Heatmap colours indicate Pearson correlation coefficients between the median expression profiles of the identified modules. The dendogram on the left shows the hierarchical clustering of modules based on similarity of median expression profiles between modules.

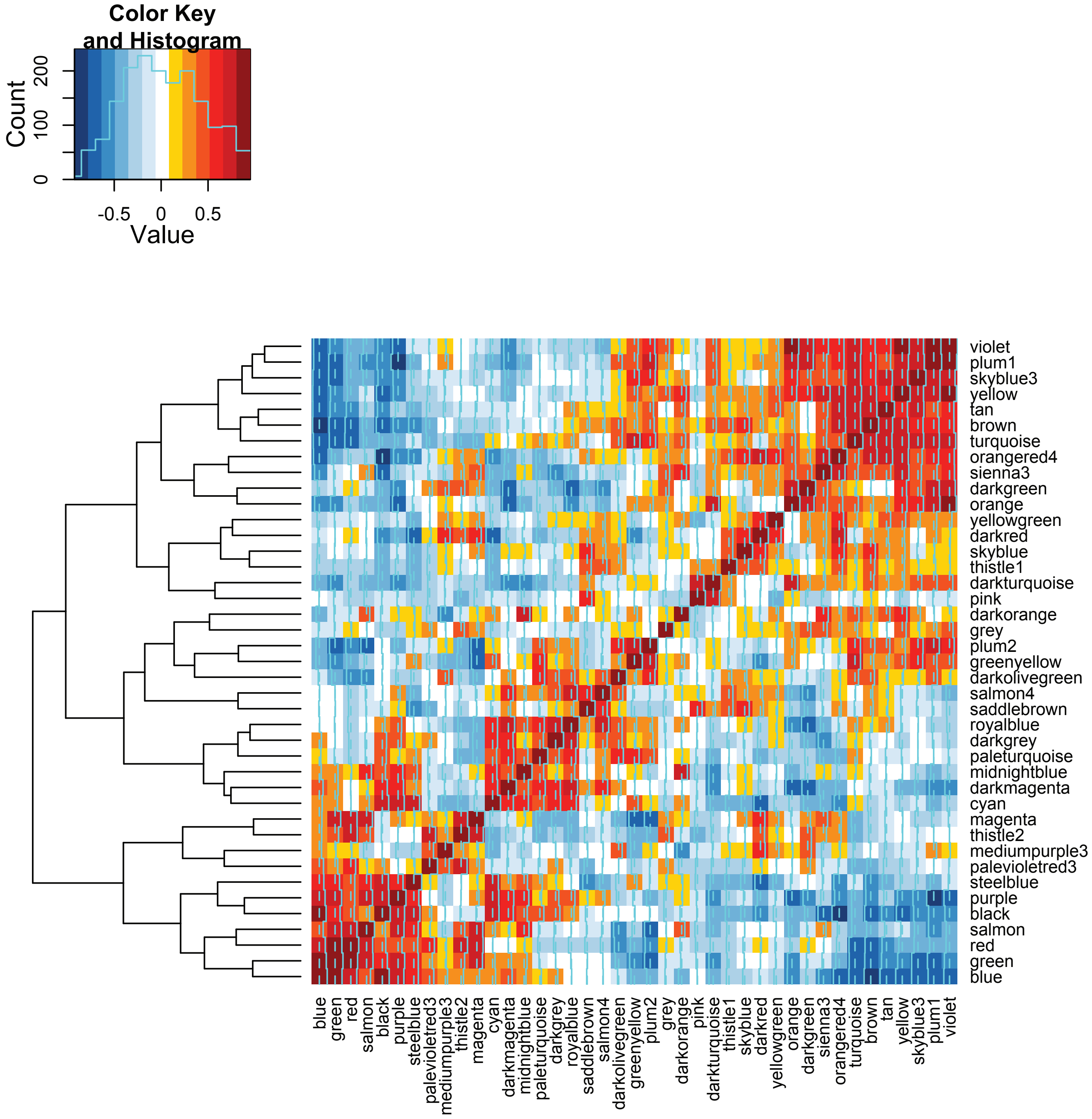

Supplement: Supplementary file 2 — Additional file 2: Module median expression profile similarities. Pearson correlation coefficients between the median expression profiles of the identified modules. (PDF 2 MB) [file 12864_2014_6415_MOESM2_ESM.pdf]
