# Supplementary material for: Gene co-expression network analysis in Rhodobacter capsulatus and application to comparative expression analysis of Rhodobacter sphaeroides
Source: BMC Genomics. 2014 Aug 28;15(1):730. doi: 10.1186/1471-2164-15-730 (PMC4158056; doi:10.1186/1471-2164-15-730)
Supplement: Supplementary file 3 — Additional file 3: Module heatmaps. Expression profile of genes in all 40 identified co-expression modules across all conditions and/or mutant strains. (PDF 524 KB) [file 12864_2014_6415_MOESM3_ESM.pdf]

Additional file 3. Module heatmaps. Expression profile of genes in all 40 identified co-expression modules across all conditions and/or mutant strains.

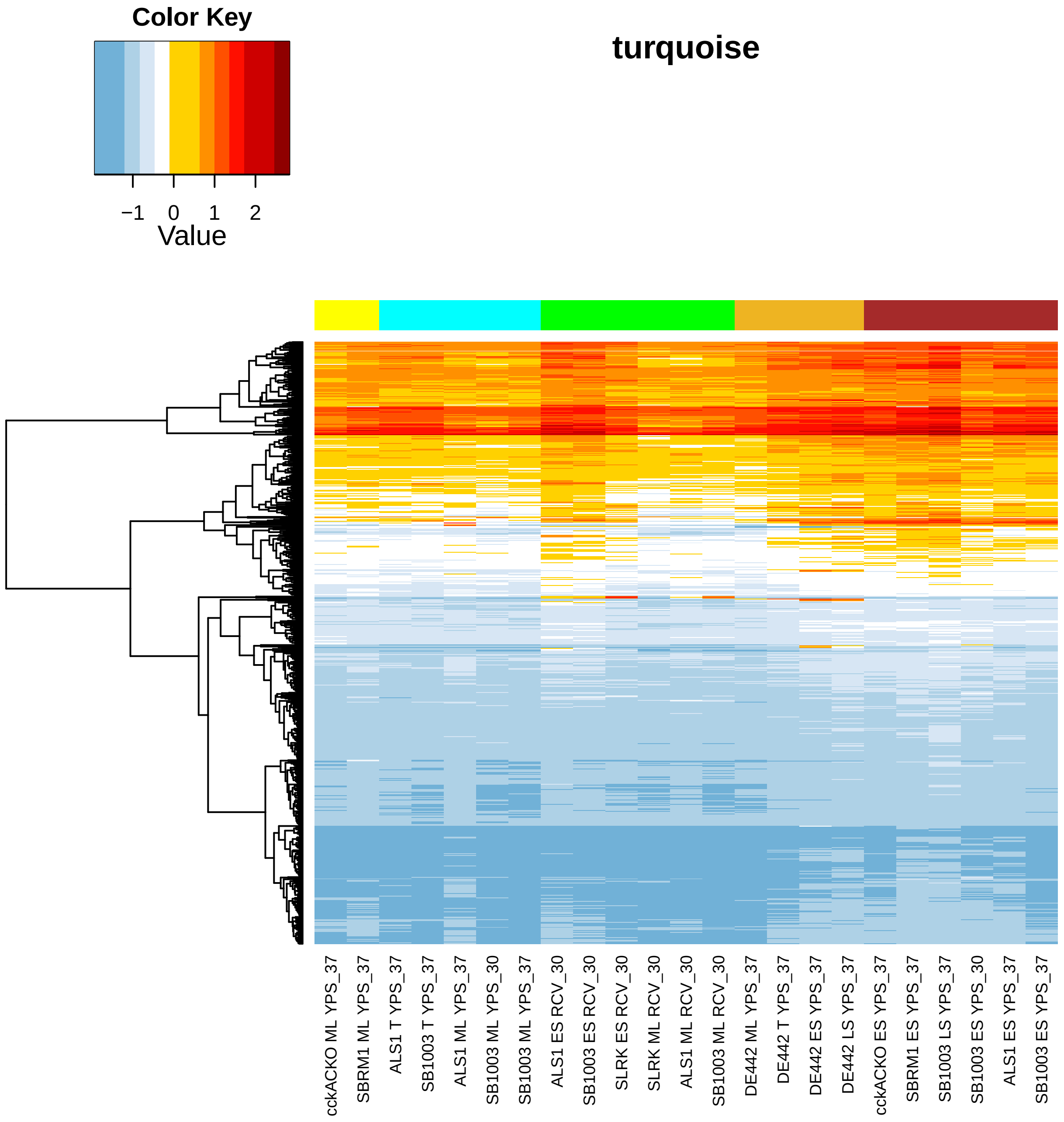

Color Key
−1
0
1
2
Value
turquoise
cckACKO ML YPS_37
SBRM1 ML YPS_37
ALS1 T YPS_37
SB1003 T YPS_37
ALS1 ML YPS_37
SB1003 ML YPS_30
SB1003 ML YPS_37
ALS1 ES RCV_30
SB1003 ES RCV_30
SLRK ES RCV_30
SLRK ML RCV_30
ALS1 ML RCV_30
SB1003 ML RCV_30
DE442 ML YPS_37
DE442 T YPS_37
DE442 ES YPS_37
DE442 LS YPS_37
cckACKO ES YPS_37
SBRM1 ES YPS_37
SB1003 LS YPS_37
SB1003 ES YPS_30
ALS1 ES YPS_37
SB1003 ES YPS_37

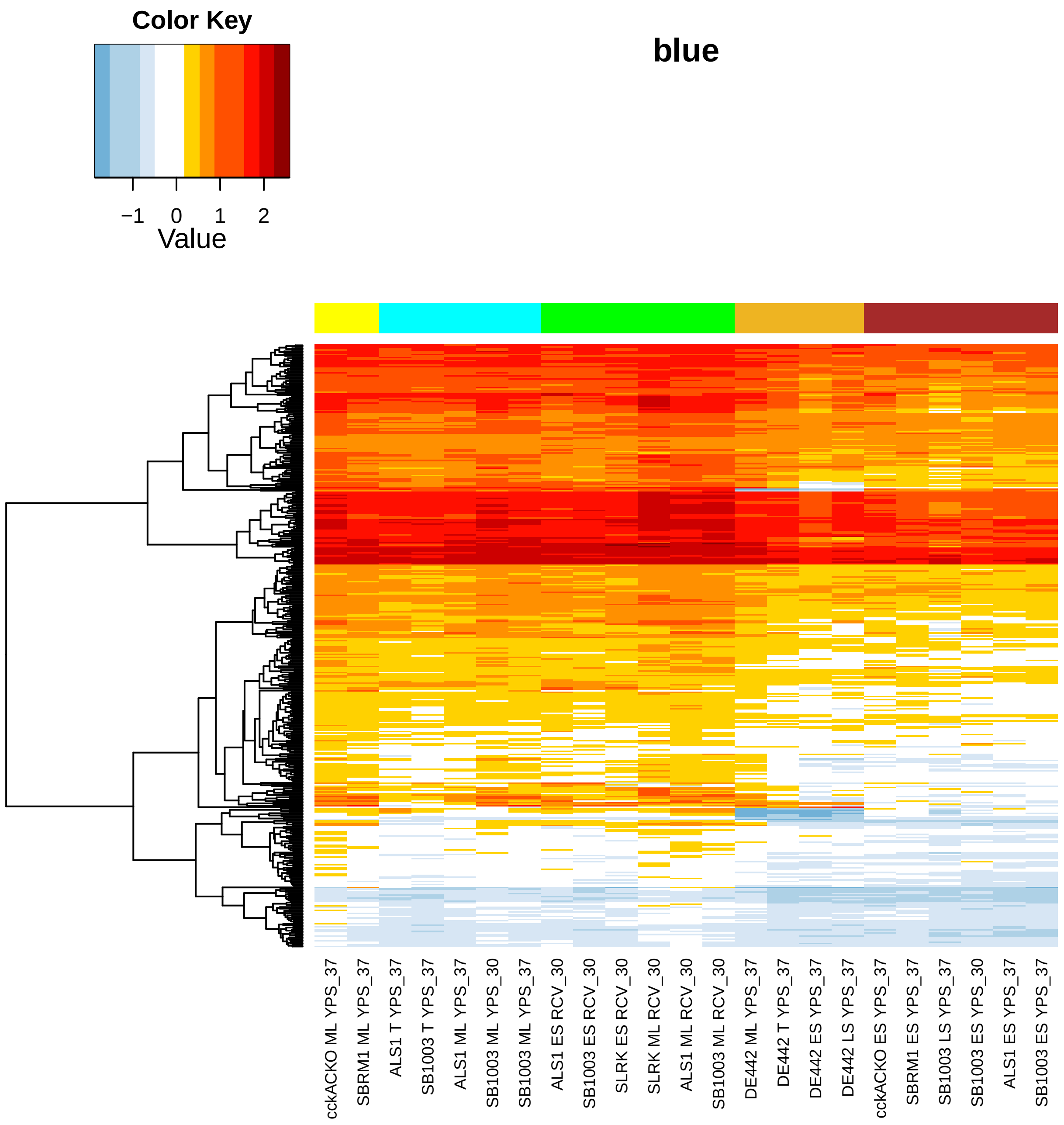
Color Key
−1
0
1
2
Value
blue
cckACKO ML YPS_37
SBRM1 ML YPS_37
ALS1 T YPS_37
SB1003 T YPS_37
ALS1 ML YPS_37
SB1003 ML YPS_30
SB1003 ML YPS_37
ALS1 ES RCV_30
SB1003 ES RCV_30
SLRK ES RCV_30
SLRK ML RCV_30
ALS1 ML RCV_30
SB1003 ML RCV_30
DE442 ML YPS_37
DE442 T YPS_37
DE442 ES YPS_37
DE442 LS YPS_37
cckACKO ES YPS_37
SBRM1 ES YPS_37
SB1003 LS YPS_37
SB1003 ES YPS_30
ALS1 ES YPS_37
SB1003 ES YPS_37

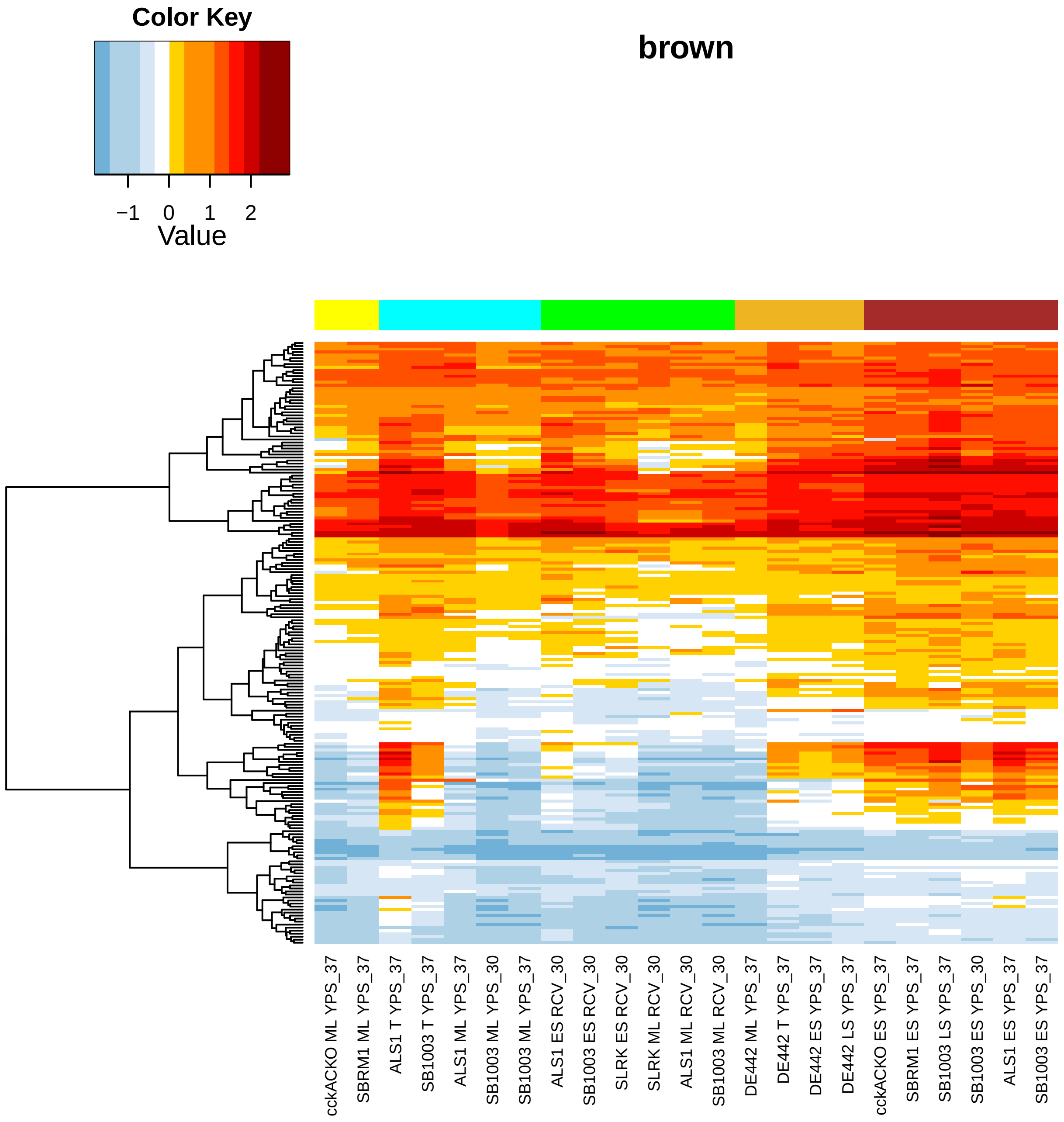

Color Key
−1
0
1
2
Value
brown
cckACKO ML YPS_37
SBRM1 ML YPS_37
ALS1 T YPS_37
SB1003 T YPS_37
ALS1 ML YPS_37
SB1003 ML YPS_30
SB1003 ML YPS_37
ALS1 ES RCV_30
SB1003 ES RCV_30
SLRK ES RCV_30
SLRK ML RCV_30
ALS1 ML RCV_30
SB1003 ML RCV_30
DE442 ML YPS_37
DE442 T YPS_37
DE442 ES YPS_37
DE442 LS YPS_37
cckACKO ES YPS_37
SBRM1 ES YPS_37
SB1003 LS YPS_37
SB1003 ES YPS_30
ALS1 ES YPS_37
SB1003 ES YPS_37

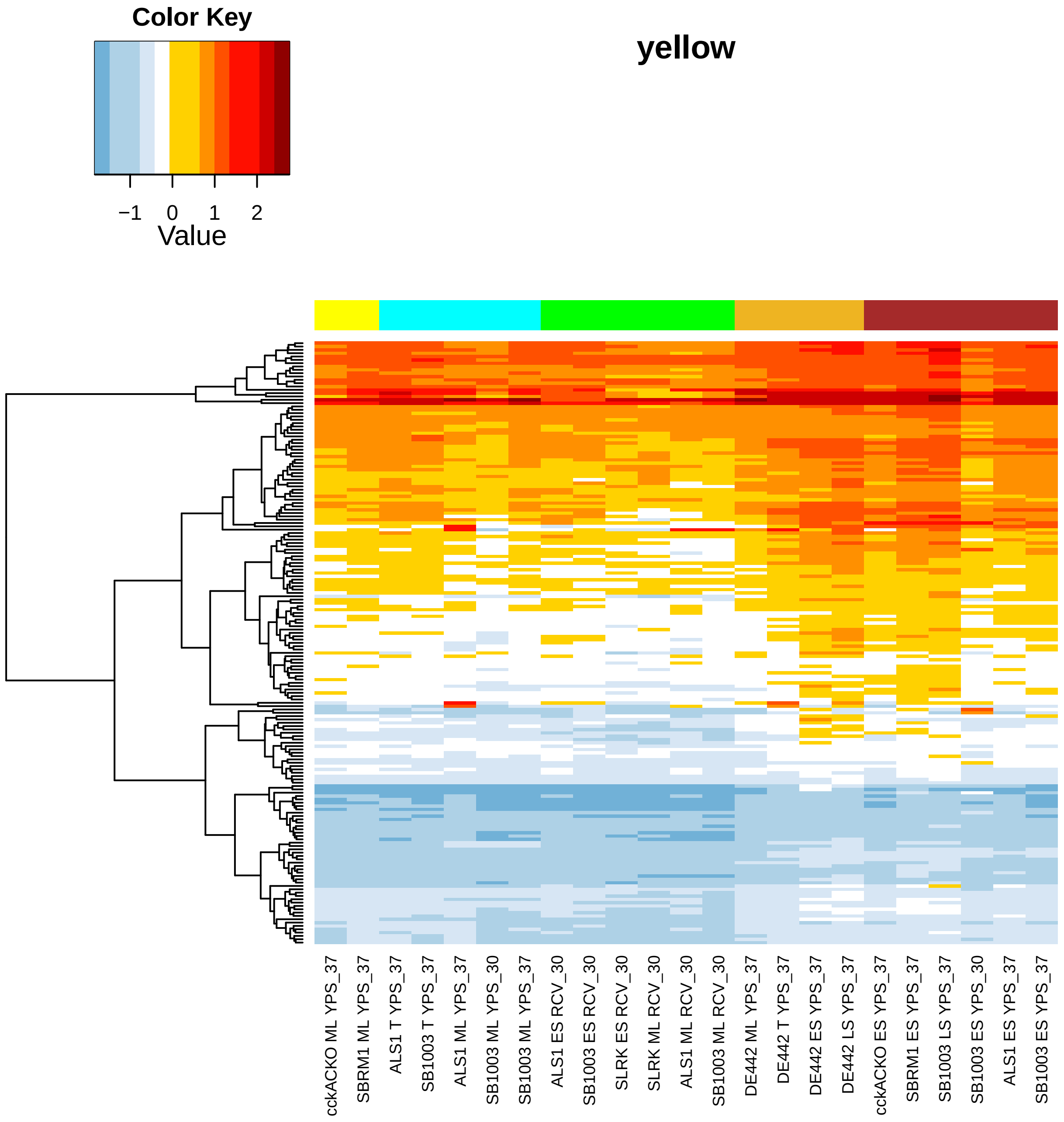

Color Key
−1 0 1 2
Value
yellow
cckACKO ML YPS_37
SBRM1 ML YPS_37
ALS1 T YPS_37
SB1003 T YPS_37
ALS1 ML YPS_37
SB1003 ML YPS_30
SB1003 ML YPS_37
ALS1 ES RCV_30
SB1003 ES RCV_30
SLRK ES RCV_30
SLRK ML RCV_30
ALS1 ML RCV_30
SB1003 ML RCV_30
DE442 ML YPS_37
DE442 T YPS_37
DE442 ES YPS_37
DE442 LS YPS_37
cckACKO ES YPS_37
SBRM1 ES YPS_37
SB1003 LS YPS_37
SB1003 ES YPS_30
ALS1 ES YPS_37
SB1003 ES YPS_37

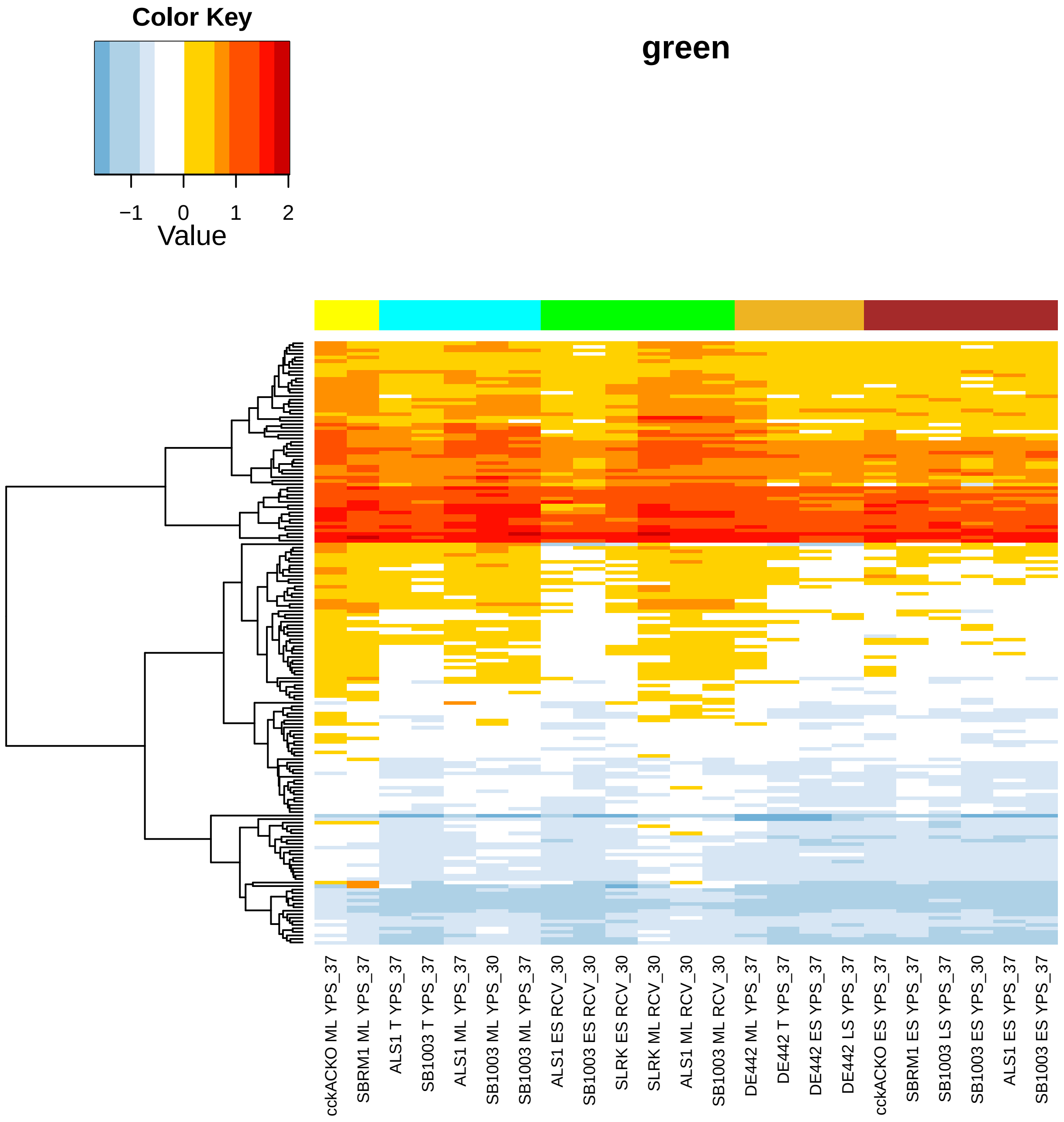
Color Key
−1
0
1
2
Value
green
cckACKO ML YPS_37
SBRM1 ML YPS_37
ALS1 T YPS_37
SB1003 T YPS_37
ALS1 ML YPS_37
SB1003 ML YPS_30
SB1003 ML YPS_37
ALS1 ES RCV_30
SB1003 ES RCV_30
SLRK ES RCV_30
SLRK ML RCV_30
ALS1 ML RCV_30
SB1003 ML RCV_30
DE442 ML YPS_37
DE442 T YPS_37
DE442 ES YPS_37
DE442 LS YPS_37
cckACKO ES YPS_37
SBRM1 ES YPS_37
SB1003 LS YPS_37
SB1003 ES YPS_30
ALS1 ES YPS_37
SB1003 ES YPS_37

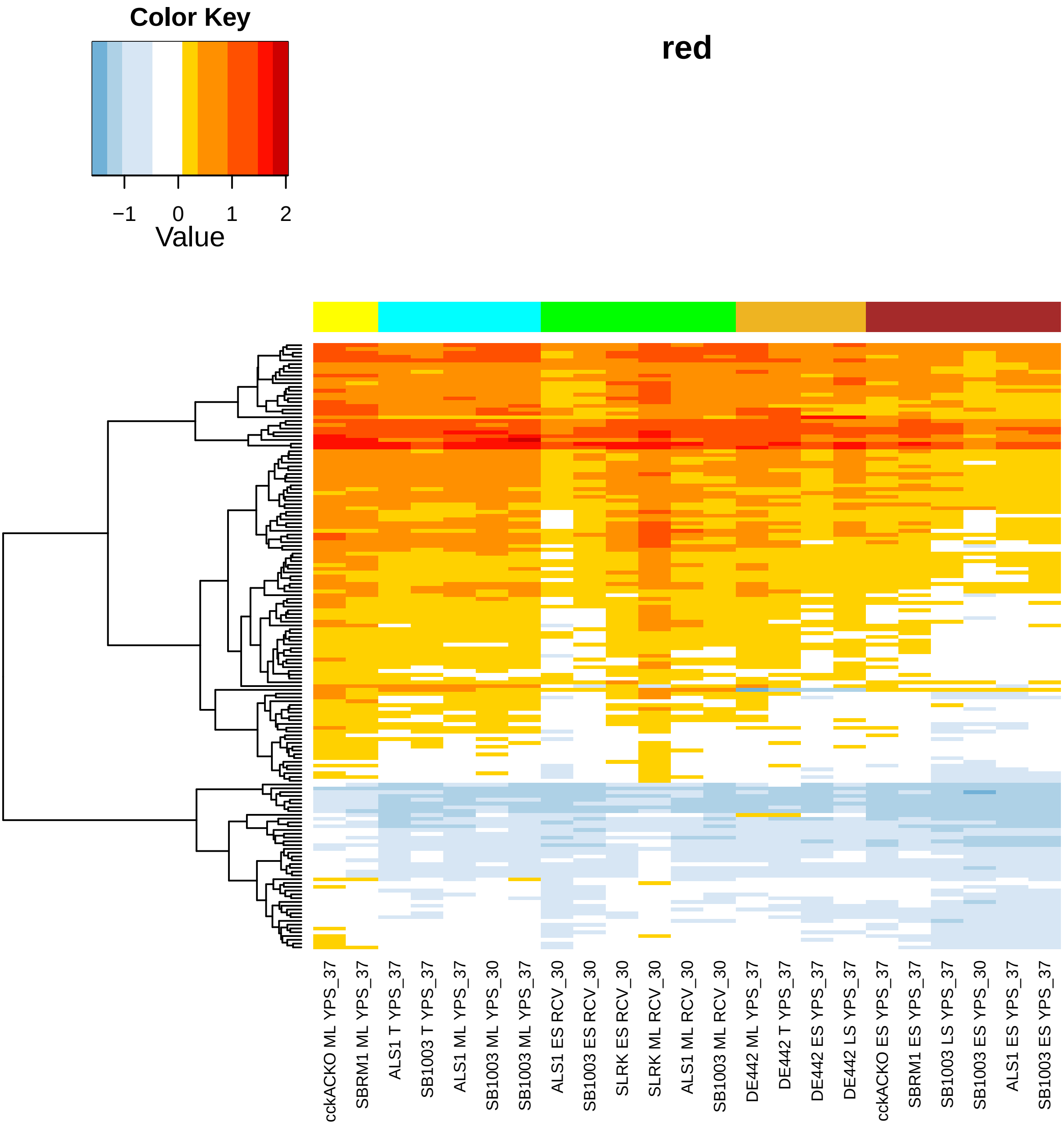
Color Key
−1
0
1
2
Value
red
cckACKO ML YPS_37
SBRM1 ML YPS_37
ALS1 T YPS_37
SB1003 T YPS_37
ALS1 ML YPS_37
SB1003 ML YPS_30
SB1003 ML YPS_37
ALS1 ES RCV_30
SB1003 ES RCV_30
SLRK ES RCV_30
SLRK ML RCV_30
ALS1 ML RCV_30
SB1003 ML RCV_30
DE442 ML YPS_37
DE442 T YPS_37
DE442 ES YPS_37
DE442 LS YPS_37
cckACKO ES YPS_37
SBRM1 ES YPS_37
SB1003 LS YPS_37
SB1003 ES YPS_30
ALS1 ES YPS_37
SB1003 ES YPS_37

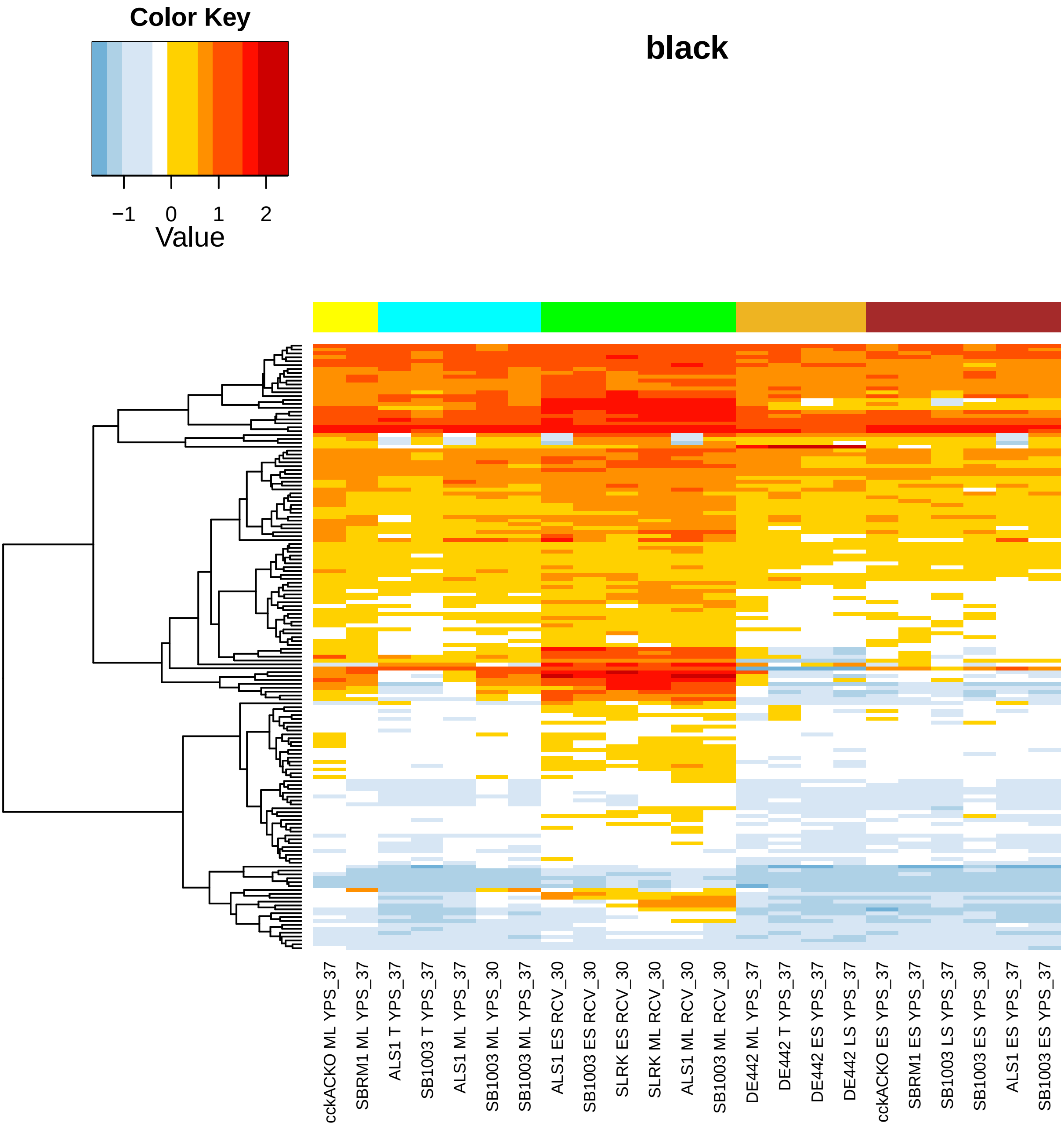
Color Key
−1 0 1 2
Value
black
cckACKO ML YPS_37
SBRM1 ML YPS_37
ALS1 T YPS_37
SB1003 T YPS_37
ALS1 ML YPS_37
SB1003 ML YPS_30
SB1003 ML YPS_37
ALS1 ES RCV_30
SB1003 ES RCV_30
SLRK ES RCV_30
SLRK ML RCV_30
ALS1 ML RCV_30
SB1003 ML RCV_30
DE442 ML YPS_37
DE442 T YPS_37
DE442 ES YPS_37
DE442 LS YPS_37
cckACKO ES YPS_37
SBRM1 ES YPS_37
SB1003 LS YPS_37
SB1003 ES YPS_30
ALS1 ES YPS_37
SB1003 ES YPS_37

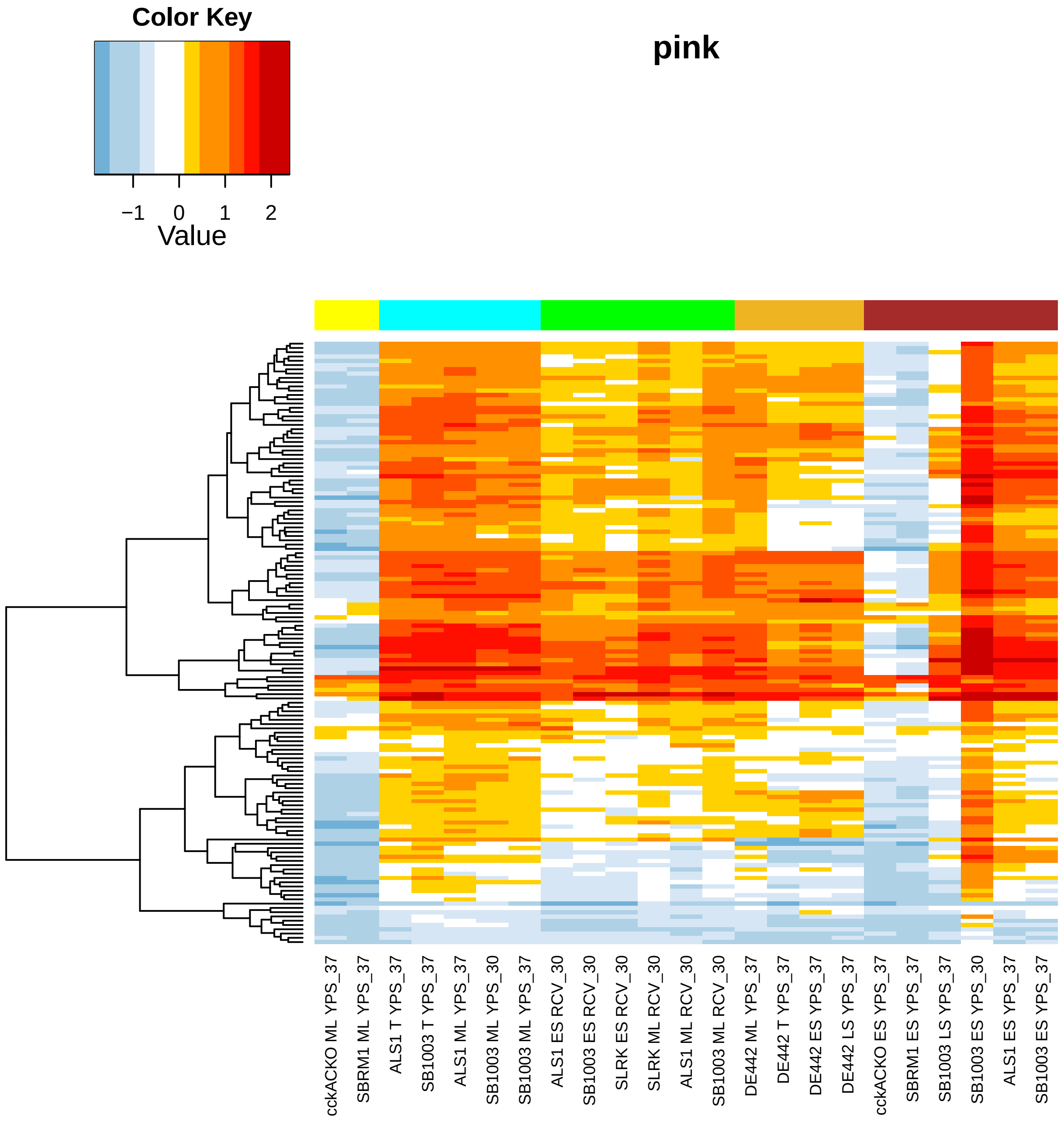

Color Key
−1
0
1
2
Value
pink
cckACKO ML YPS_37
SBRM1 ML YPS_37
ALS1 T YPS_37
SB1003 T YPS_37
ALS1 ML YPS_37
SB1003 ML YPS_30
SB1003 ML YPS_37
ALS1 ES RCV_30
SB1003 ES RCV_30
SLRK ES RCV_30
SLRK ML RCV_30
ALS1 ML RCV_30
SB1003 ML RCV_30
DE442 ML YPS_37
DE442 T YPS_37
DE442 ES YPS_37
DE442 LS YPS_37
cckACKO ES YPS_37
SBRM1 ES YPS_37
SB1003 LS YPS_37
SB1003 ES YPS_30
ALS1 ES YPS_37
SB1003 ES YPS_37

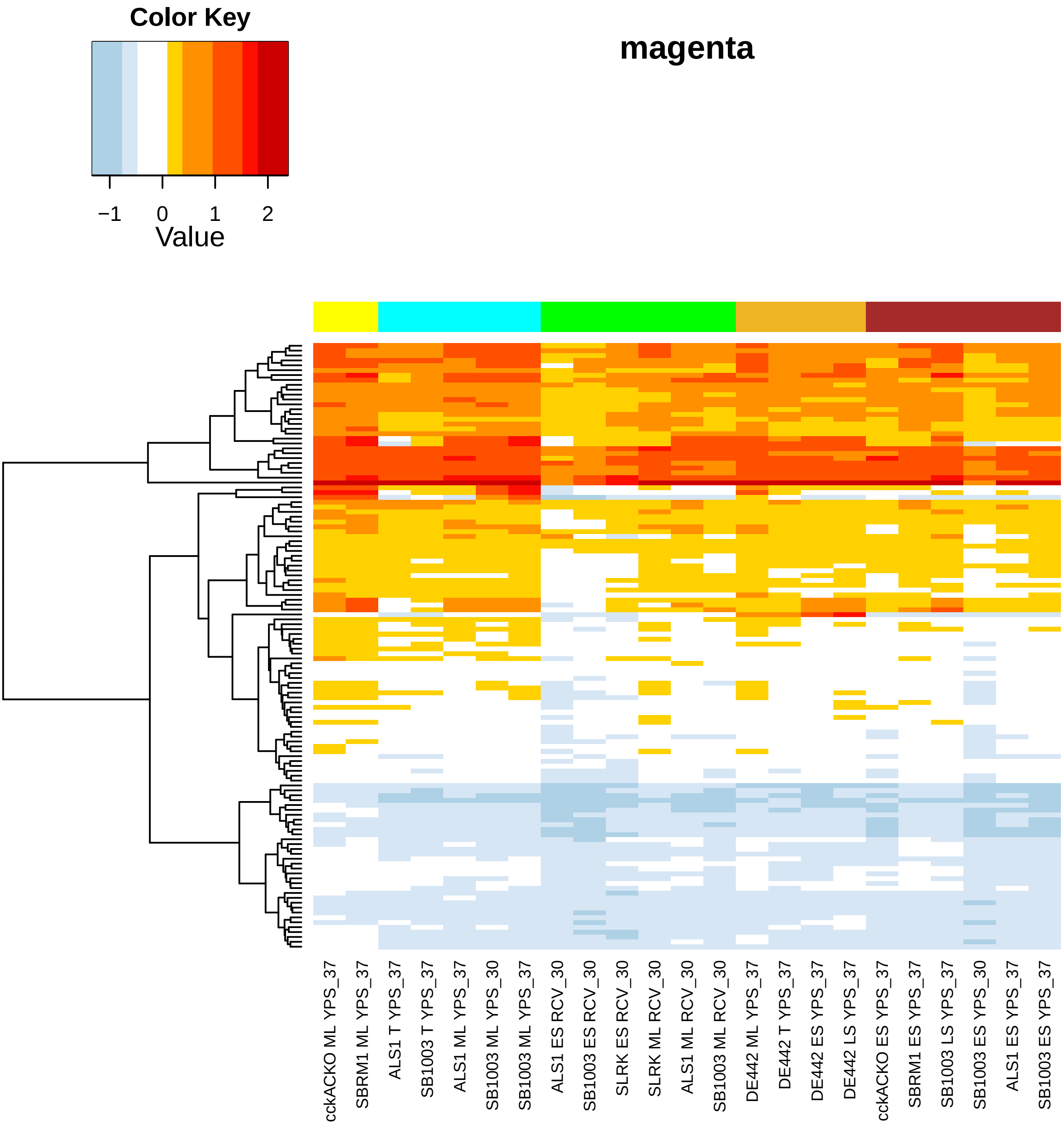
Color Key
−1
0
1
2
Value
magenta
ccKACKO ML YPS_37
SBRM1 ML YPS_37
ALS1 T YPS_37
SB1003 T YPS_37
ALS1 ML YPS_37
SB1003 ML YPS_30
SB1003 ML YPS_37
ALS1 ES RCV_30
SB1003 ES RCV_30
SLRK ES RCV_30
SLRK ML RCV_30
ALS1 ML RCV_30
SB1003 ML RCV_30
DE442 ML YPS_37
DE442 T YPS_37
DE442 ES YPS_37
DE442 LS YPS_37
ccKACKO ES YPS_37
SBRM1 ES YPS_37
SB1003 LS YPS_37
SB1003 ES YPS_30
ALS1 ES YPS_37
SB1003 ES YPS_37

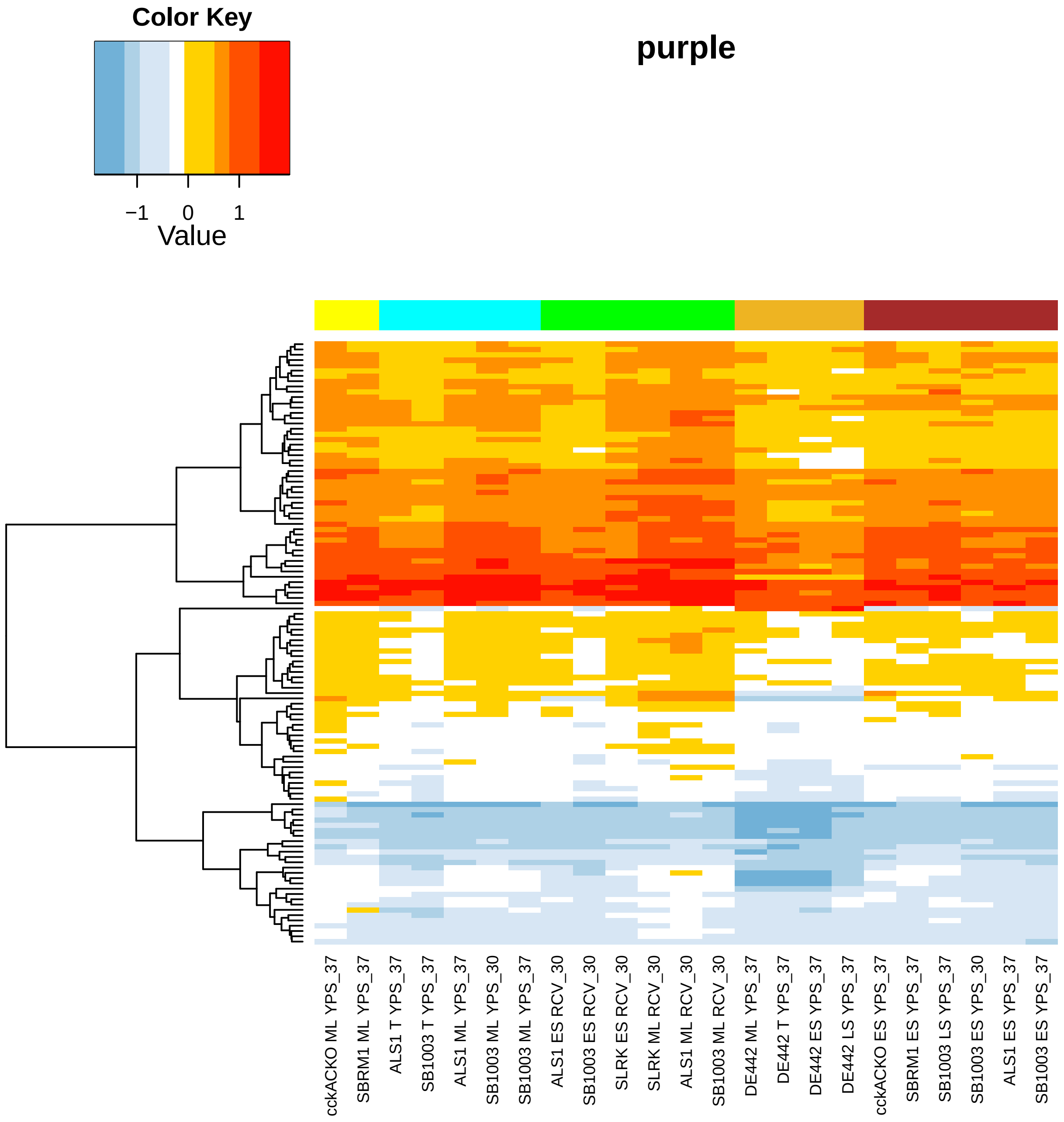

Color Key
−1
0
1
Value
purple
ccACKO ML YPS_37
SBRM1 ML YPS_37
ALS1 T YPS_37
SB1003 T YPS_37
ALS1 ML YPS_37
SB1003 ML YPS_30
SB1003 ML YPS_37
ALS1 ES RCV_30
SB1003 ES RCV_30
SLRK ES RCV_30
SLRK ML RCV_30
ALS1 ML RCV_30
SB1003 ML RCV_30
DE442 ML YPS_37
DE442 T YPS_37
DE442 ES YPS_37
DE442 LS YPS_37
ccACKO ES YPS_37
SBRM1 ES YPS_37
SB1003 LS YPS_37
SB1003 ES YPS_30
ALS1 ES YPS_37
SB1003 ES YPS_37

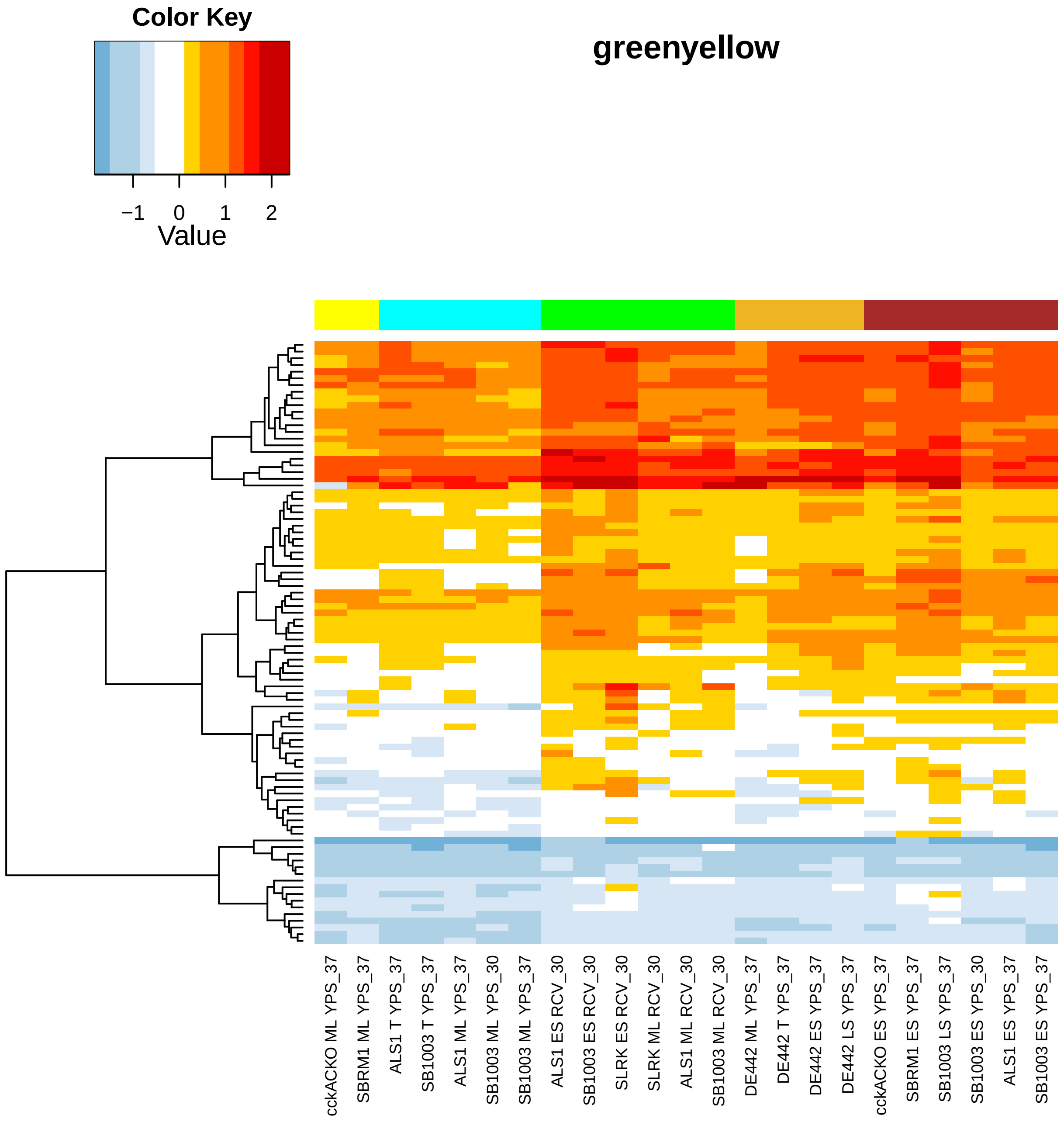
Color Key
−1
0
1
2
Value
greenyellow
cckACKO ML YPS_37
SBRM1 ML YPS_37
ALS1 T YPS_37
SB1003 T YPS_37
ALS1 ML YPS_37
SB1003 ML YPS_30
SB1003 ML YPS_37
ALS1 ES RCV_30
SB1003 ES RCV_30
SLRK ES RCV_30
SLRK ML RCV_30
ALS1 ML RCV_30
SB1003 ML RCV_30
DE442 ML YPS_37
DE442 T YPS_37
DE442 ES YPS_37
DE442 LS YPS_37
cckACKO ES YPS_37
SBRM1 ES YPS_37
SB1003 LS YPS_37
SB1003 ES YPS_30
ALS1 ES YPS_37
SB1003 ES YPS_37

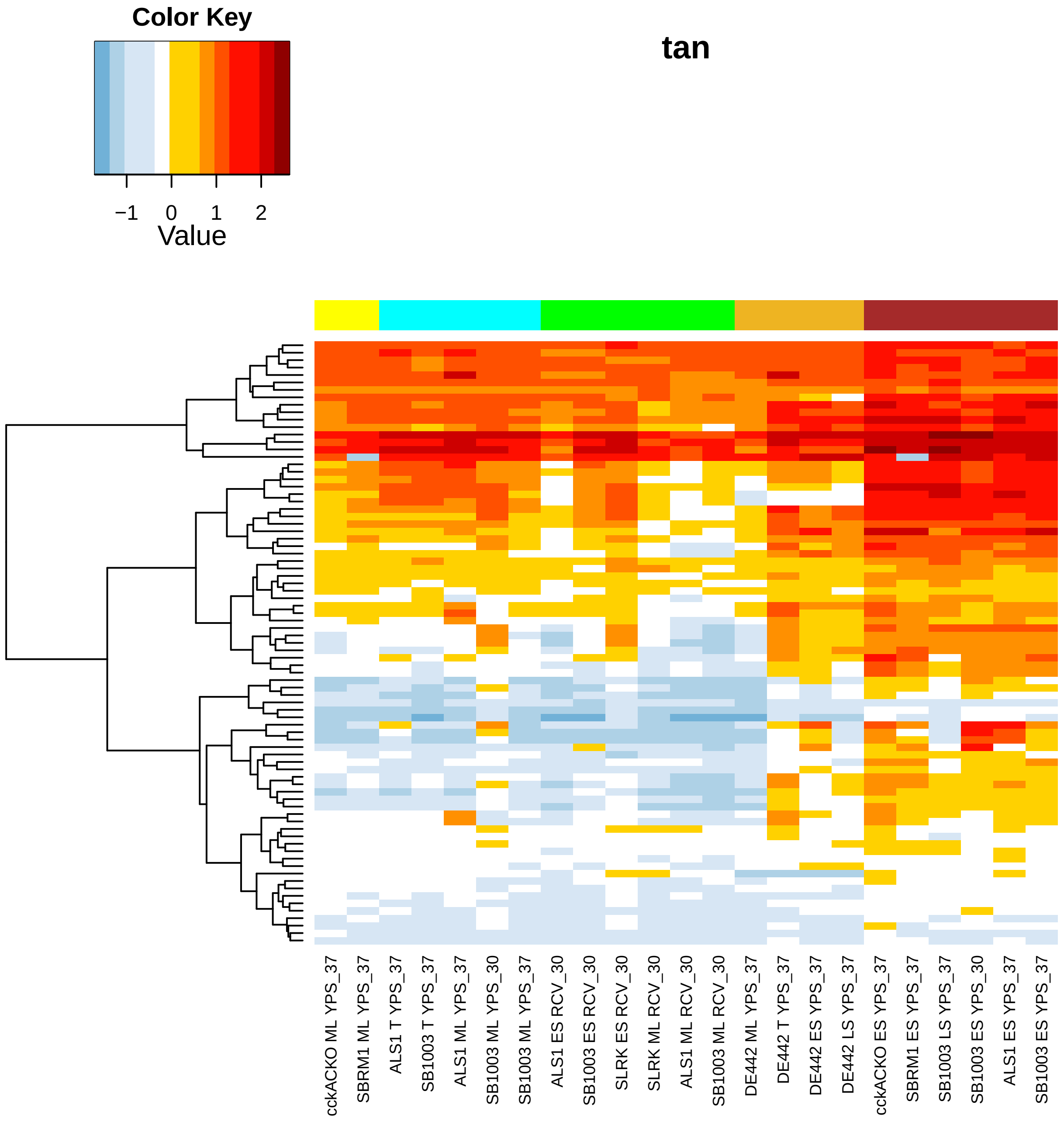
Color Key
−1
0
1
2
Value
tan
cckACKO ML YPS_37
SBRM1 ML YPS_37
ALS1 T YPS_37
SB1003 T YPS_37
ALS1 ML YPS_37
SB1003 ML YPS_30
SB1003 ML YPS_37
ALS1 ES RCV_30
SB1003 ES RCV_30
SLRK ES RCV_30
SLRK ML RCV_30
ALS1 ML RCV_30
SB1003 ML RCV_30
DE442 ML YPS_37
DE442 T YPS_37
DE442 ES YPS_37
DE442 LS YPS_37
cckACKO ES YPS_37
SBRM1 ES YPS_37
SB1003 LS YPS_37
SB1003 ES YPS_30
ALS1 ES YPS_37
SB1003 ES YPS_37

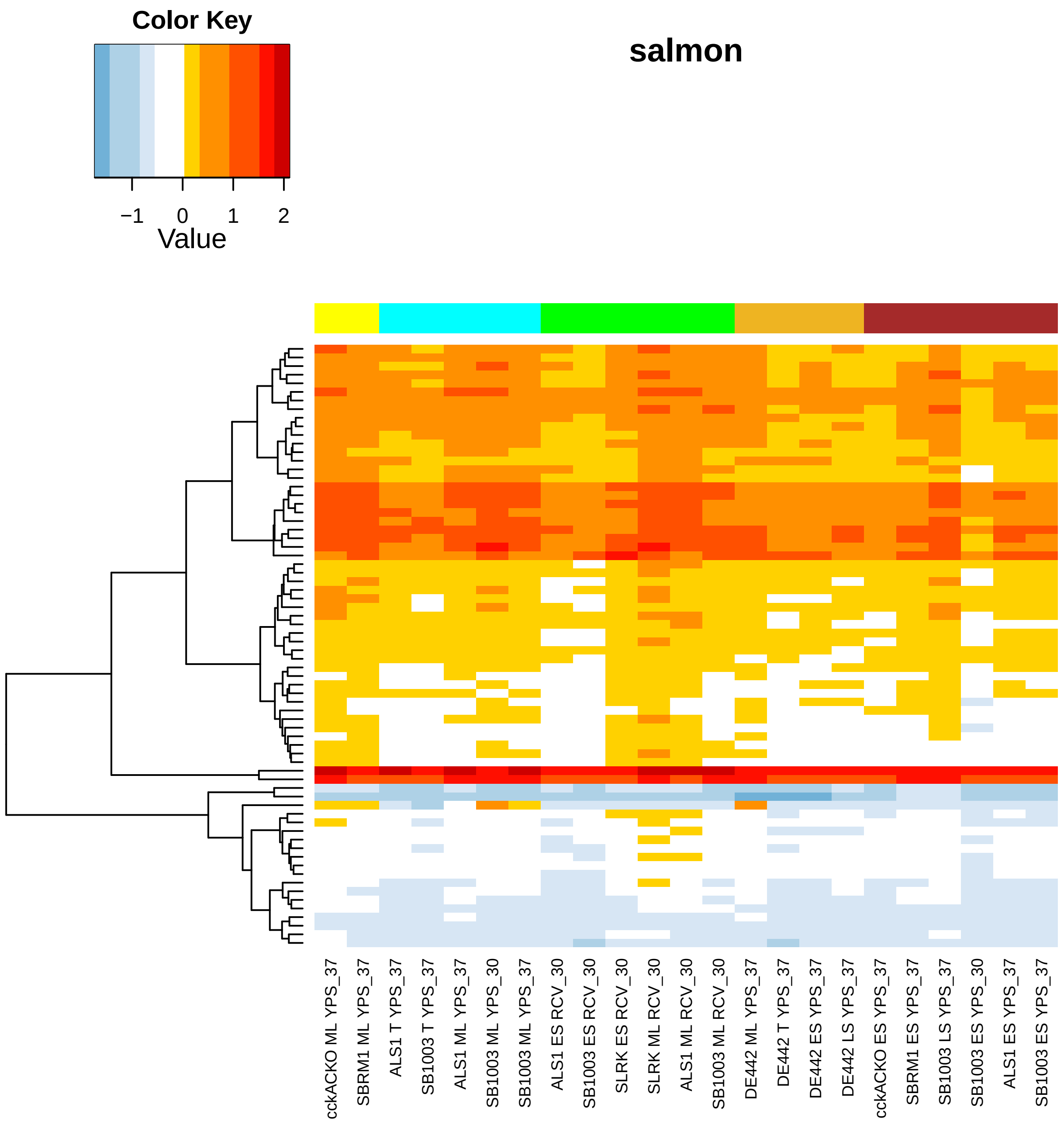

Color Key
−1
0
1
2
Value
salmon
cckACKO ML YPS_37
SBRM1 ML YPS_37
ALS1 T YPS_37
SB1003 T YPS_37
ALS1 ML YPS_37
SB1003 ML YPS_30
SB1003 ML YPS_37
ALS1 ES RCV_30
SB1003 ES RCV_30
SLRK ES RCV_30
SLRK ML RCV_30
ALS1 ML RCV_30
SB1003 ML RCV_30
DE442 ML YPS_37
DE442 T YPS_37
DE442 ES YPS_37
DE442 LS YPS_37
cckACKO ES YPS_37
SBRM1 ES YPS_37
SB1003 LS YPS_37
SB1003 ES YPS_30
ALS1 ES YPS_37
SB1003 ES YPS_37

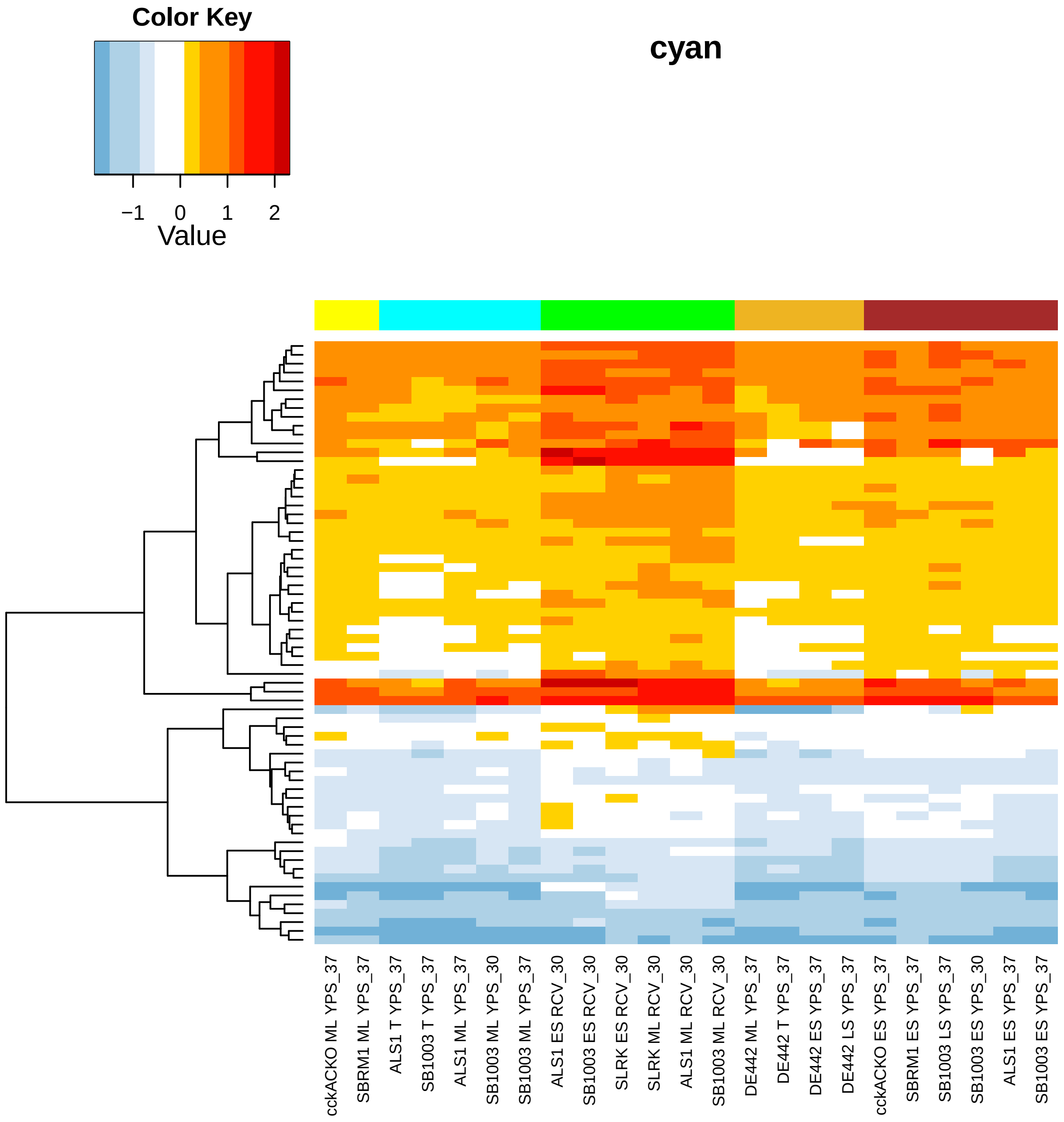

Color Key
−1
0
1
2
Value
cyan
cckACKO ML YPS_37
SBRM1 ML YPS_37
ALS1 T YPS_37
SB1003 T YPS_37
ALS1 ML YPS_37
SB1003 ML YPS_30
SB1003 ML YPS_37
ALS1 ES RCV_30
SB1003 ES RCV_30
SLRK ES RCV_30
SLRK ML RCV_30
ALS1 ML RCV_30
SB1003 ML RCV_30
DE442 ML YPS_37
DE442 T YPS_37
DE442 ES YPS_37
DE442 LS YPS_37
cckACKO ES YPS_37
SBRM1 ES YPS_37
SB1003 LS YPS_37
SB1003 ES YPS_30
ALS1 ES YPS_37
SB1003 ES YPS_37

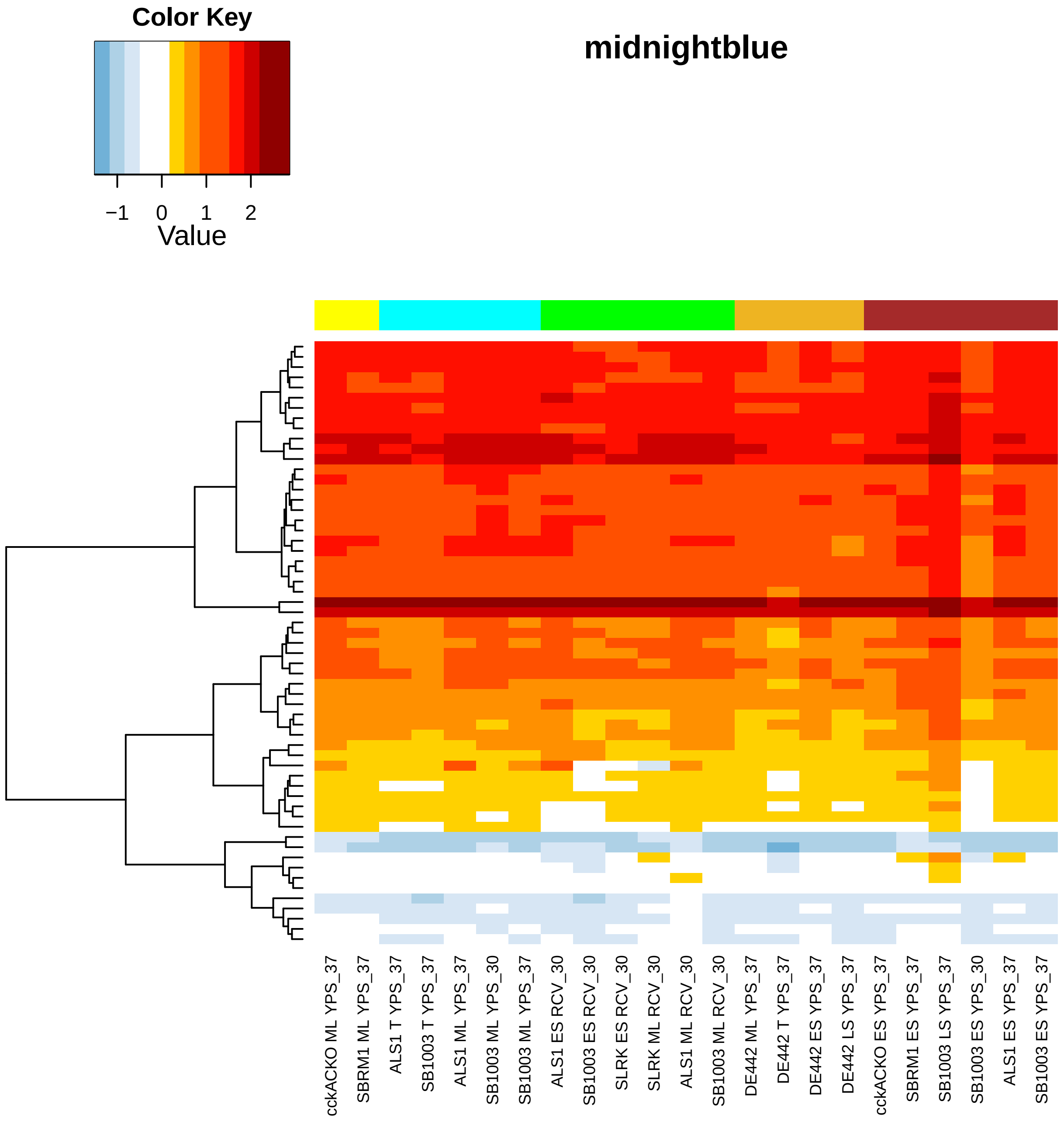

Color Key
−1
0
1
2
Value
midnightblue
cckACKO ML YPS_37
SBRM1 ML YPS_37
ALS1 T YPS_37
SB1003 T YPS_37
ALS1 ML YPS_37
SB1003 ML YPS_30
SB1003 ML YPS_37
ALS1 ES RCV_30
SB1003 ES RCV_30
SLRK ES RCV_30
SLRK ML RCV_30
ALS1 ML RCV_30
SB1003 ML RCV_30
DE442 ML YPS_37
DE442 T YPS_37
DE442 ES YPS_37
DE442 LS YPS_37
cckACKO ES YPS_37
SBRM1 ES YPS_37
SB1003 LS YPS_37
SB1003 ES YPS_30
ALS1 ES YPS_37
SB1003 ES YPS_37

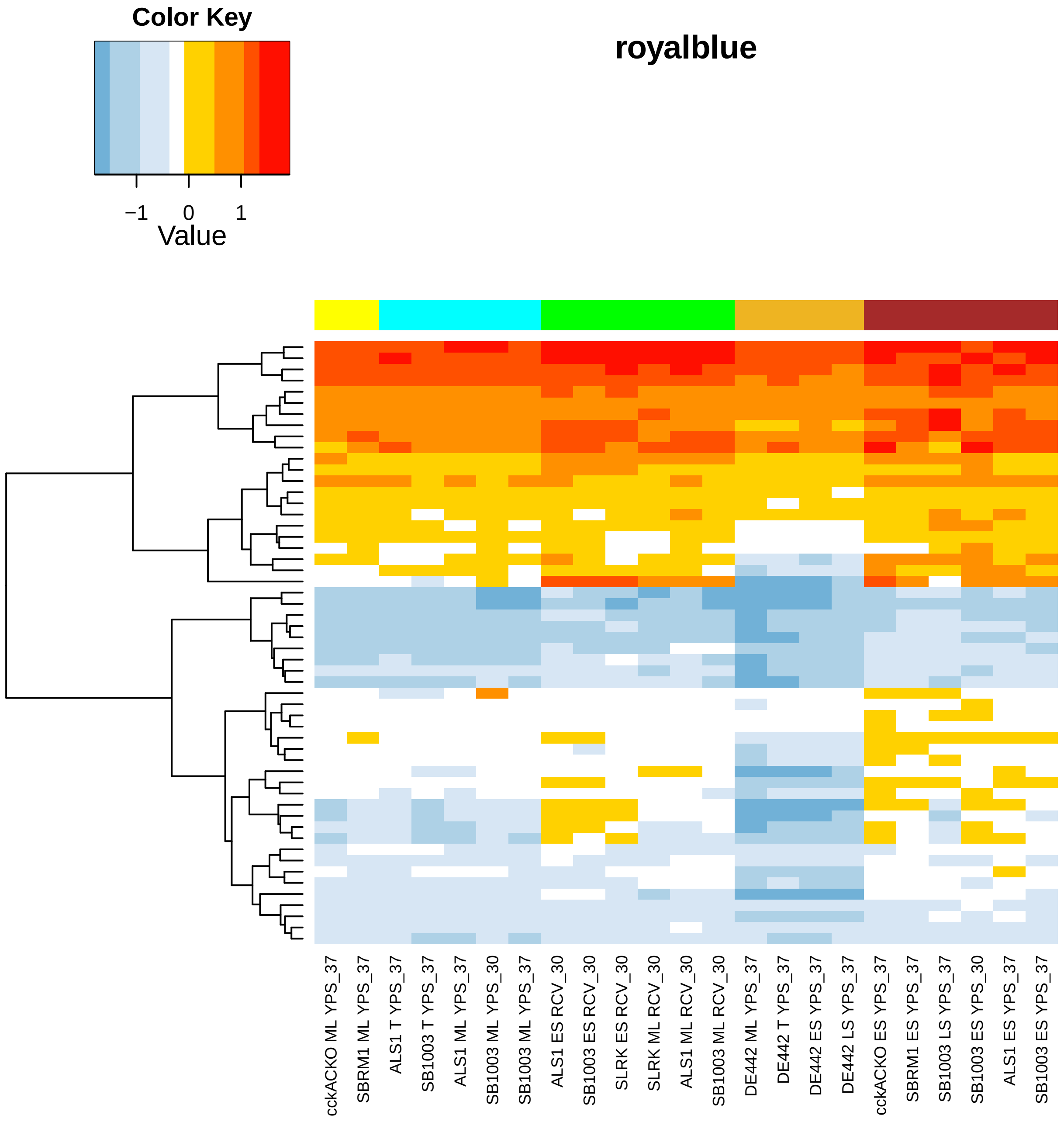

Color Key
−1
0
1
Value
royalblue
cckACKO ML YPS_37
SBRM1 ML YPS_37
ALS1 T YPS_37
SB1003 T YPS_37
ALS1 ML YPS_37
SB1003 ML YPS_30
SB1003 ML YPS_37
ALS1 ES RCV_30
SB1003 ES RCV_30
SLRK ES RCV_30
SLRK ML RCV_30
ALS1 ML RCV_30
SB1003 ML RCV_30
DE442 ML YPS_37
DE442 T YPS_37
DE442 ES YPS_37
DE442 LS YPS_37
cckACKO ES YPS_37
SBRM1 ES YPS_37
SB1003 LS YPS_37
SB1003 ES YPS_30
ALS1 ES YPS_37
SB1003 ES YPS_37

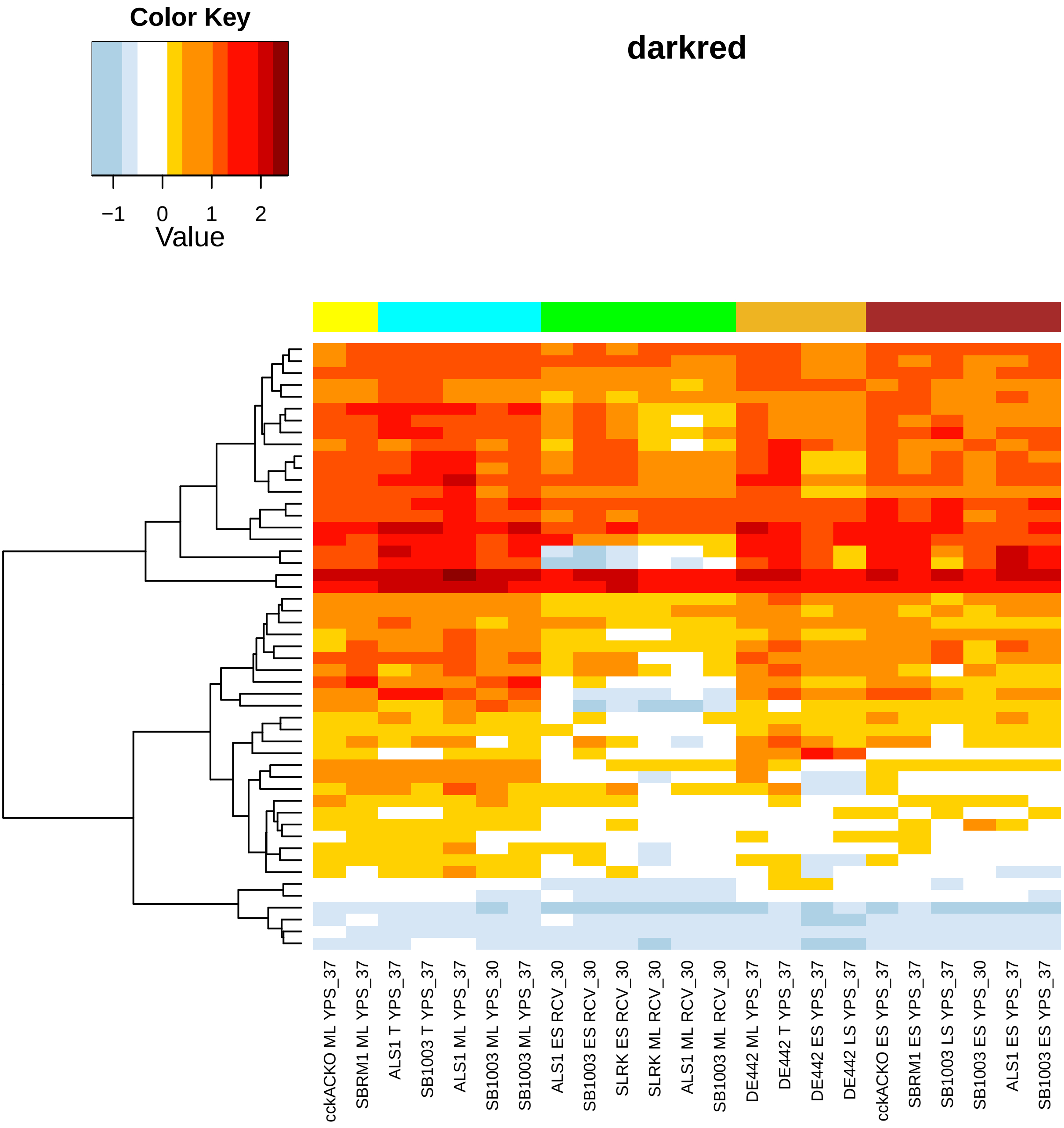

Color Key
−1
0
1
2
Value
darkred
cckACKO ML YPS_37
SBRM1 ML YPS_37
ALS1 T YPS_37
SB1003 T YPS_37
ALS1 ML YPS_37
SB1003 ML YPS_30
SB1003 ML YPS_37
ALS1 ES RCV_30
SB1003 ES RCV_30
SLRK ES RCV_30
SLRK ML RCV_30
ALS1 ML RCV_30
SB1003 ML RCV_30
DE442 ML YPS_37
DE442 T YPS_37
DE442 ES YPS_37
DE442 LS YPS_37
cckACKO ES YPS_37
SBRM1 ES YPS_37
SB1003 LS YPS_37
SB1003 ES YPS_30
ALS1 ES YPS_37
SB1003 ES YPS_37

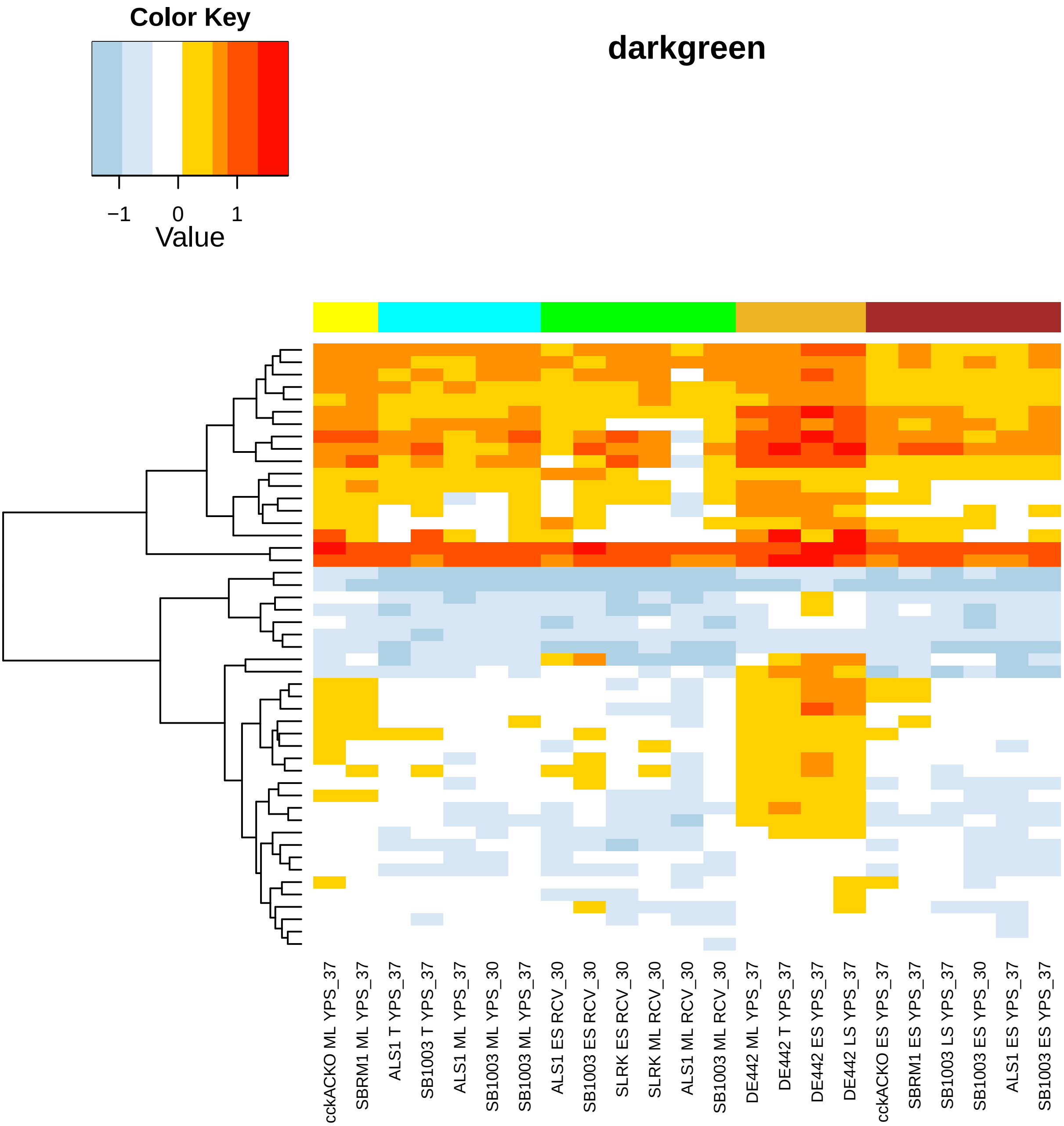

Color Key
−1
0
1
Value
darkgreen
cckACKO ML YPS_37
SBRM1 ML YPS_37
ALS1 T YPS_37
SB1003 T YPS_37
ALS1 ML YPS_37
SB1003 ML YPS_30
SB1003 ML YPS_37
ALS1 ES RCV_30
SB1003 ES RCV_30
SLRK ES RCV_30
SLRK ML RCV_30
ALS1 ML RCV_30
SB1003 ML RCV_30
DE442 ML YPS_37
DE442 T YPS_37
DE442 ES YPS_37
DE442 LS YPS_37
cckACKO ES YPS_37
SBRM1 ES YPS_37
SB1003 LS YPS_37
SB1003 ES YPS_30
ALS1 ES YPS_37
SB1003 ES YPS_37

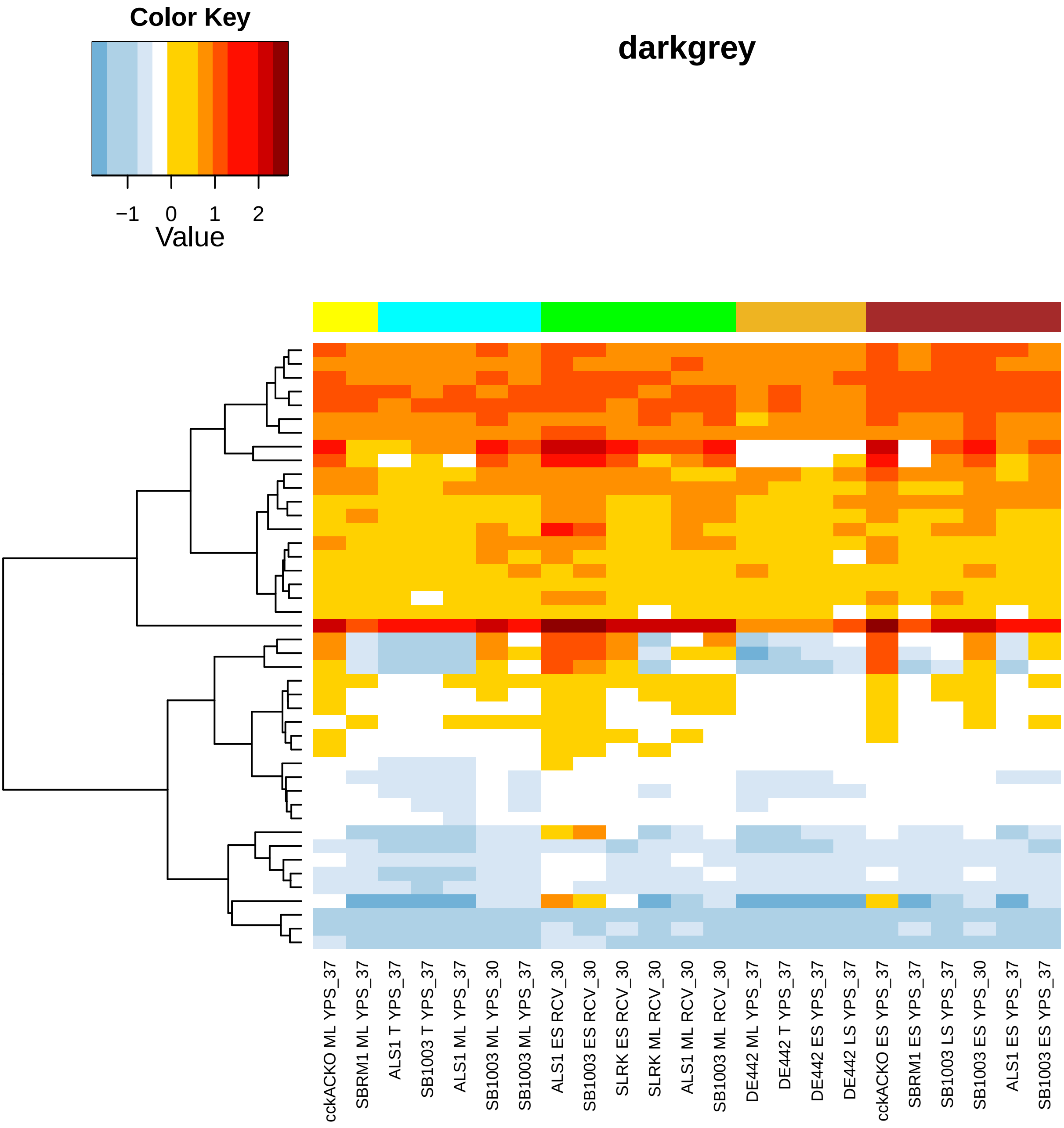

Color Key
−1
0
1
2
Value
darkgrey
cckACKO ML YPS_37
SBRM1 ML YPS_37
ALS1 T YPS_37
SB1003 T YPS_37
ALS1 ML YPS_37
SB1003 ML YPS_30
SB1003 ML YPS_37
ALS1 ES RCV_30
SB1003 ES RCV_30
SLRK ES RCV_30
SLRK ML RCV_30
ALS1 ML RCV_30
SB1003 ML RCV_30
DE442 ML YPS_37
DE442 T YPS_37
DE442 ES YPS_37
DE442 LS YPS_37
cckACKO ES YPS_37
SBRM1 ES YPS_37
SB1003 LS YPS_37
SB1003 ES YPS_30
ALS1 ES YPS_37
SB1003 ES YPS_37

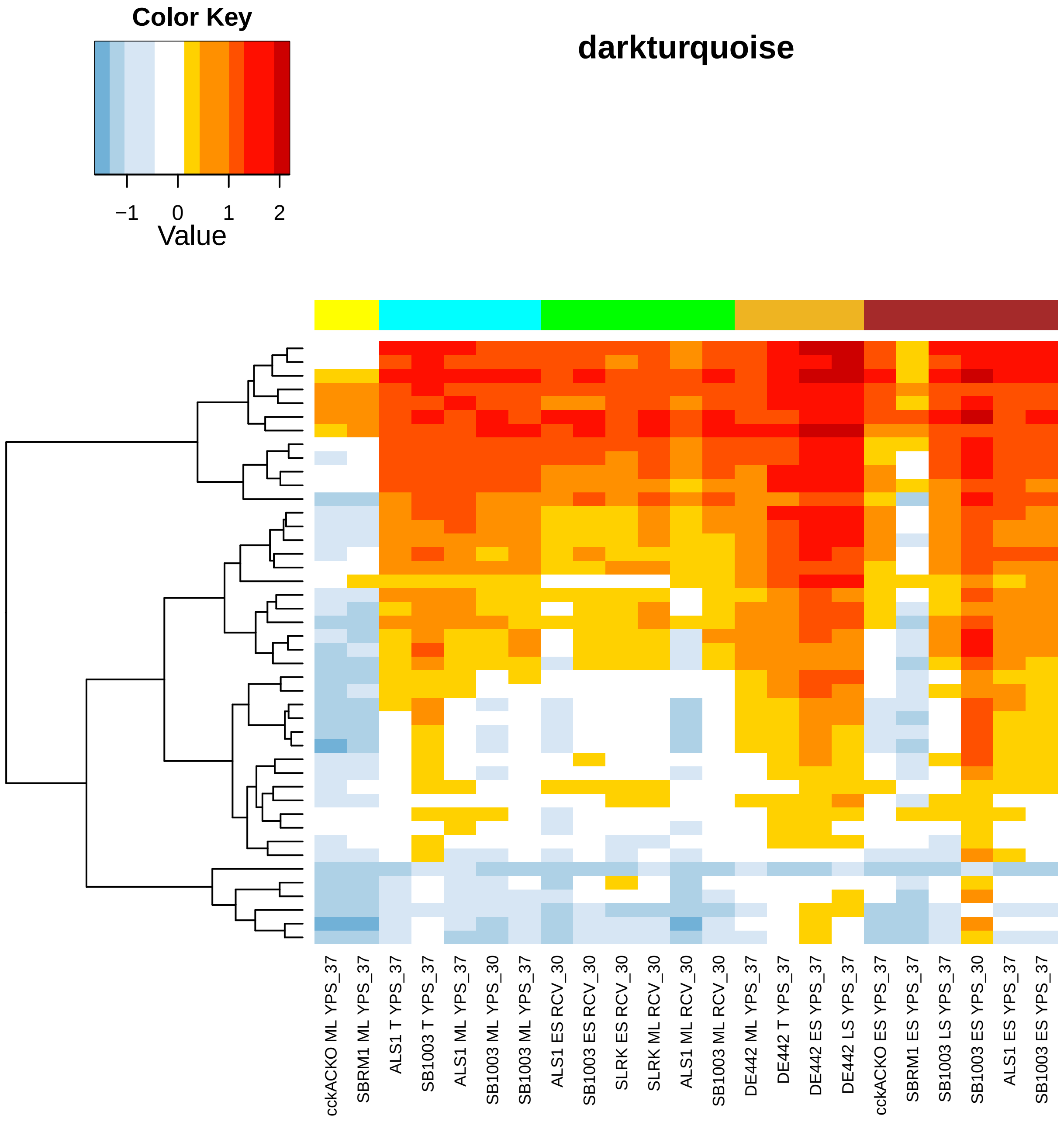

Color Key
−1
0
1
2
Value
darkturquoise
cckACKO ML YPS_37
SBRM1 ML YPS_37
ALS1 T YPS_37
SB1003 T YPS_37
ALS1 ML YPS_37
SB1003 ML YPS_30
SB1003 ML YPS_37
ALS1 ES RCV_30
SB1003 ES RCV_30
SLRK ES RCV_30
SLRK ML RCV_30
ALS1 ML RCV_30
SB1003 ML RCV_30
DE442 ML YPS_37
DE442 T YPS_37
DE442 ES YPS_37
DE442 LS YPS_37
cckACKO ES YPS_37
SBRM1 ES YPS_37
SB1003 LS YPS_37
SB1003 ES YPS_30
ALS1 ES YPS_37
SB1003 ES YPS_37

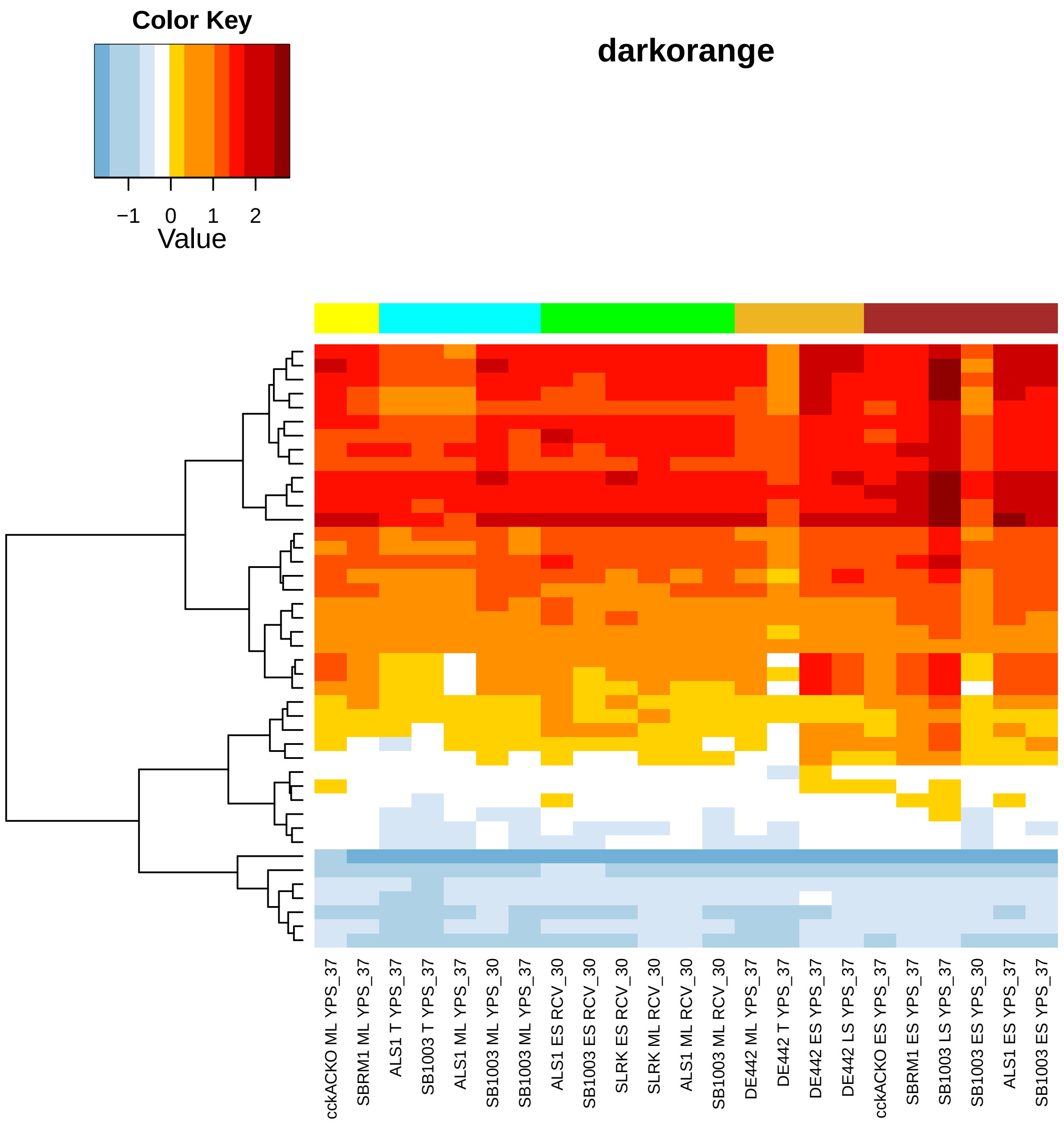

Color Key
−1
0
1
2
Value
darkorange
cckACKO ML YPS_37
SBRM1 ML YPS_37
ALS1 T YPS_37
SB1003 T YPS_37
ALS1 ML YPS_37
SB1003 ML YPS_30
SB1003 ML YPS_37
ALS1 ES RCV_30
SB1003 ES RCV_30
SLRK ES RCV_30
SLRK ML RCV_30
ALS1 ML RCV_30
SB1003 ML RCV_30
DE442 ML YPS_37
DE442 T YPS_37
DE442 ES YPS_37
DE442 LS YPS_37
cckACKO ES YPS_37
SBRM1 ES YPS_37
SB1003 LS YPS_37
SB1003 ES YPS_30
ALS1 ES YPS_37
SB1003 ES YPS_37

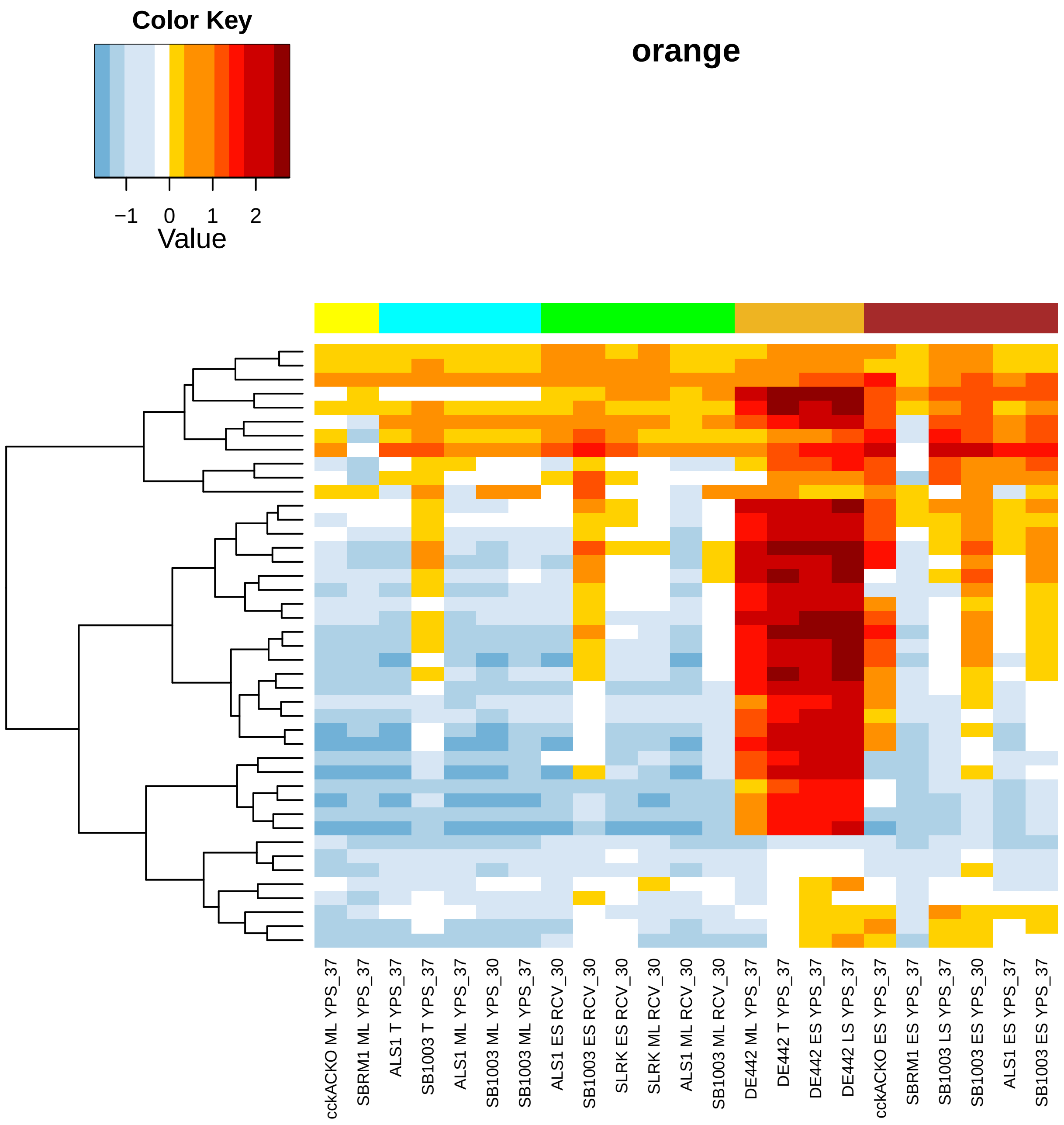

Color Key
−1
0
1
2
Value
orange
cckACKO ML YPS_37
SBRM1 ML YPS_37
ALS1 T YPS_37
SB1003 T YPS_37
ALS1 ML YPS_37
SB1003 ML YPS_30
SB1003 ML YPS_37
ALS1 ES RCV_30
SB1003 ES RCV_30
SLRK ES RCV_30
SLRK ML RCV_30
ALS1 ML RCV_30
SB1003 ML RCV_30
DE442 ML YPS_37
DE442 T YPS_37
DE442 ES YPS_37
DE442 LS YPS_37
cckACKO ES YPS_37
SBRM1 ES YPS_37
SB1003 LS YPS_37
SB1003 ES YPS_30
ALS1 ES YPS_37
SB1003 ES YPS_37

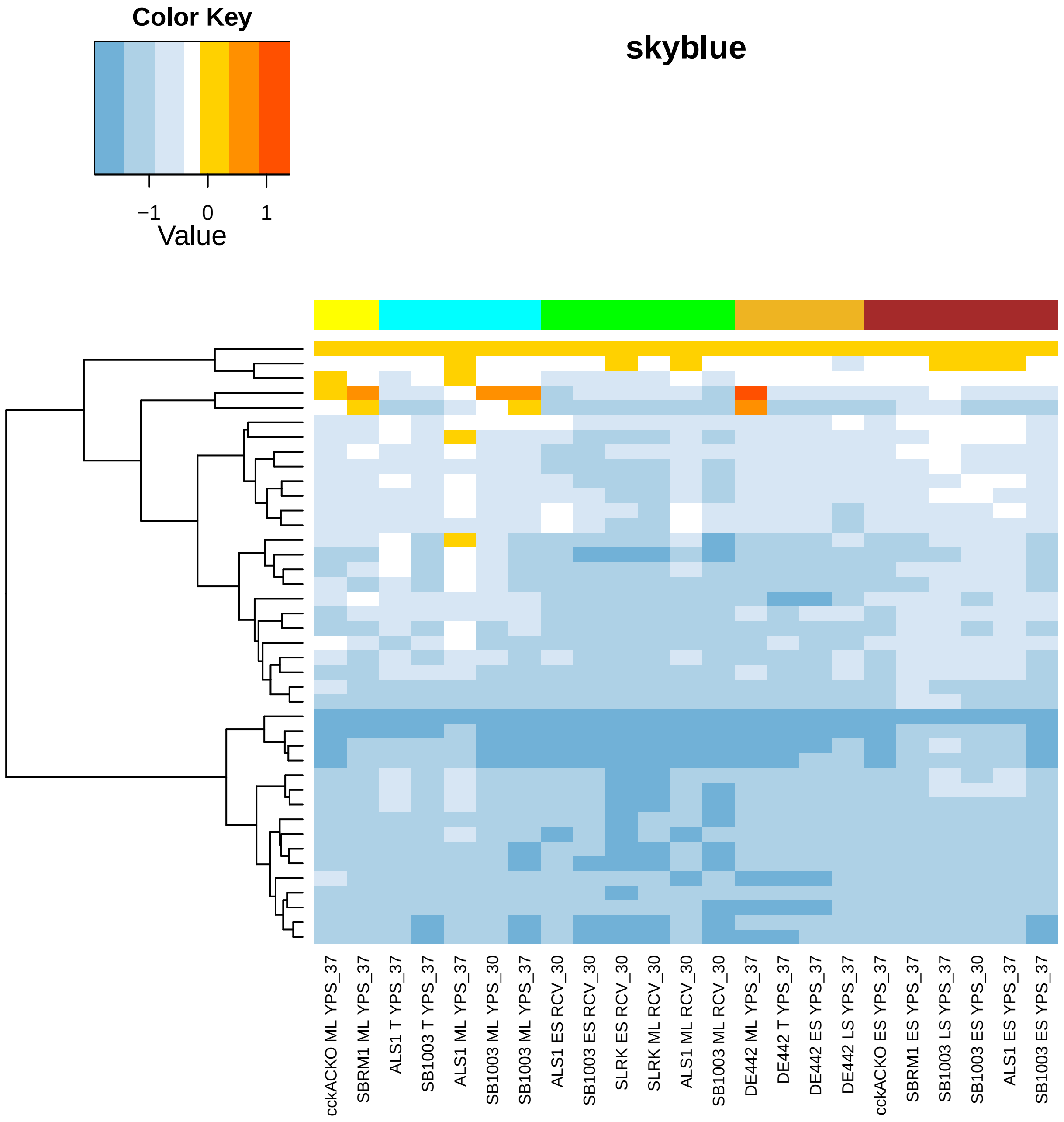

Color Key
−1
0
1
Value
skyblue
cckACKO ML YPS_37
SBRM1 ML YPS_37
ALS1 T YPS_37
SB1003 T YPS_37
ALS1 ML YPS_37
SB1003 ML YPS_30
SB1003 ML YPS_37
ALS1 ES RCV_30
SB1003 ES RCV_30
SLRK ES RCV_30
SLRK ML RCV_30
ALS1 ML RCV_30
SB1003 ML RCV_30
DE442 ML YPS_37
DE442 T YPS_37
DE442 ES YPS_37
DE442 LS YPS_37
cckACKO ES YPS_37
SBRM1 ES YPS_37
SB1003 LS YPS_37
SB1003 ES YPS_30
ALS1 ES YPS_37
SB1003 ES YPS_37

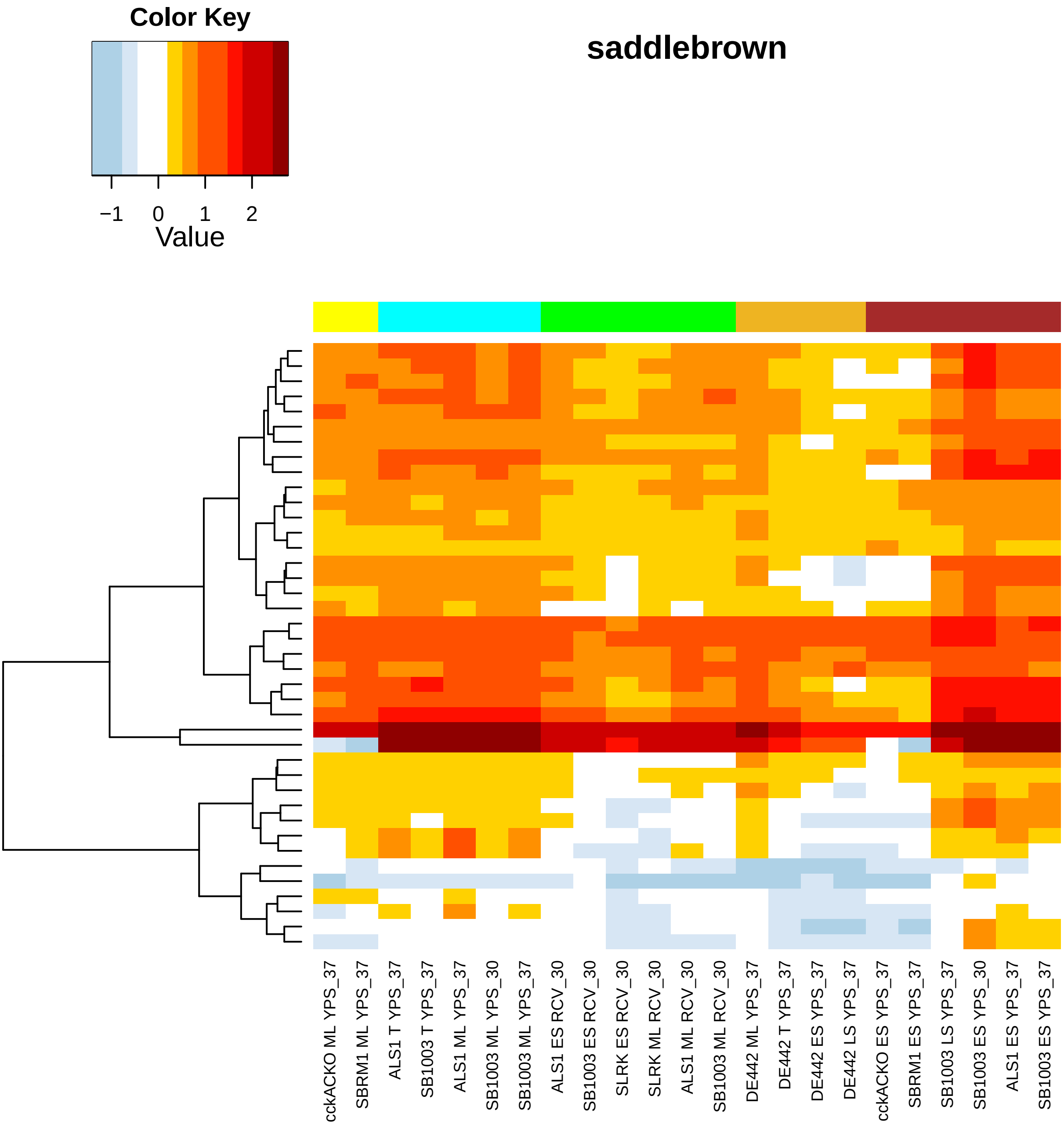
Color Key
−1
0
1
2
Value
saddlebrown
cckACKO ML YPS_37
SBRM1 ML YPS_37
ALS1 T YPS_37
SB1003 T YPS_37
ALS1 ML YPS_37
SB1003 ML YPS_30
SB1003 ML YPS_37
ALS1 ES RCV_30
SB1003 ES RCV_30
SLRK ES RCV_30
SLRK ML RCV_30
ALS1 ML RCV_30
SB1003 ML RCV_30
DE442 ML YPS_37
DE442 T YPS_37
DE442 ES YPS_37
DE442 LS YPS_37
cckACKO ES YPS_37
SBRM1 ES YPS_37
SB1003 LS YPS_37
SB1003 ES YPS_30
ALS1 ES YPS_37
SB1003 ES YPS_37

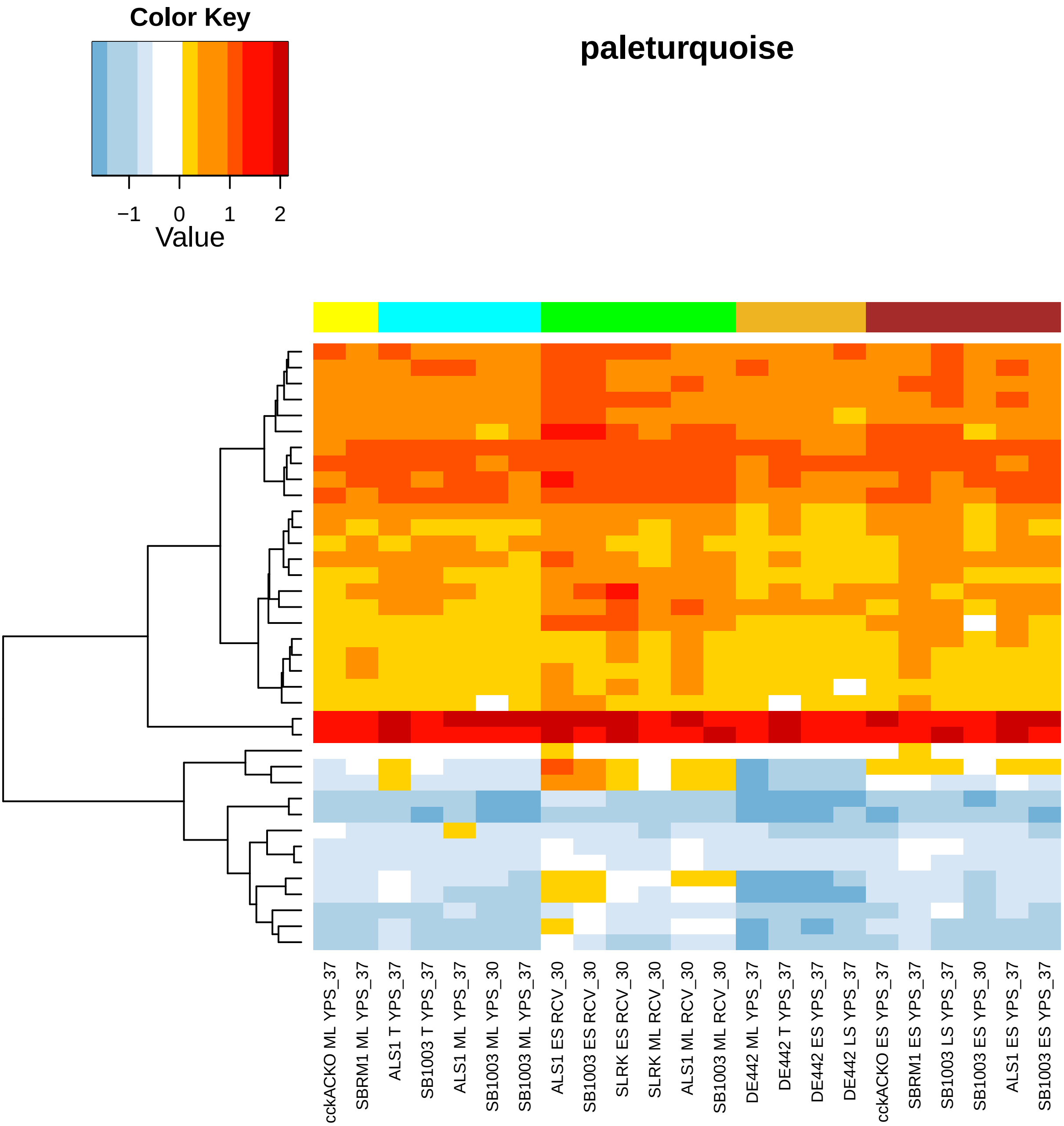

Color Key
−1
0
1
2
Value
paleturquoise
cckACKO ML YPS_37
SBRM1 ML YPS_37
ALS1 T YPS_37
SB1003 T YPS_37
ALS1 ML YPS_37
SB1003 ML YPS_30
SB1003 ML YPS_37
ALS1 ES RCV_30
SB1003 ES RCV_30
SLRK ES RCV_30
SLRK ML RCV_30
ALS1 ML RCV_30
SB1003 ML RCV_30
DE442 ML YPS_37
DE442 T YPS_37
DE442 ES YPS_37
DE442 LS YPS_37
cckACKO ES YPS_37
SBRM1 ES YPS_37
SB1003 LS YPS_37
SB1003 ES YPS_30
ALS1 ES YPS_37
SB1003 ES YPS_37

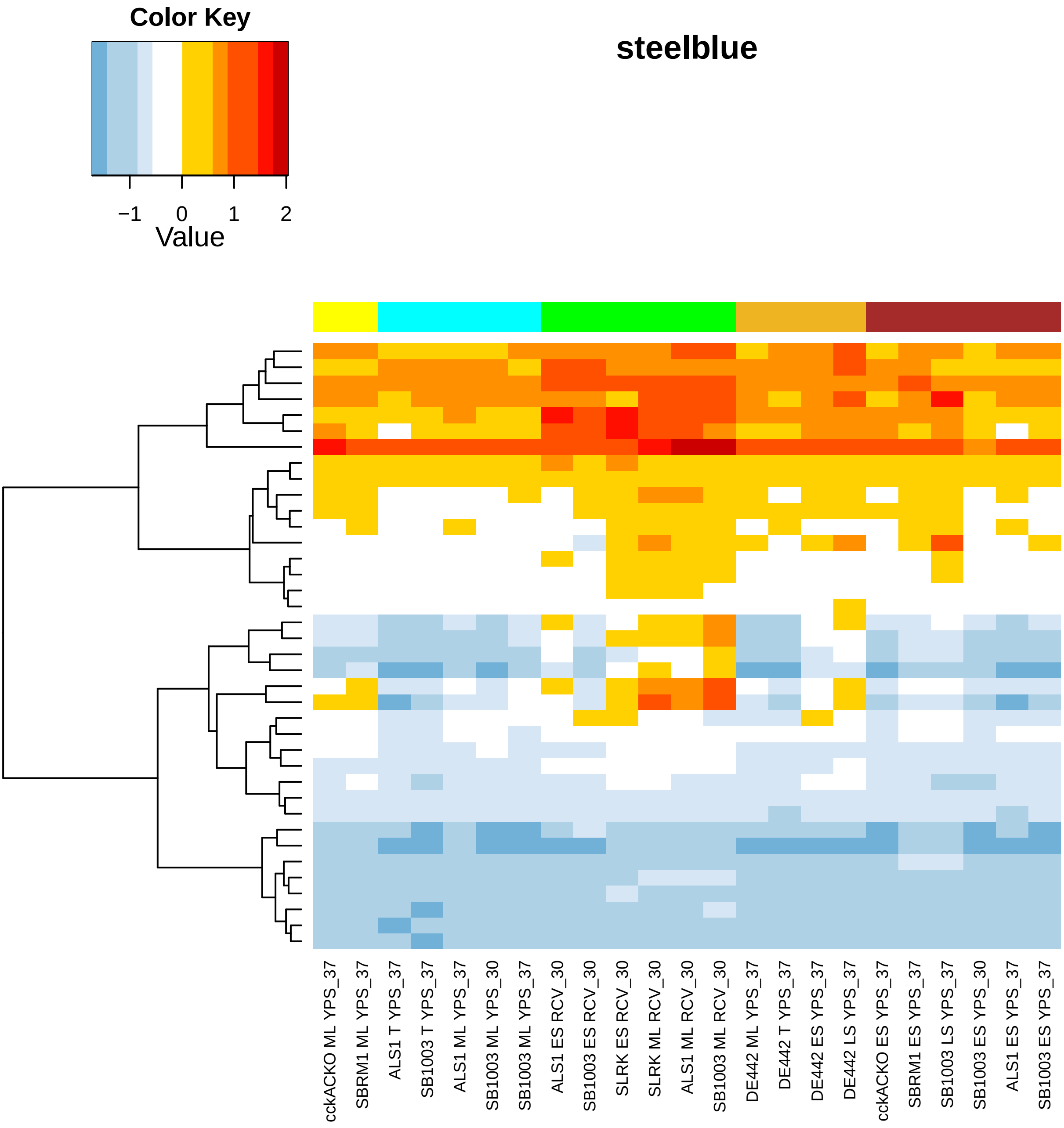

Color Key
−1
0
1
2
Value
steelblue
cckACKO ML YPS_37
SBRM1 ML YPS_37
ALS1 T YPS_37
SB1003 T YPS_37
ALS1 ML YPS_37
SB1003 ML YPS_30
SB1003 ML YPS_37
ALS1 ES RCV_30
SB1003 ES RCV_30
SLRK ES RCV_30
SLRK ML RCV_30
ALS1 ML RCV_30
SB1003 ML RCV_30
DE442 ML YPS_37
DE442 T YPS_37
DE442 ES YPS_37
DE442 LS YPS_37
cckACKO ES YPS_37
SBRM1 ES YPS_37
SB1003 LS YPS_37
SB1003 ES YPS_30
ALS1 ES YPS_37
SB1003 ES YPS_37

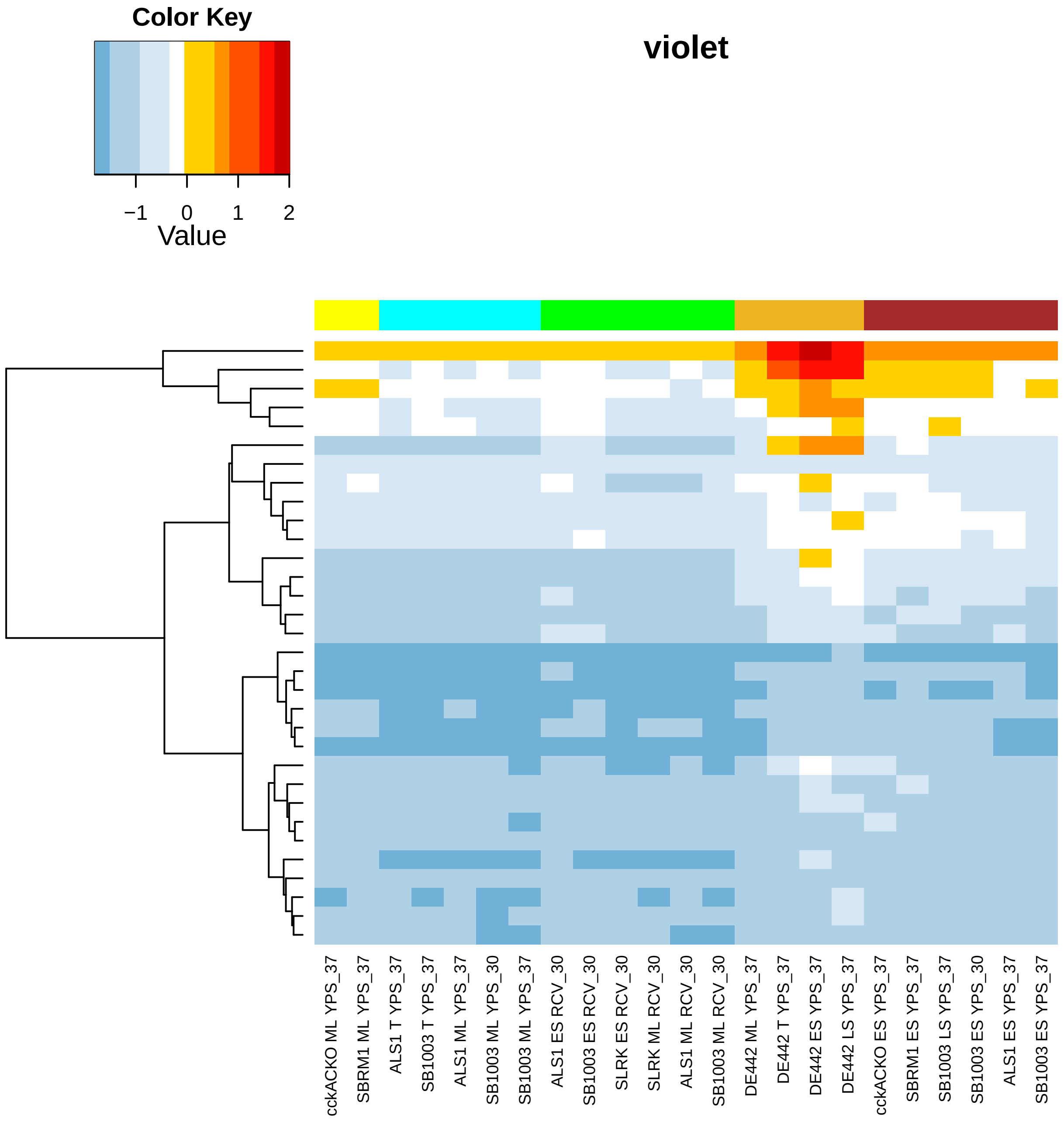
Color Key
−1
0
1
2
Value
violet
cckACKO ML YPS_37
SBRM1 ML YPS_37
ALS1 T YPS_37
SB1003 T YPS_37
ALS1 ML YPS_37
SB1003 ML YPS_30
SB1003 ML YPS_37
ALS1 ES RCV_30
SB1003 ES RCV_30
SLRK ES RCV_30
SLRK ML RCV_30
ALS1 ML RCV_30
SB1003 ML RCV_30
DE442 ML YPS_37
DE442 T YPS_37
DE442 ES YPS_37
DE442 LS YPS_37
cckACKO ES YPS_37
SBRM1 ES YPS_37
SB1003 LS YPS_37
SB1003 ES YPS_30
ALS1 ES YPS_37
SB1003 ES YPS_37

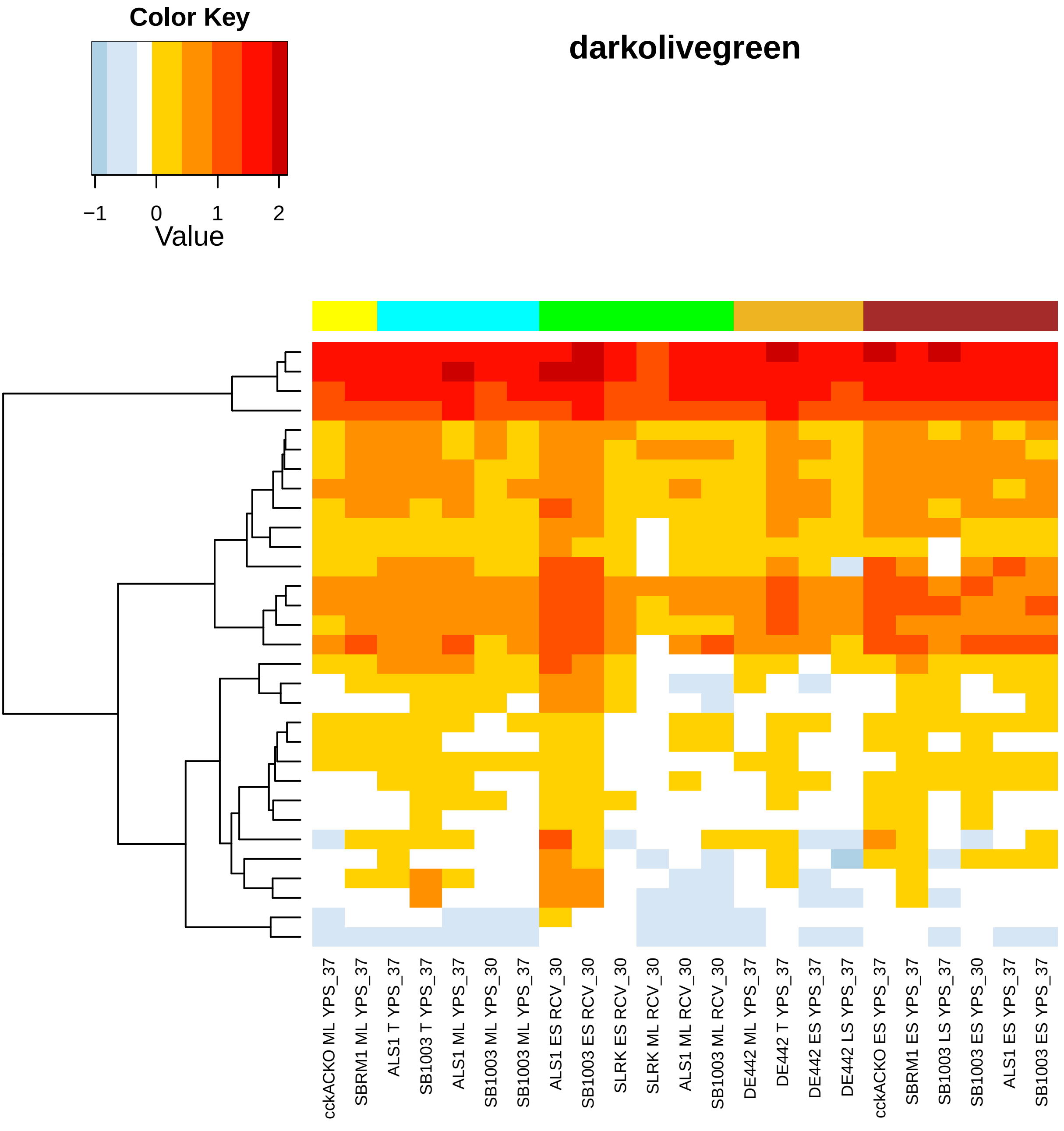

Color Key
−1
0
1
2
Value
darkolivegreen
cckACKO ML YPS_37
SBRM1 ML YPS_37
ALS1 T YPS_37
SB1003 T YPS_37
ALS1 ML YPS_37
SB1003 ML YPS_30
SB1003 ML YPS_37
ALS1 ES RCV_30
SB1003 ES RCV_30
SLRK ES RCV_30
SLRK ML RCV_30
ALS1 ML RCV_30
SB1003 ML RCV_30
DE442 ML YPS_37
DE442 T YPS_37
DE442 ES YPS_37
DE442 LS YPS_37
cckACKO ES YPS_37
SBRM1 ES YPS_37
SB1003 LS YPS_37
SB1003 ES YPS_30
ALS1 ES YPS_37
SB1003 ES YPS_37

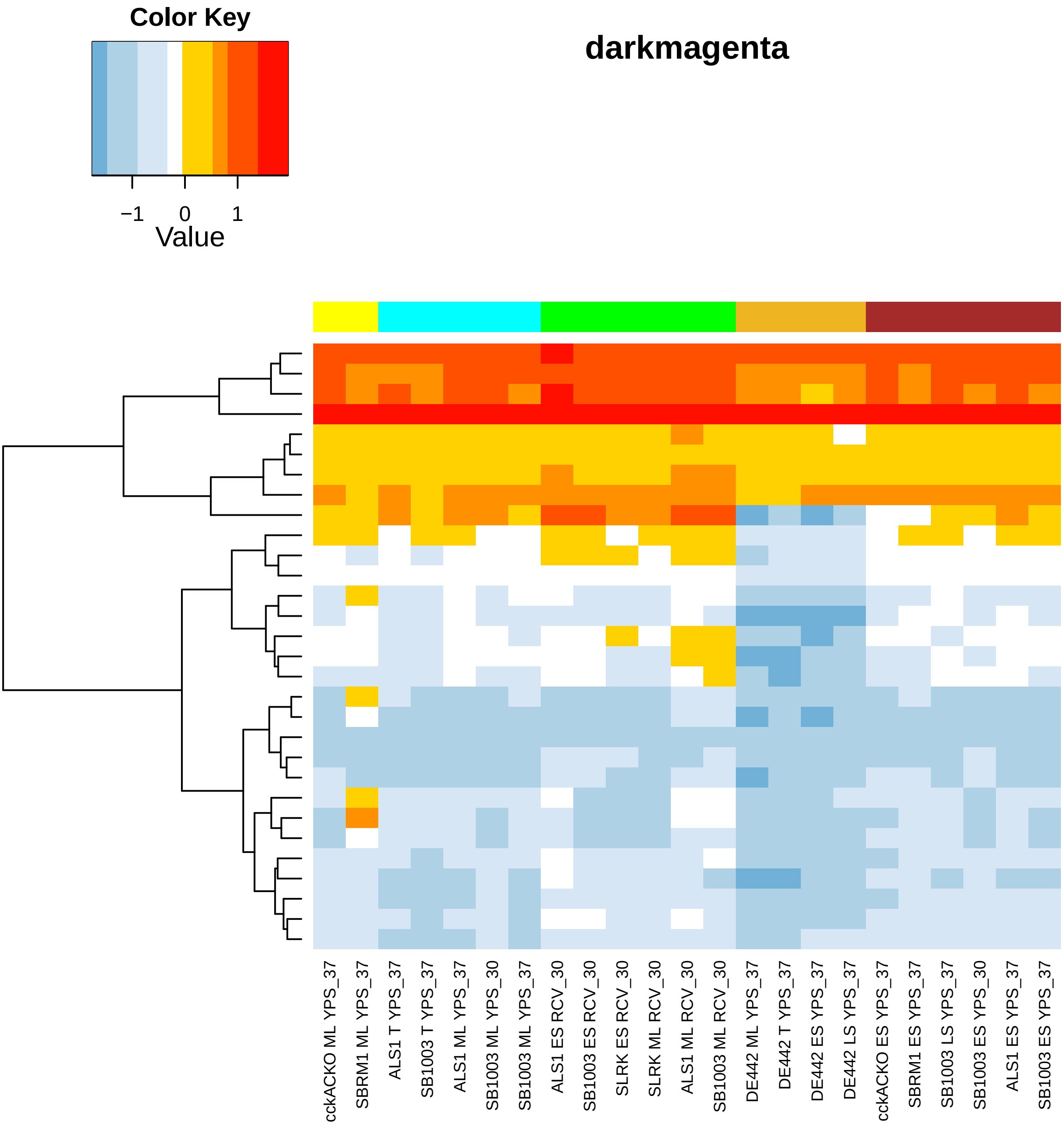
Color Key
−1
0
1
Value
darkmagenta
cckACKO ML YPS_37
SBRM1 ML YPS_37
ALS1 T YPS_37
SB1003 T YPS_37
ALS1 ML YPS_37
SB1003 ML YPS_30
SB1003 ML YPS_37
ALS1 ES RCV_30
SB1003 ES RCV_30
SLRK ES RCV_30
SLRK ML RCV_30
ALS1 ML RCV_30
SB1003 ML RCV_30
DE442 ML YPS_37
DE442 T YPS_37
DE442 ES YPS_37
DE442 LS YPS_37
cckACKO ES YPS_37
SBRM1 ES YPS_37
SB1003 LS YPS_37
SB1003 ES YPS_30
ALS1 ES YPS_37
SB1003 ES YPS_37

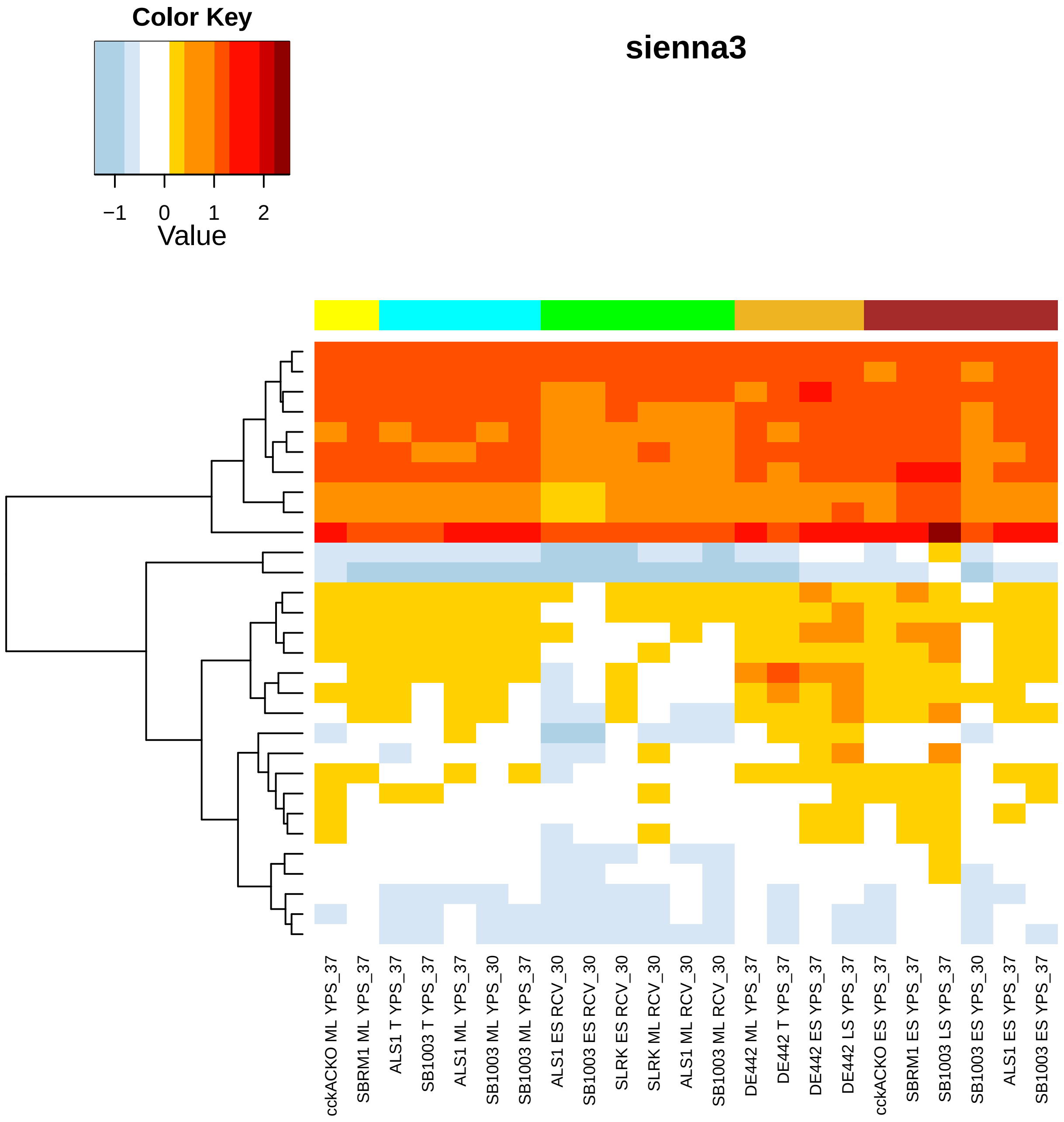
Color Key
−1
0
1
2
Value
sienna3
cckACKO ML YPS_37
SBRM1 ML YPS_37
ALS1 T YPS_37
SB1003 T YPS_37
ALS1 ML YPS_37
SB1003 ML YPS_30
SB1003 ML YPS_37
ALS1 ES RCV_30
SB1003 ES RCV_30
SLRK ES RCV_30
SLRK ML RCV_30
ALS1 ML RCV_30
SB1003 ML RCV_30
DE442 ML YPS_37
DE442 T YPS_37
DE442 ES YPS_37
DE442 LS YPS_37
cckACKO ES YPS_37
SBRM1 ES YPS_37
SB1003 LS YPS_37
SB1003 ES YPS_30
ALS1 ES YPS_37
SB1003 ES YPS_37

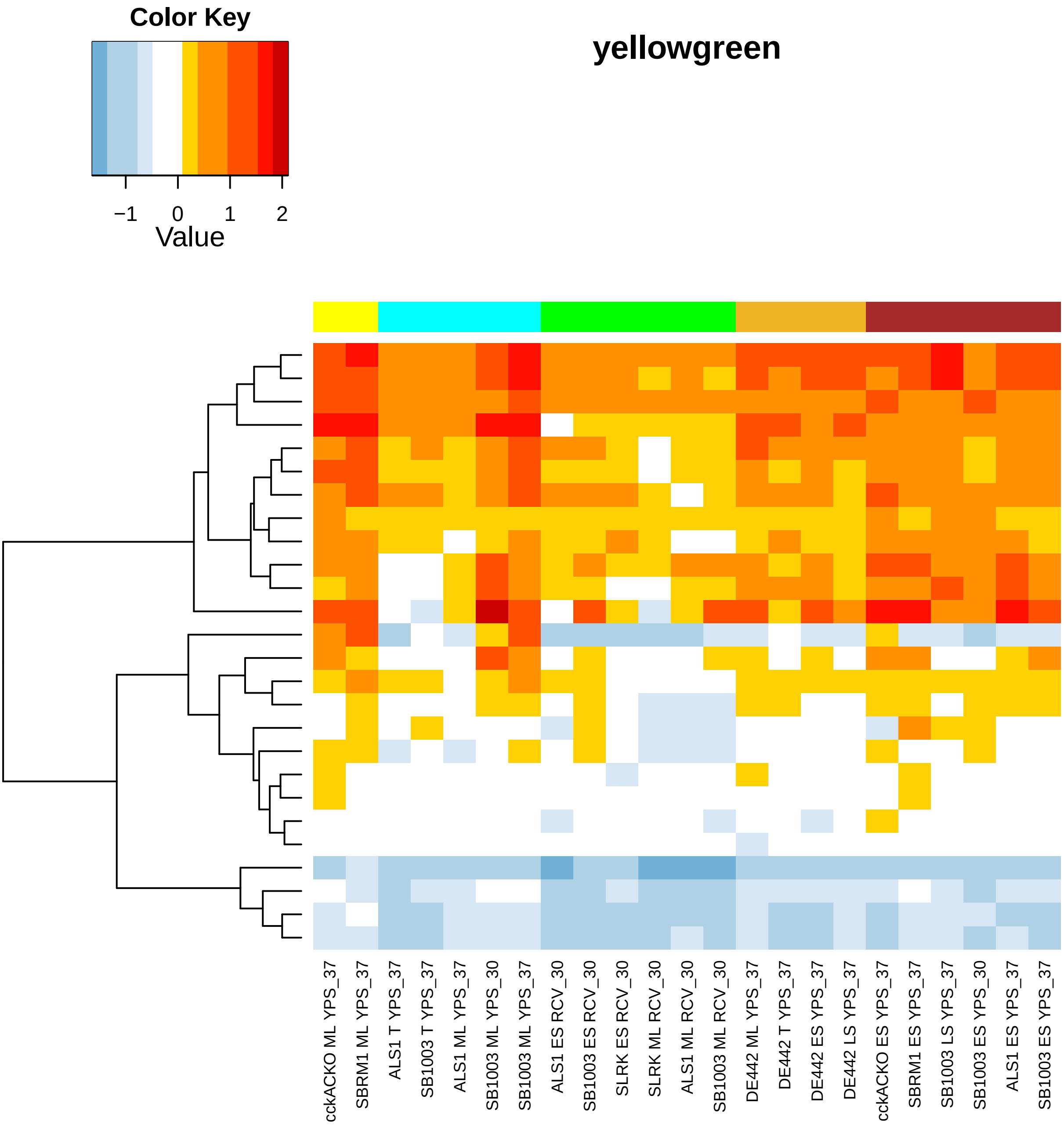

Color Key
−1
0
1
2
Value
yellowgreen
cckACKO ML YPS_37
SBRM1 ML YPS_37
ALS1 T YPS_37
SB1003 T YPS_37
ALS1 ML YPS_37
SB1003 ML YPS_30
SB1003 ML YPS_37
ALS1 ES RCV_30
SB1003 ES RCV_30
SLRK ES RCV_30
SLRK ML RCV_30
ALS1 ML RCV_30
SB1003 ML RCV_30
DE442 ML YPS_37
DE442 T YPS_37
DE442 ES YPS_37
DE442 LS YPS_37
cckACKO ES YPS_37
SBRM1 ES YPS_37
SB1003 LS YPS_37
SB1003 ES YPS_30
ALS1 ES YPS_37
SB1003 ES YPS_37

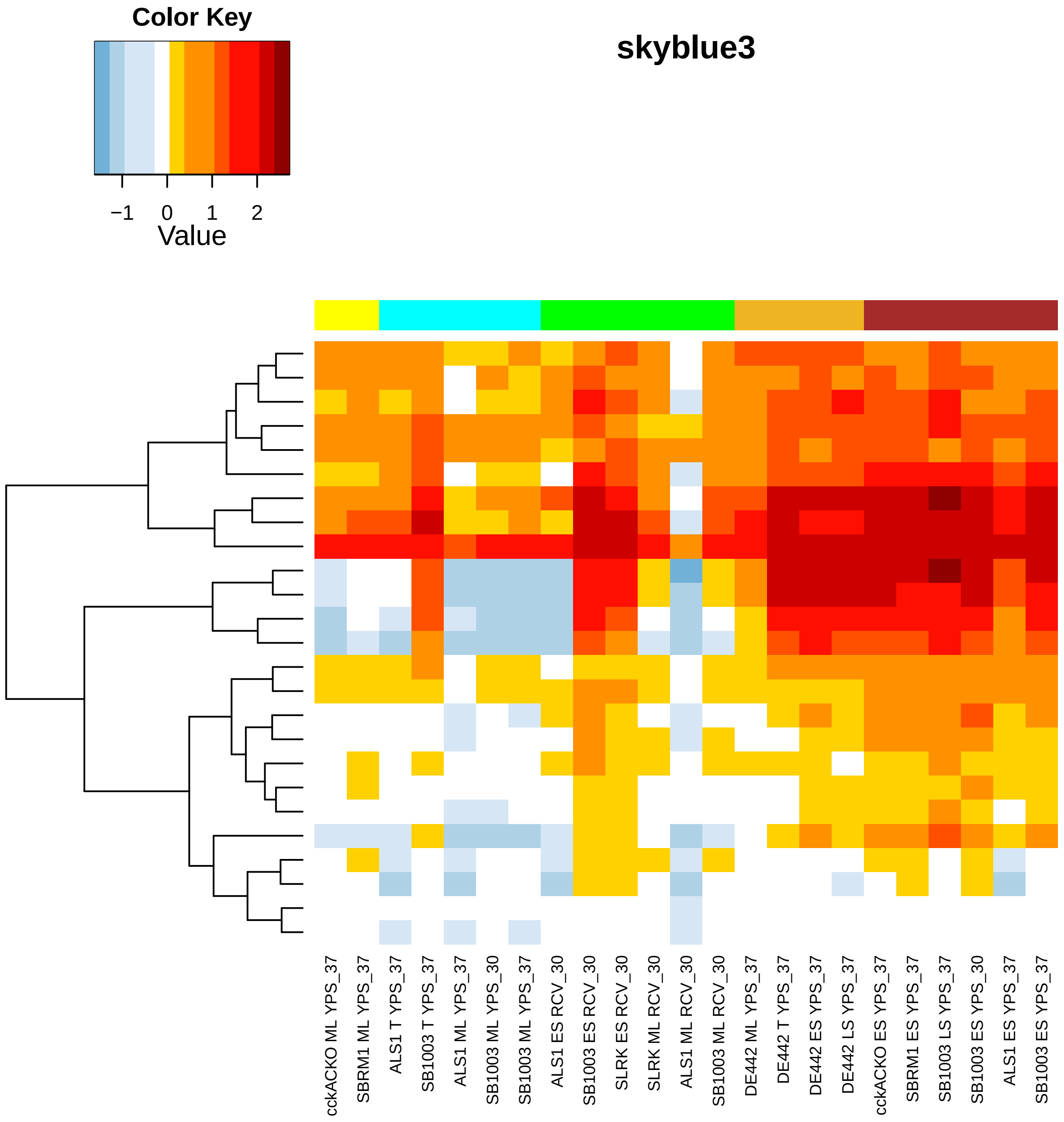
Color Key
−1
0
1
2
Value
skyblue3
cckACKO ML YPS_37
SBRM1 ML YPS_37
ALS1 T YPS_37
SB1003 T YPS_37
ALS1 ML YPS_37
SB1003 ML YPS_30
SB1003 ML YPS_37
ALS1 ES RCV_30
SB1003 ES RCV_30
SLRK ES RCV_30
SLRK ML RCV_30
ALS1 ML RCV_30
SB1003 ML RCV_30
DE442 ML YPS_37
DE442 T YPS_37
DE442 ES YPS_37
DE442 LS YPS_37
cckACKO ES YPS_37
SBRM1 ES YPS_37
SB1003 LS YPS_37
SB1003 ES YPS_30
ALS1 ES YPS_37
SB1003 ES YPS_37

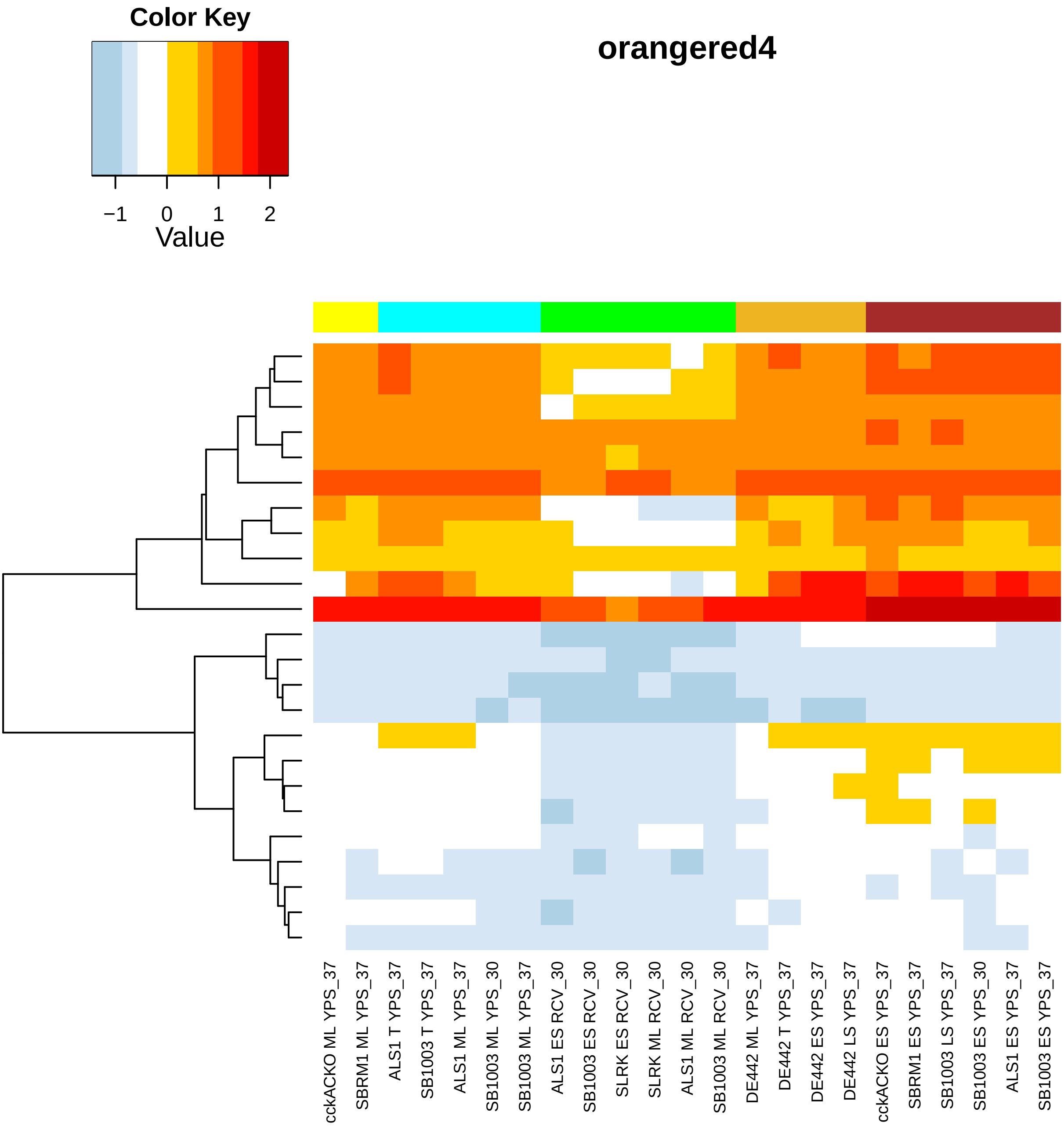

Color Key
−1
0
1
2
Value
orangered4
cckACKO ML YPS_37
SBRM1 ML YPS_37
ALS1 T YPS_37
SB1003 T YPS_37
ALS1 ML YPS_37
SB1003 ML YPS_30
SB1003 ML YPS_37
ALS1 ES RCV_30
SB1003 ES RCV_30
SLRK ES RCV_30
SLRK ML RCV_30
ALS1 ML RCV_30
SB1003 ML RCV_30
DE442 ML YPS_37
DE442 T YPS_37
DE442 ES YPS_37
DE442 LS YPS_37
cckACKO ES YPS_37
SBRM1 ES YPS_37
SB1003 LS YPS_37
SB1003 ES YPS_30
ALS1 ES YPS_37
SB1003 ES YPS_37

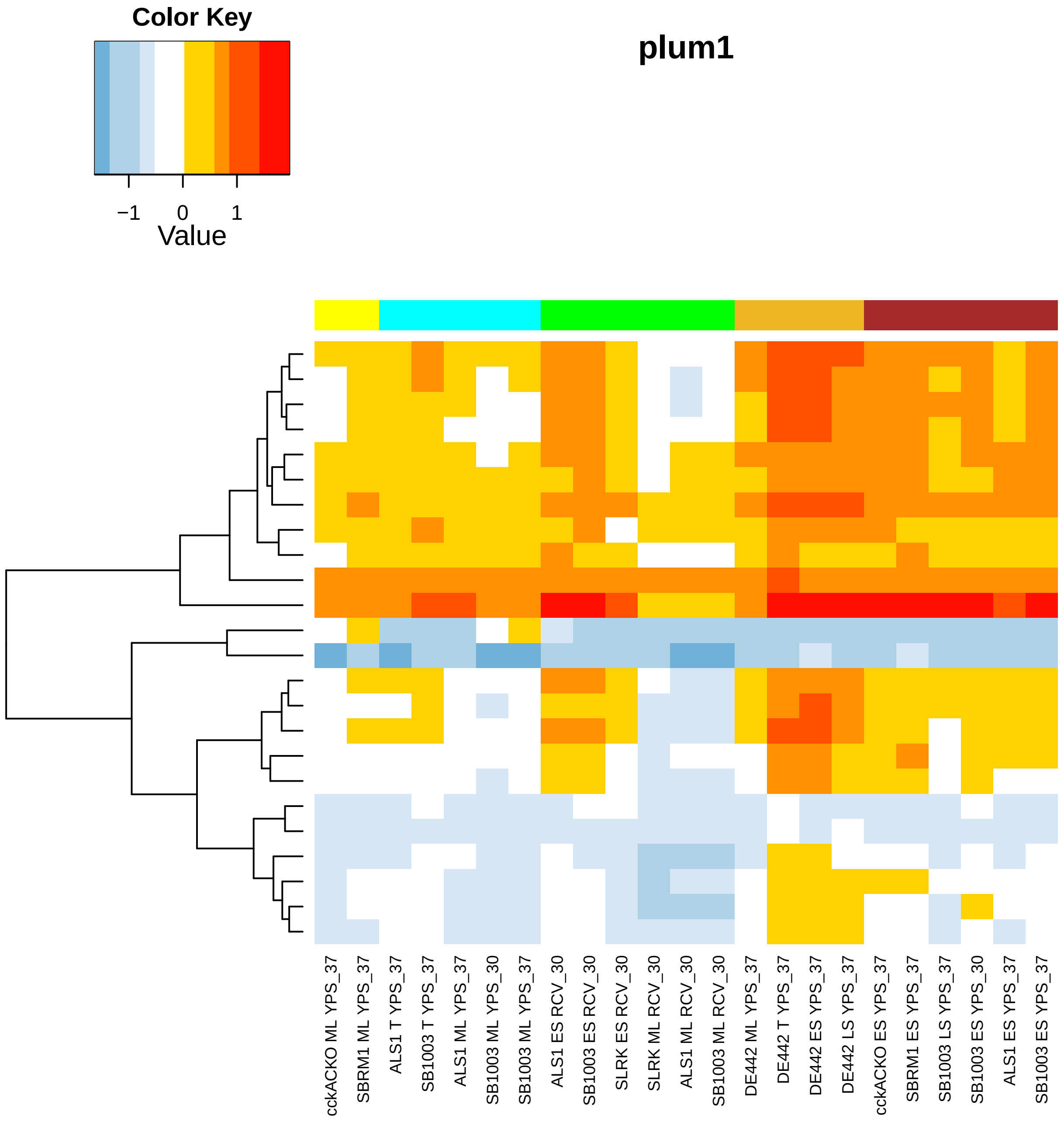
Color Key
−1
0
1
Value
plum1
cckACKO ML YPS_37
SBRM1 ML YPS_37
ALS1 T YPS_37
SB1003 T YPS_37
ALS1 ML YPS_37
SB1003 ML YPS_30
SB1003 ML YPS_37
ALS1 ES RCV_30
SB1003 ES RCV_30
SLRK ES RCV_30
SLRK ML RCV_30
ALS1 ML RCV_30
SB1003 ML RCV_30
DE442 ML YPS_37
DE442 T YPS_37
DE442 ES YPS_37
DE442 LS YPS_37
cckACKO ES YPS_37
SBRM1 ES YPS_37
SB1003 LS YPS_37
SB1003 ES YPS_30
ALS1 ES YPS_37
SB1003 ES YPS_37

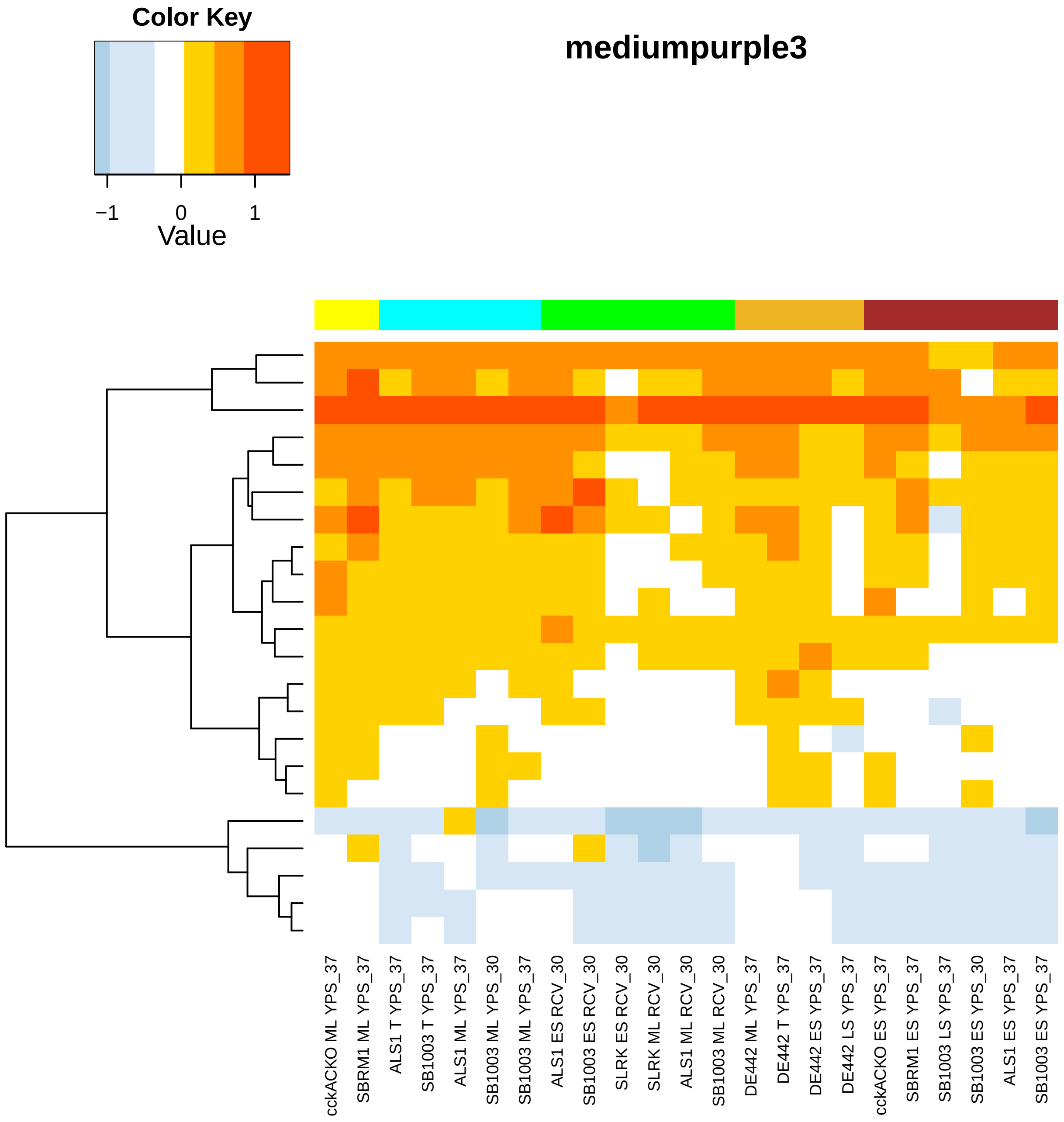

Color Key
−1
0
1
Value
mediumpurple3
cckACKO ML YPS_37
SBRM1 ML YPS_37
ALS1 T YPS_37
SB1003 T YPS_37
ALS1 ML YPS_37
SB1003 ML YPS_30
SB1003 ML YPS_37
ALS1 ES RCV_30
SB1003 ES RCV_30
SLRK ES RCV_30
SLRK ML RCV_30
ALS1 ML RCV_30
SB1003 ML RCV_30
DE442 ML YPS_37
DE442 T YPS_37
DE442 ES YPS_37
DE442 LS YPS_37
cckACKO ES YPS_37
SBRM1 ES YPS_37
SB1003 LS YPS_37
SB1003 ES YPS_30
ALS1 ES YPS_37
SB1003 ES YPS_37

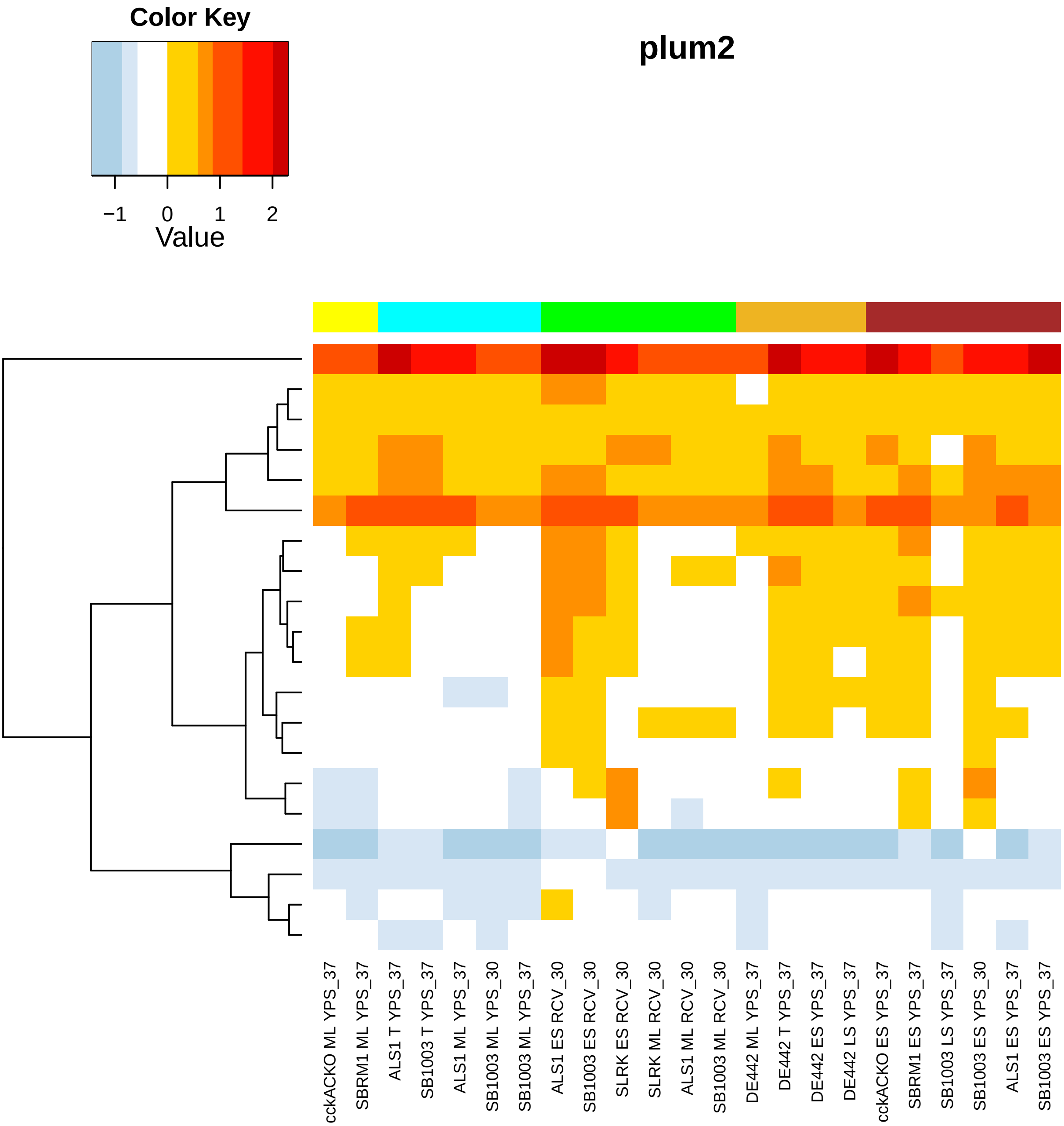

Color Key
−1
0
1
2
Value
plum2
cckACKO ML YPS_37
SBRM1 ML YPS_37
ALS1 T YPS_37
SB1003 T YPS_37
ALS1 ML YPS_37
SB1003 ML YPS_30
SB1003 ML YPS_37
ALS1 ES RCV_30
SB1003 ES RCV_30
SLRK ES RCV_30
SLRK ML RCV_30
ALS1 ML RCV_30
SB1003 ML RCV_30
DE442 ML YPS_37
DE442 T YPS_37
DE442 ES YPS_37
DE442 LS YPS_37
cckACKO ES YPS_37
SBRM1 ES YPS_37
SB1003 LS YPS_37
SB1003 ES YPS_30
ALS1 ES YPS_37
SB1003 ES YPS_37

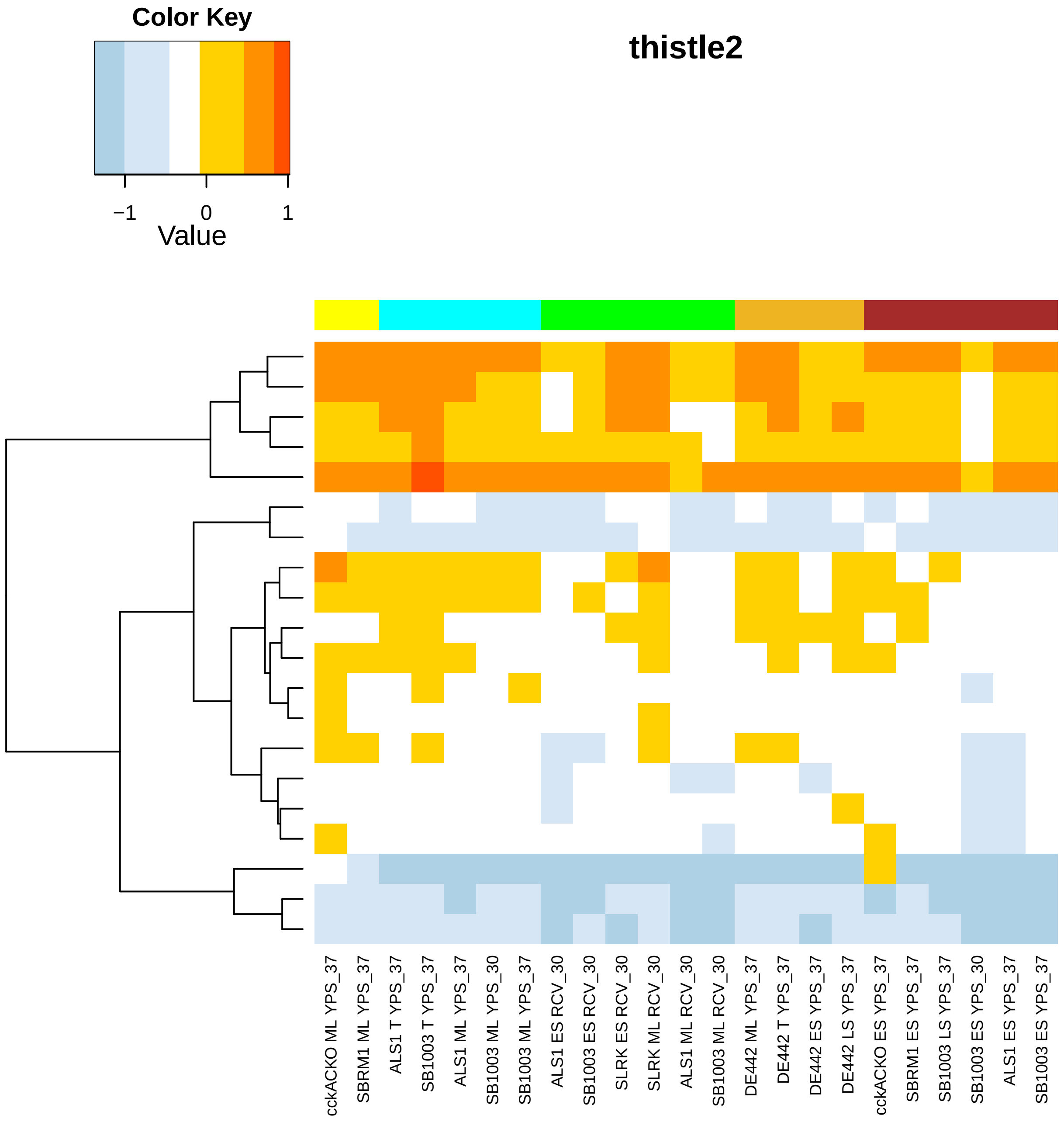
Color Key
−1
0
1
Value
thistle2
cckACKO ML YPS_37
SBRM1 ML YPS_37
ALS1 T YPS_37
SB1003 T YPS_37
ALS1 ML YPS_37
SB1003 ML YPS_30
SB1003 ML YPS_37
ALS1 ES RCV_30
SB1003 ES RCV_30
SLRK ES RCV_30
SLRK ML RCV_30
ALS1 ML RCV_30
SB1003 ML RCV_30
DE442 ML YPS_37
DE442 T YPS_37
DE442 ES YPS_37
DE442 LS YPS_37
cckACKO ES YPS_37
SBRM1 ES YPS_37
SB1003 LS YPS_37
SB1003 ES YPS_30
ALS1 ES YPS_37
SB1003 ES YPS_37

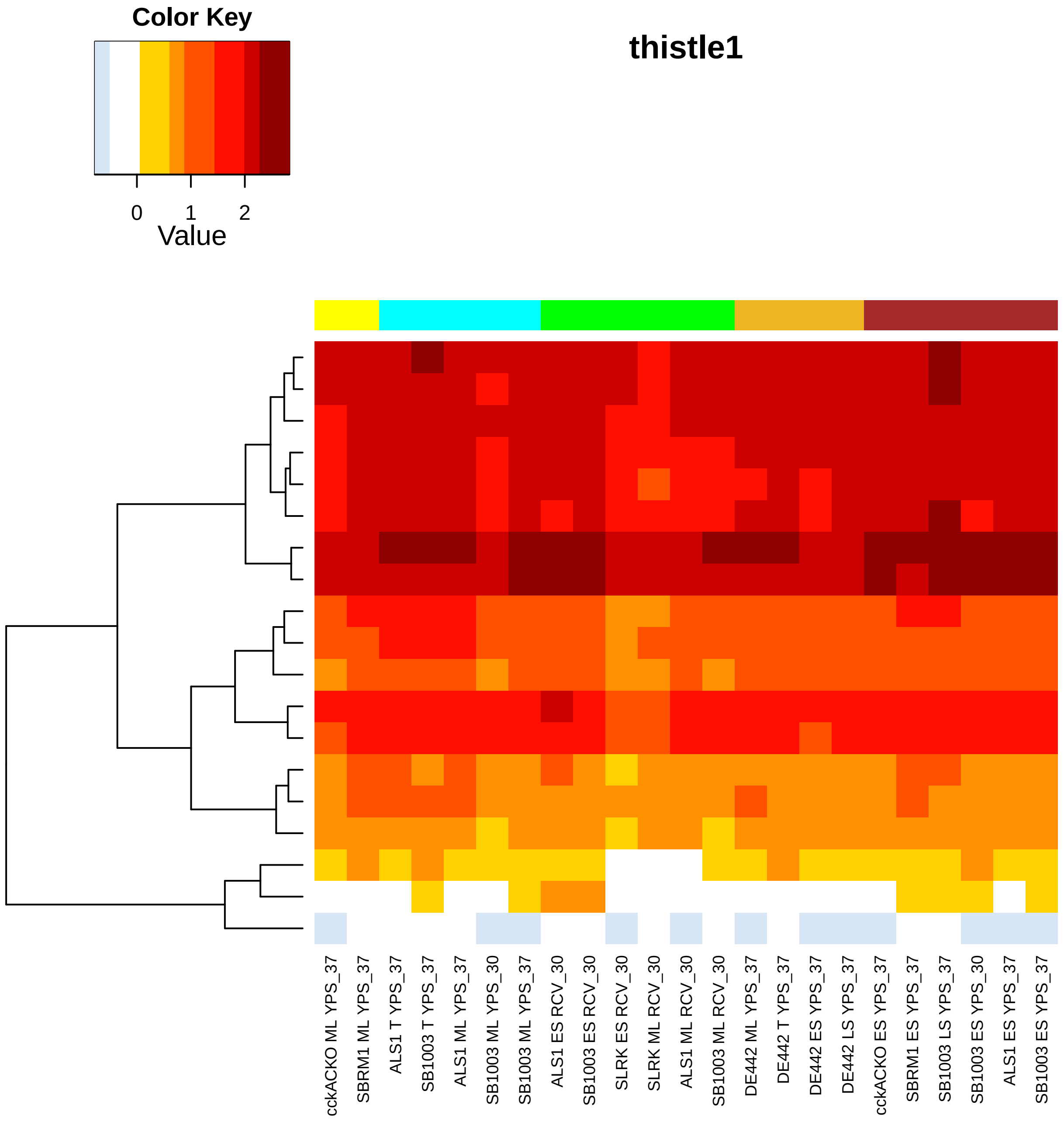

Color Key
0
1
2
Value
thistle1
cckACKO ML YPS_37
SBRM1 ML YPS_37
ALS1 T YPS_37
SB1003 T YPS_37
ALS1 ML YPS_37
SB1003 ML YPS_30
SB1003 ML YPS_37
ALS1 ES RCV_30
SB1003 ES RCV_30
SLRK ES RCV_30
SLRK ML RCV_30
ALS1 ML RCV_30
SB1003 ML RCV_30
DE442 ML YPS_37
DE442 T YPS_37
DE442 ES YPS_37
DE442 LS YPS_37
cckACKO ES YPS_37
SBRM1 ES YPS_37
SB1003 LS YPS_37
SB1003 ES YPS_30
ALS1 ES YPS_37
SB1003 ES YPS_37

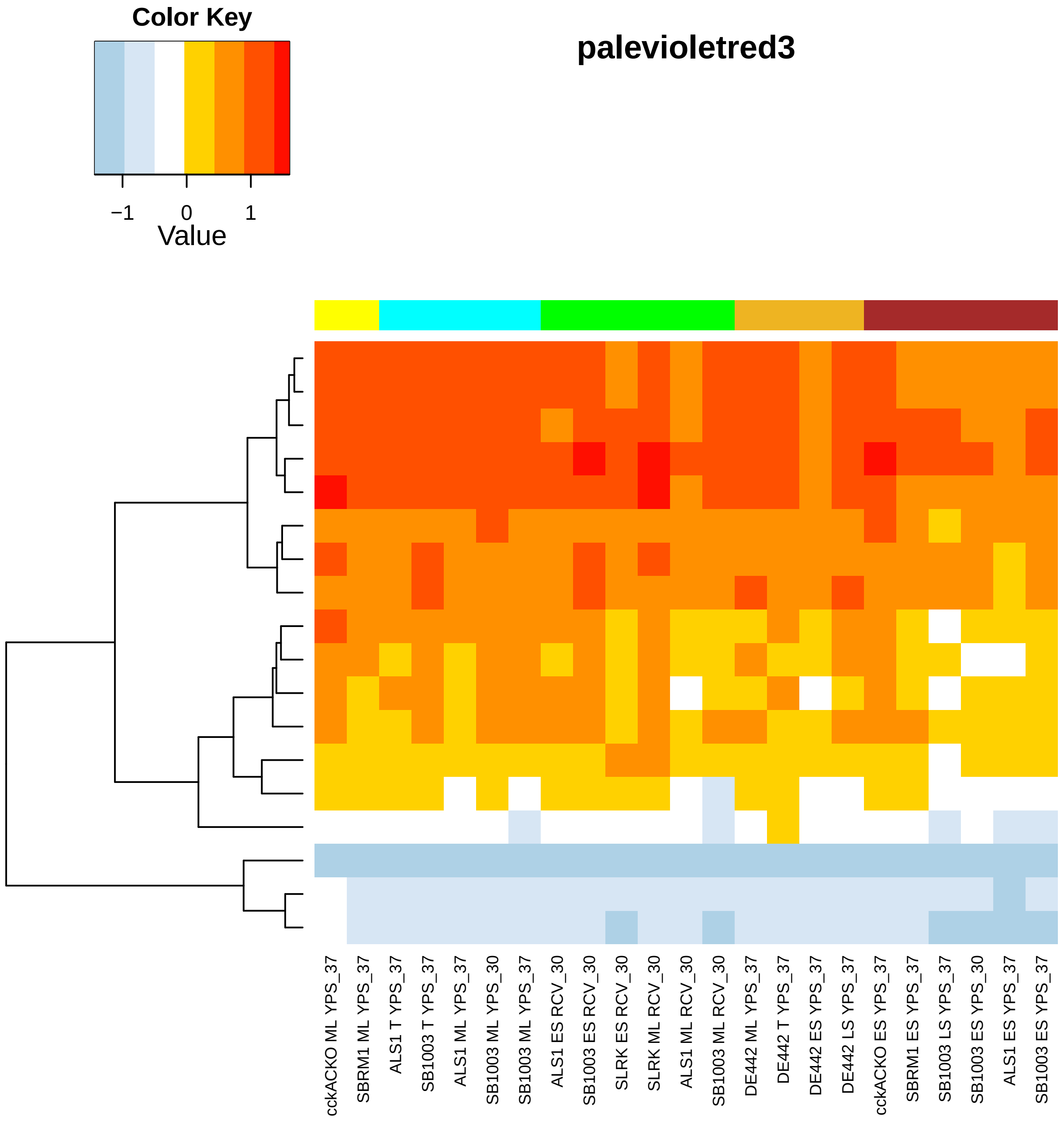
Color Key
−1
0
1
Value
palevioletred3
cckACKO ML YPS_37
SBRM1 ML YPS_37
ALS1 T YPS_37
SB1003 T YPS_37
ALS1 ML YPS_37
SB1003 ML YPS_30
SB1003 ML YPS_37
ALS1 ES RCV_30
SB1003 ES RCV_30
SLRK ES RCV_30
SLRK ML RCV_30
ALS1 ML RCV_30
SB1003 ML RCV_30
DE442 ML YPS_37
DE442 T YPS_37
DE442 ES YPS_37
DE442 LS YPS_37
cckACKO ES YPS_37
SBRM1 ES YPS_37
SB1003 LS YPS_37
SB1003 ES YPS_30
ALS1 ES YPS_37
SB1003 ES YPS_37

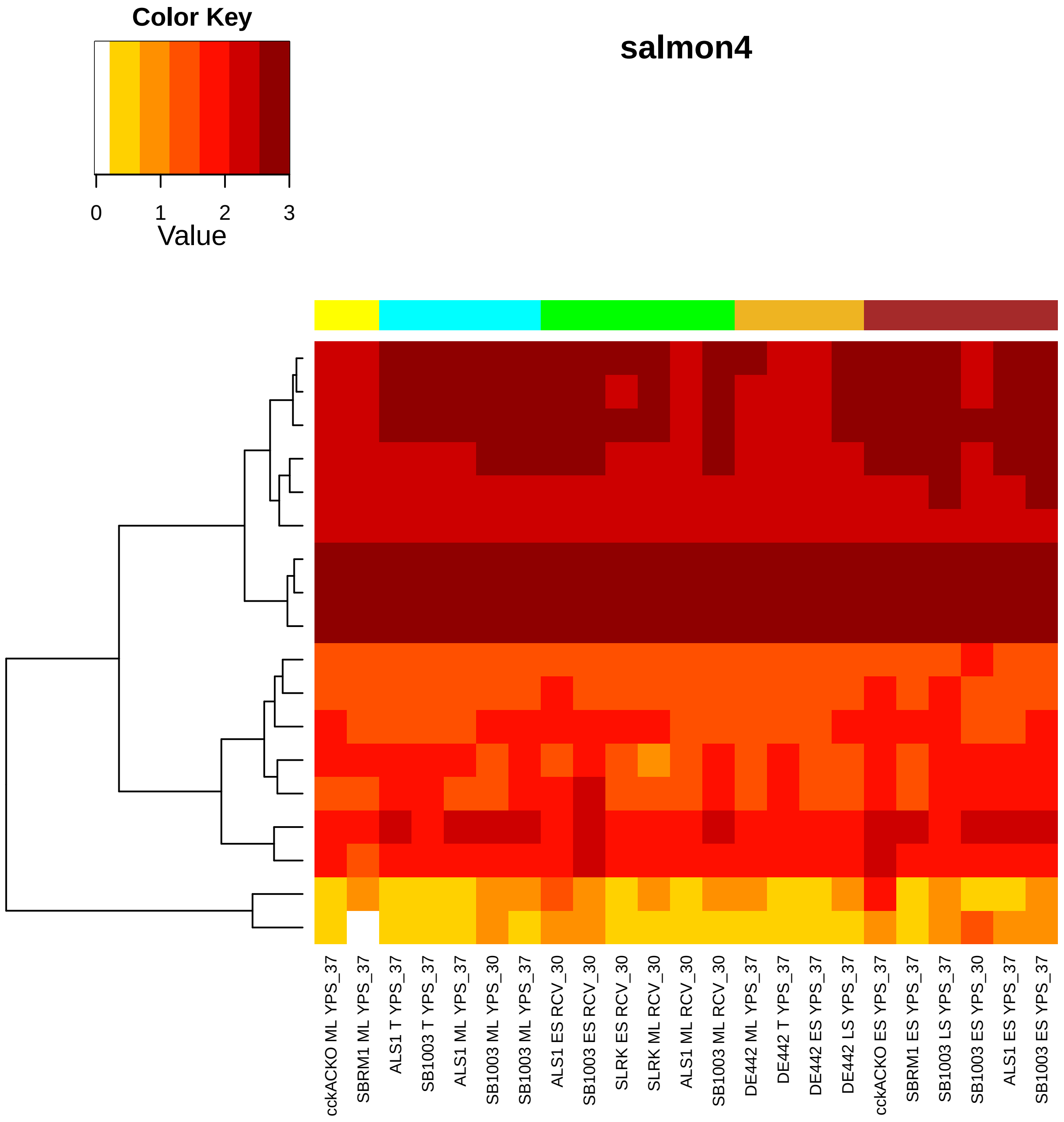

Color Key
0
1
2
3
Value
salmon4
cckACKO ML YPS_37
SBRM1 ML YPS_37
ALS1 T YPS_37
SB1003 T YPS_37
ALS1 ML YPS_37
SB1003 ML YPS_30
SB1003 ML YPS_37
ALS1 ES RCV_30
SB1003 ES RCV_30
SLRK ES RCV_30
SLRK ML RCV_30
ALS1 ML RCV_30
SB1003 ML RCV_30
DE442 ML YPS_37
DE442 T YPS_37
DE442 ES YPS_37
DE442 LS YPS_37
cckACKO ES YPS_37
SBRM1 ES YPS_37
SB1003 LS YPS_37
SB1003 ES YPS_30
ALS1 ES YPS_37
SB1003 ES YPS_37
