# Supplementary material for: Gene co-expression network analysis in Rhodobacter capsulatus and application to comparative expression analysis of Rhodobacter sphaeroides
Source: BMC Genomics. 2014 Aug 28;15(1):730. doi: 10.1186/1471-2164-15-730 (PMC4158056; doi:10.1186/1471-2164-15-730)

Additional file 6. Preservation statistics of *R. capsulatus* gene modules in *R. capsulatus* proteomics data. The size of each bubble represents the module size in the data subset used to assess module preservation (i.e. the number of genes present in the protein abundance dataset). The horizontal line indicates the Zsummary.pres thresholds for low to moderate evidence of conservation (>2). Two *R. capsulatus* modules, blue and brown, were conserved in the protein abundance data. **A.** Module preservation as a function of module quality. The vertical line indicates the Zsummary.qual threshold (>2) for robustness of the mRNA modules in the data subset based on a permutation test. Most modules' Zsummary.qual values are above this threshold. **B.** Relationship between the two preservation statistics, Zsummary.qual and medianRank. Lower medianRank values indicate higher preservation.

A

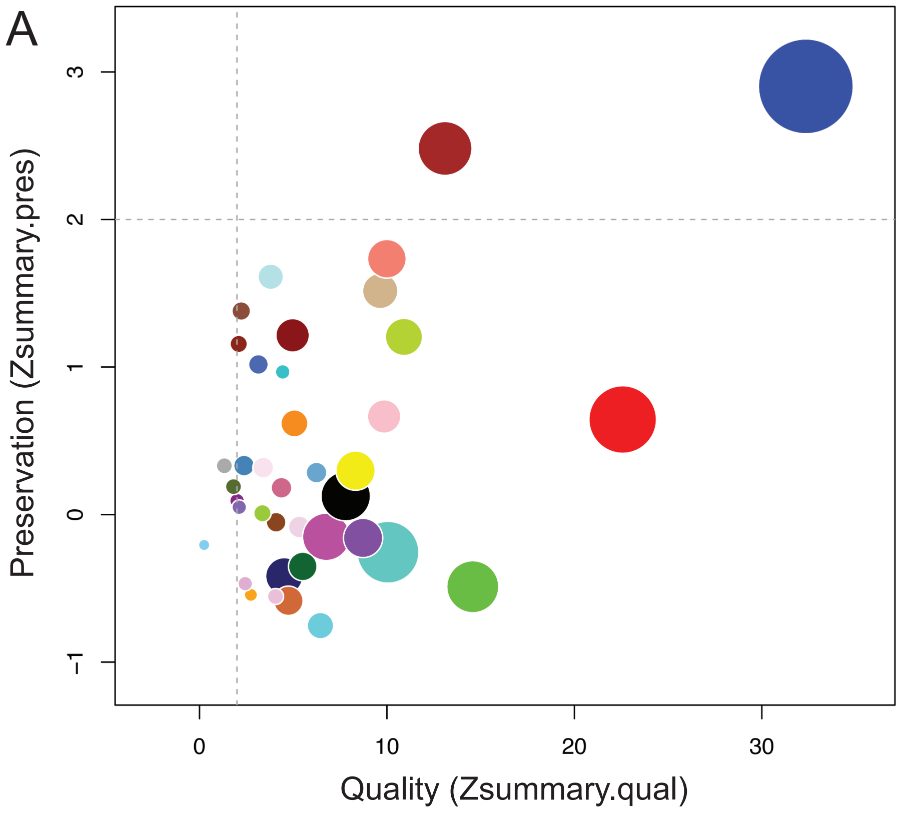

B

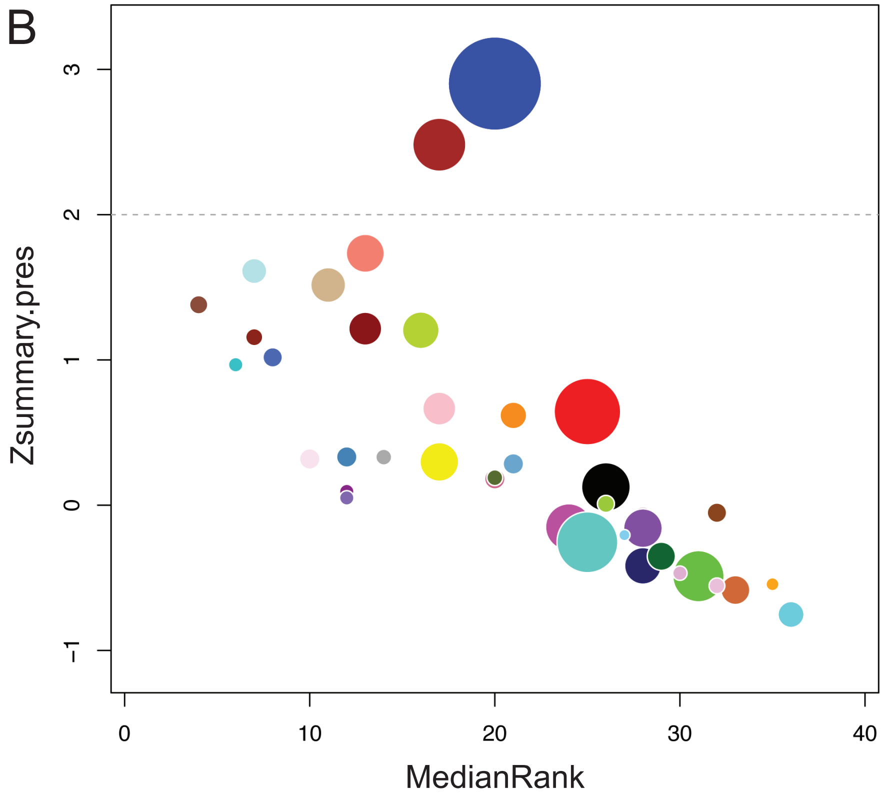

Supplement: Supplementary file 6 — Additional file 6: Preservation statistics of R. capsulatus gene modules in R. capsulatus proteomics data. Module preservation as a function of module quality and relationship between the two preservation statistics, Zsummary.qual and medianRank. (PDF 784 KB) [file 12864_2014_6415_MOESM6_ESM.pdf]
